# DamID transcriptional profiling identifies the Snail/Scratch transcription factor Kahuli as an Alk target in the *Drosophila* visceral mesoderm

**DOI:** 10.1242/dev.199465

**Published:** 2021-12-14

**Authors:** Patricia Mendoza-Garcia, Swaraj Basu, Sanjay Kumar Sukumar, Badrul Arefin, Georg Wolfstetter, Vimala Anthonydhason, Linnea Molander, Ezgi Uçkun, Henrik Lindehell, Cristina Lebrero-Fernandez, Jan Larsson, Erik Larsson, Mats Bemark, Ruth H. Palmer

**Affiliations:** 1 Department of Medical Biochemistry and Cell Biology, Institute of Biomedicine, University of Gothenburg, SE-405 30 Gothenburg, Sweden; 2 Department of Molecular Biology, Umeå University, SE-901 87 Umeå, Sweden; 3 Department of Microbiology and Immunology, Institute of Biomedicine, University of Gothenburg, SE-405 30 Gothenburg, Sweden; 4 Department of Clinical Immunology and Transfusion Medicine, Sahlgrenska University Hospital, Region Västra Götaland, SE-41346 Gothenburg, Sweden

**Keywords:** Single cell, ChIP, Jelly belly, Signaling, TaDa, Midgut constriction, ETS, Pointed

## Abstract

Development of the *Drosophila* visceral muscle depends on Anaplastic Lymphoma Kinase (Alk) receptor tyrosine kinase (RTK) signaling, which specifies founder cells (FCs) in the circular visceral mesoderm (VM). Although Alk activation by its ligand Jelly Belly (Jeb) is well characterized, few target molecules have been identified. Here, we used targeted DamID (TaDa) to identify Alk targets in embryos overexpressing Jeb versus embryos with abrogated Alk activity, revealing differentially expressed genes, including the Snail/Scratch family transcription factor *Kahuli* (*Kah*). We confirmed *Kah* mRNA and protein expression in the VM, and identified midgut constriction defects in *Kah* mutants similar to those of *pointed* (*pnt*). ChIP and RNA-Seq data analysis defined a Kah target-binding site similar to that of Snail, and identified a set of common target genes putatively regulated by Kah and Pnt during midgut constriction. Taken together, we report a rich dataset of Alk-responsive loci in the embryonic VM and functionally characterize the role of Kah in the regulation of embryonic midgut morphogenesis.

## Abstract (precis)

**Summary:** Using targeted DamID (TaDa) to reveal Alk targets in the *Drosophila* visceral mesoderm identified the Kahuli (Kah) transcription factor, which plays a role in the regulation of embryonic midgut morphogenesis.

## INTRODUCTION

Receptor tyrosine kinase (RTK) signaling enables transduction of extracellular signals into the cell and is essential in a wide range of developmental processes. Ligand-dependent RTK activation is conserved among metazoans, leading to engagement of signal transduction adaptor proteins, serine/threonine kinases and transcription factors (TFs) that regulate gene expression and promote a wide range of intracellular responses. In *Drosophila melanogaster*, the Anaplastic Lymphoma Kinase (Alk) RTK and its ligand Jelly Belly (Jeb), are involved in the development of the visceral mesoderm (VM), where they drive a signaling pathway required for specification of muscle founder cells (FCs) (Englund et al., 2003; Lee et al., 2003; Stute et al., 2004). Ligand-stimulated activation of Alk acts through the guanosine triphosphatase Ras, the scaffold protein connector enhancer of kinase suppressor of Ras (Cnk) and Aveugle/Hyphen (Ave/Hyp) to drive the mitogen-activated protein kinase/extracellular signal-regulated kinase (MAPK/ERK) pathway (Englund et al., 2003; Lee et al., 2003; Stute et al., 2004; Wolfstetter et al., 2017).

During *Drosophila* development, the mesoderm is partitioned along the dorso-ventral axis due to inductive inputs from the ectoderm, such as Decapentaplegic (Dpp), which induces high levels of Tinman (Tin) and subsequently Bagpipe (Bap), leading to VM specification (Frasch, 1995). Early VM consists of naïve Alk-expressing myoblasts that are specified to become either FCs or fusion-competent myoblasts (FCMs). FC specification requires activation of Alk signaling by Jeb secreted from the adjacent somatic mesoderm (Englund et al., 2003; Lee et al., 2003; Stute et al., 2004). After specification, FCs fuse with FCMs, forming binucleate myotubes (Campos-Ortega and Hartenstein, 1997; Klapper et al., 2002; Lee et al., 2006; Martin et al., 2001; Poulson, 1950). Fusion is required for the formation of circular visceral muscles, upon which the longitudinal muscle precursors migrate, forming a web of interconnected muscles surrounding the midgut endoderm (Georgias et al., 1997; Klapper et al., 2002; Kusch and Reuter, 1999; Martin et al., 2001; Rudolf et al., 2014). Although Alk is expressed throughout the VM, only the ventral-most row of cells are exposed to Jeb (Englund et al., 2003; Lee et al., 2003; Lorén et al., 2001; Stute et al., 2004). Alk signaling regulates transcription of FC-specific genes, including *Hand*, *optomotor-blind-related-gene-1* (*org-1*) and *kin of irre* (*kirre*) (Englund et al., 2003; Lee et al., 2003; Stute et al., 2004; Varshney and Palmer, 2006). Animals devoid of FCs do not undergo visceral myoblast fusion and the gut musculature fails to develop (Englund et al., 2003; Lee et al., 2003; Lorén et al., 2001; Stute et al., 2004). The influence of Alk signaling on later events in visceral myogenesis is unclear; however, Alk activity is required for visceral *decapentaplegic* (*dpp*) expression in the VM (Shirinian et al., 2007).

Although Alk has been widely studied during *Drosophila* embryogenesis, assaying transcriptional effects specifically in the VM is challenging using traditional transcriptomics, and few Alk transcriptional targets have been described. We used Targeted DamID (TaDa) to determine genome-wide Alk-regulated transcriptional events in the embryonic VM. TaDa exploits the activity of bacterial DNA adenine methyltransferase (Dam) fused to any protein of interest to determine cell type-specific DNA-binding profiles, and has previously been used with RNA polymerases, TFs and histone modifiers, among others (Aughey and Southall, 2016). TaDa can further be refined to address DNA-binding profiles in specific tissues and time points using the well-established GAL4/UAS expression system (Brand and Perrimon, 1993; Southall et al., 2013). This tissue-specific approach revealed known and previously unidentified Alk VM target genes. Among these, we validated the Snail/Scratch family TF Kahuli (Kah) as a previously unreported VM target of Alk. Loss of Alk signaling resulted in reduced *Kah* mRNA expression in FCs, while activation of Alk increased *Kah* expression. To characterize *Kah* function, we generated *Kah* loss-of-function mutants, which fail to form the first midgut constriction at later embryonic stages. We show that this defect in *Kah* mutants is similar to that previously described for *pnt* mutants, suggesting that Kah and Pnt may function together to regulate this process. Publicly available ChIP datasets for Kah and Pnt revealed a number of common targets, reinforcing the hypothesis that Kah and Pnt work together in midgut morphogenesis. Thus, our Alk activity-dependent DamID approach successfully identified a number of Alk-regulated transcriptional targets in the embryonic VM, including the Kah TF that is required for correct midgut constriction.

## RESULTS

### Targeted DamID-derived transcriptional landscape of the *Drosophila* VM

**Fig. 1.**
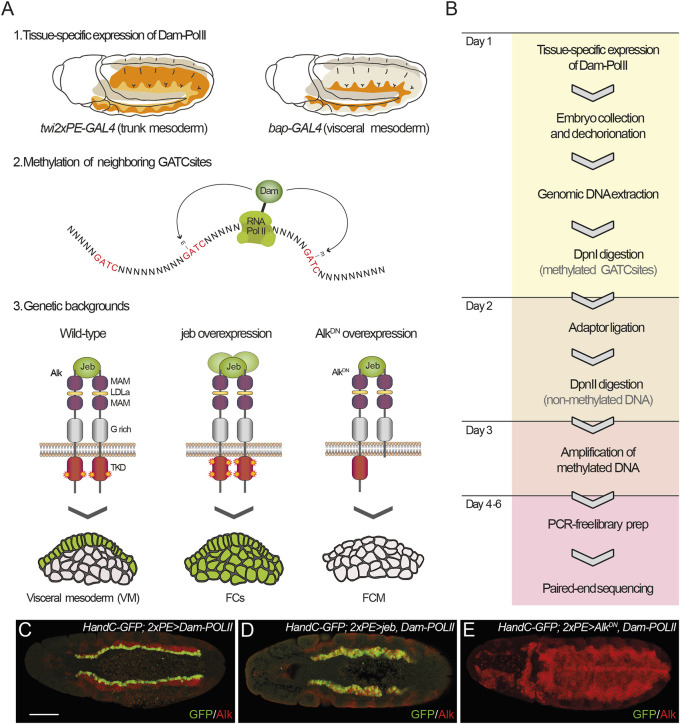
**Transcriptional profiling of Alk targets in the VM by TaDa.** (A) Schematic outlining the TaDa experimental approach. *bap-GAL4* and *twi.2xPE-GAL4* were used to drive tissue-specific expression of Dam-PolII (1), leading to methylation of GATC sequences throughout the genome (2). TaDa analyses were performed in conditions of wild-type, activated (via *jeb* overexpression) and inhibition of Alk signaling (via dominant-negative *Alk.DN* overexpression) (3). (B) Experimental flow-chart from TaDa expression to library preparation and sequencing. (C-E) *HandC-GFP* reporter gene expression in the genetic backgrounds outlined in A3 that were included in TaDa analyses. (C) Wild-type embryos exhibit *HandC-GFP* expression in the ventral-most FC row. (D) Expression of Jeb with *twi2xPE-Gal4* leads to ectopic *HandC-GFP* expression in all VM cells. (E) *HandC-GFP* expression is non-detectable in VM of *twi2xPE-Gal4>AlkDN* embryos. Scale bar: 50 µm.

To characterize Alk-regulated transcriptional activity *in vivo*, we employed TaDa in the embryonic VM in which we genetically manipulated Alk signaling output. We used transgenic *Drosophila* expressing *Dam* methylase fused to *RNA-Pol II* (here *Dam-PolII*) (Southall et al., 2013), driven either generally in the mesoderm (*twi2xPE-GAL4*) or specifically in the VM (*bap-GAL4*), resulting in methylation at GATC sites in targeted tissue (Fig. 1A; Movies 1-4). To manipulate Alk signaling we used combinatorial expression of either *UAS-Jeb*, which leads to ectopic activation of Alk, or *UAS-Alk.EC.MYC*, encoding a MYC-tagged extracellular domain (ECD) of Alk that inhibits Alk signaling in a dominant-negative manner (further referred to as *UAS-Alk.DN*) (Bazigou et al., 2007) (Fig. 1A,C-E). Animals expressing *Dam-PolII* alone in a wild-type background were employed to control for basal Dam-PolII signal (Fig. 1A,C). Stage 10-13 embryos were collected, representing a developmental window during which Alk is activated in future visceral FCs, and experimental sampling was performed in triplicate. Methylated DNA was isolated and digested with the methylation-specific DpnI restriction endonuclease, followed by next-generation sequencing to identify Alk transcriptional targets in the VM (Fig. 1B). Our analysis of this dataset was based on previous pipelines developed for DamID (Maksimov et al., 2016; Tosti et al., 2018). The number of quality reads obtained were comparable between samples and replicates (>15M reads/sample, Fig. S1A). After alignment to the *Drosophila* genome, sequencing depth was >60%, with exception of one sample (Fig. S1B). A high degree of reproducibility was observed between biological replicates overexpressing Alk.DN and Dam-PolII. In contrast, samples expressing Jeb displayed substantial variation (Fig. S1C). Therefore, we employed *twi2xPE-GAL4*- and *bap-Gal4*-driven *UAS-jeb* NGS TaDa datasets for qualitative analyses only.

To assess whether our DamID approach recapitulates transcriptionally active regions of the genome, we performed a meta-analysis of Dam-PolII occupancy, as indicated by GATC associated reads (see Materials and Methods for details), relative to the distance to the closest transcription start site (TSS). When comparing all GATC motifs (Non Dam-PolII) and random regions in the genome to Dam-PolII methylated GATC sites (Dam-PolII), we observed a tendency for methylated GATC sites to accumulate close to TSSs (Fig. S2A). In addition, we compared our DamID results with previously published RNA-seq data from isolated mesoderm cells (NCBI BioProject, PRJEB11879). In agreement with our previous observations, the Dam-PolII-binding profile along all annotated genes is consistent with a mesodermal RNA expression profile (Fig. S2B,C), demonstrating that the Dam-PolII binding in our analyses reflects PolII *in vivo* occupancy.

### TaDa identifies Alk-regulated loci in the *Drosophila* VM

**Fig. 2.**
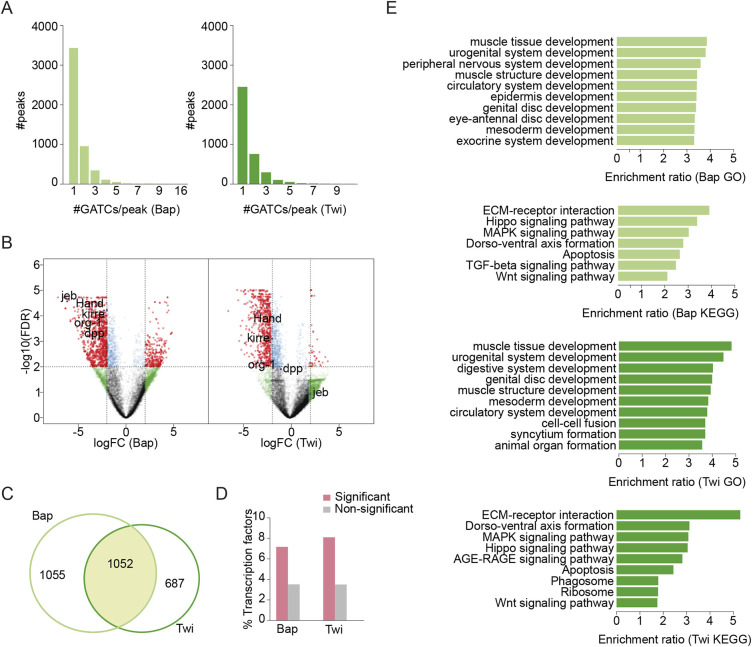
**Significant peaks and associated genes identified by TaDa.** (A-E) LogFC of reads mapped to GATCs obtained by comparing *UAS-Alk.DN* samples against Dam-Pol II samples separately for *bap-GAL4* (Bap) and *twi.2xPE-GAL4* (Twi) samples. Peaks were built by clustering GATC sites at median GATC fragment distance for the *Drosophila* genome. LogFC represents the mean logFC of all GATCs falling inside the peak. (A) Distribution of peaks formed by clustering, expressed as number of GATC sites per peak. (B) Each gene was assigned an overlapping peak with a minimum FDR value, and both logFC and FDR for the assigned peaks are shown as a volcano plot. Genes of interest and known Alk transcriptional targets, such as *Hand*, *kirre*, *org-1* and *dpp*, are indicated. For details see Table S1. (C) Venn diagram indicating the number of genes associated with peaks at FDR<0.01 for bap-GAL4 (bap) and twi.2xPE-GAL4 (Twi) TaDa datasets. (D) Genes associated with Bap and Twi peaks (FDR<0.01) are enriched for TFs, compared with the remaining set of genes in both instances (Fisher test, *P*<2e-16). For details, see Table S1. (E) Enrichment of GO terms and KEGG pathways (FDR<0.05) for genes associated with significant peaks for bap-GAL4 (bap) and *twi.2xPE-GAL4* (Twi) TaDa datasets.

To detect differential gene expression between *Dam-PolII* and *Alk.DN* samples, we clustered neighboring GATC-associated reads, a maximum of 350 bp apart (median GATC fragment distance for the *Drosophila* genome) into peaks (Tosti et al., 2018). Most peaks were associated with a single GATC (Fig. 2A). We then calculated the mean fold change ratios for all GATCs falling into each peak across annotated transcripts (*Alk.DN/Dam-Pol II*), and a false discovery rate (FDR) was assigned to each peak. Each gene along the genome was assigned an overlapping peak with the minimum FDR value, and its logFC and FDR were used for differential expression analysis visualization on a volcano plot (Fig. 2B).

For statistical analyses, a FDR <0.01 was considered significant. In total, we identified significant change in Dam-PolII occupancy on 1739 genes in the *twi.2xPE-GAL4* samples (Twi) and 2107 genes in the *bap-GAL4* samples (Bap), with an overlap of 1052 genes between samples (Fig. 2C). Identified genes included known targets of Alk signaling, such as *Hand*, *org-1*, *kirre* and *dpp* (Fig. 2B, Table S1) (Lee et al., 2003; Lorén et al., 2003; Shirinian et al., 2007; Stute et al., 2004; Varshney and Palmer, 2006), demonstrating that the TaDa approach was successfully able to identify Alk targets in the VM.

Alk signaling controls FC specification in the VM; therefore, we expected transcriptional activation of factors involved in this process. With TaDa, we observed peak enrichment for TFs in both Bap and Twi datasets, including *Hand* and *org-1* (Fig. 2B,D). We also observed genes involved in VM cell fusion, such as *kirre* (upregulated), and *sticks and stones (sns)* and *Verprolin 1 (Vrp1)* (downregulated) (Fig. 2B). Moreover, we identified factors involved in signaling pathways known to be active during development of the mesoderm, musculature and nervous system (Fig. 2E).

**Fig. 3.**
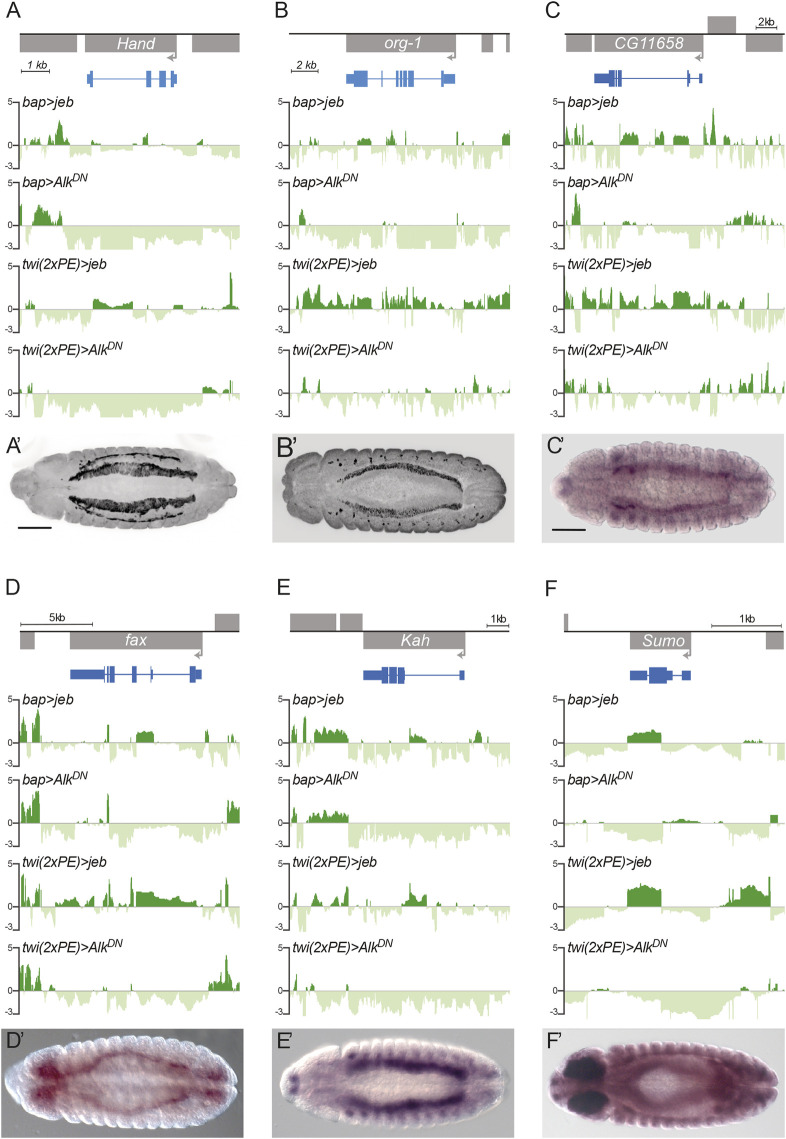
**Validation of selected TaDa-identified gene expression in the VM.** (A-F) Dam-PolII occupancy of selected candidate loci using *bap*- and *twi.2xPE-GAL4* drivers. Known Alk transcriptional targets, *Hand* (A) and *org-1* (B), are shown together with TaDa candidates *CG11658* (C), *fax* (D), *Kah* (E) and *Sumo* (F). *Y*-axes represent logFC between *UAS-Dam-PolII* (reference) and *UAS-Dam-PolII, UAS-jeb* or *UAS-Dam-PolII, UAS-Alk.DN* samples. (A′-F′) Expression patterns of candidate genes at stage 13. HandC-GFP expression (A′) and Org1 protein (B′) are shown, together with mRNA *in situ* of *CG11658* (C′), *fax* (D′), *Kah* (E′) and *Sumo* (F′). Scale bars: 50 µm.

Qualitative analysis of a selection of peak-associated genes with low *P*-values showed differential Dam-PolII occupancy between Jeb and Alk.DN overexpression samples. At individual gene levels, Dam-PolII occupancy revealed similar binding profile dynamics for Jeb (positive peaks) and Alk.DN samples (negative peaks) (Fig. 3A-F). Known Alk targets, such as *Hand* and *org-1*, exhibit highly specific expression in the VM (Fig. 3A,B). Further validation of a selection of highly significant differentially expressed genes by *in situ* showed them to be also actively expressed in the VM. These included *CG11658*, the transmembrane protein *failed axon connections* (*fax*), the *Kahuli* (*Kah*) TF and the SUMO family protein *Sumo* (previously referred to as *smt3*) (Fig. 3C-F). Taken together, our bioinformatics analysis and experimental validation supports TaDa as an effective approach in identification of novel transcriptional regulation events that are potentially downstream of Alk in the VM.

### Alk targets identified by TaDa are enriched in the visceral mesoderm

**Fig. 4.**
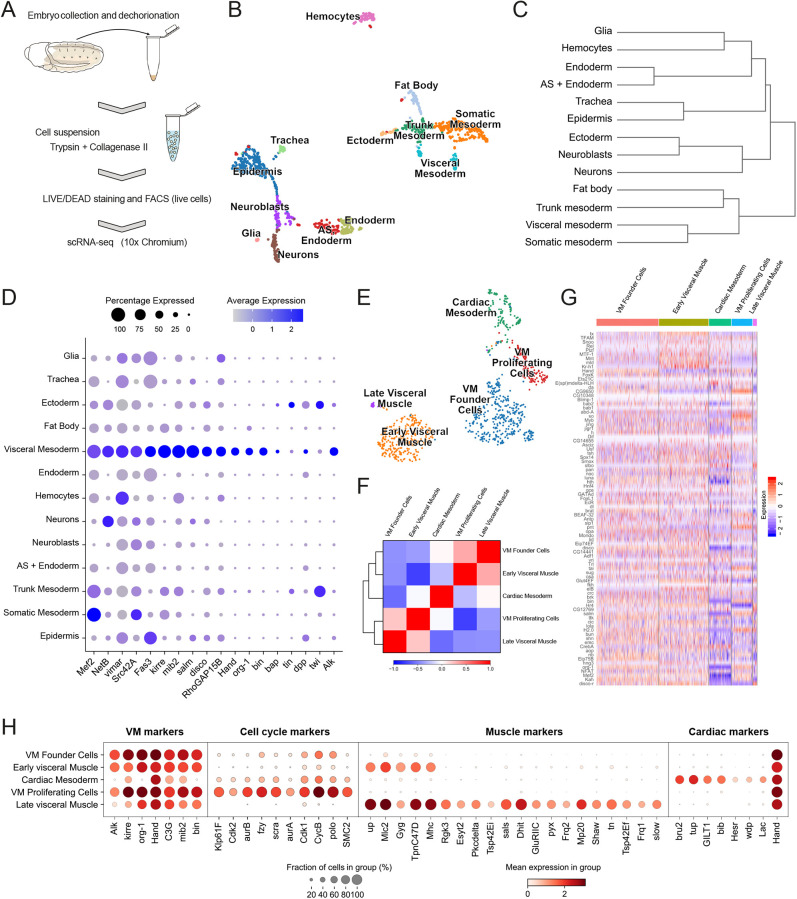
**TaDa-identified Alk targets are enriched in the visceral mesoderm.** (A) Schematic outline of embryonic scRNA-seq workflow. (B) UMAP plot displaying the cellular heterogeneity of whole embryo scRNA-seq as 13 cell clusters. (C) Dendrogram representing the relationship between the clusters. (D) Dot plot highlighting increased expression of factors involved in VM development, such as *bin*, *bap*, *org-1*, *Hand* and *Fas3* in the VM cell population cluster. (E) UMAP projection representing the five clusters of *HandC-GFP*-positive, FACS-sorted cells. (F) Correlation between the clusters across the population of the *HandC-GFP* dataset (Pearson's). (G) Heatmap indicating relative expression of TaDa-identified targets downstream of Alk in *HandC-GFP*-positive cells, highlighting low expression within the cardiac mesoderm population. (H) Dot plot representing the top canonical markers for the *HandC-GFP* scRNA-seq dataset, highlighting VM, cell cycle, muscle and cardiac markers. Expression levels are visualized as mean expression (red gradient, key below), as well as the fraction of cells in a group (dot size, key below).

To validate identified candidates, we employed single-cell RNA-sequencing (scRNA-seq) profiling on cells isolated from stage 10-13 embryos, using live/dead cell markers to isolate living cells through flow cytometric cell sorting (Fig. 4A). After quality control filtering in Seurat R and Scanpy toolkits, 1055 cells from wild-type embryos were further analyzed (Satija et al., 2015; Wolf et al., 2018). Unsupervised clustering of cells based on gene expression profiles identified 13 cell clusters with distinct transcriptional profiles that could be assigned to distinct cell lineages [epidermis, somatic mesoderm, early trunk mesoderm, amnioserosa (AS)+endoderm, neuroblasts, neurons, hemocytes, endoderm, visceral mesoderm, fat body, ectoderm, trachea and glia] (Fig. 4B,C, Fig. S3). When visualized in a two-dimensional uniform manifold approximation and projection (UMAP) plot, clusters distributed into four main groups, one of which comprised clusters of mesodermal origin (Fig. 4B,C, Fig. S3). Within this group, the cluster representing VM was identified by plotting combinatorial gene expression of known factors involved in VM development, such as *biniou* (*bin*), *bagpipe* (*bap*), *org-1*, *Hand*, *Fasciclin 3* (*Fas3*) and *Alk*.

We next analyzed expression levels of TaDa-identified candidates within the VM cluster. However, this was not possible for our whole embryo dataset due to (1) the overall low number of VM cells and (2) the low proportion of VM FCs that precluded a rigorous interrogation of TaDa candidate expression in relation to Alk activity. To achieve this, GFP-expressing cells were purified by flow cytometric cell sorting from *HandC-GFP;twi2xPE-Gal4>UAS-jeb* embryos with an enlarged visceral FC population. After quality filtering, we identified 888 cells that distributed as five clusters based on gene expression profiles (Fig. 4E-H). Four of these clusters exhibited VM marker gene expression. The remaining cluster represented *HandC-GFP-*positive cells of the cardiac mesoderm (CM), as indicated by combinatorial expression of CM-specific genes (Fig. 4G,H). In earlier work, we observed a number of proliferating cells in the VM shortly after FC specification (Popichenko et al., 2013). Further analysis allowed identification of a cluster of cells that strongly expressed both FC- and cell-cycle/cell-proliferation markers, which we termed VM proliferating cells (Fig. 4E,H, Fig. S4). Heatmap analysis revealed an enrichment of TaDa-identified, Alk-regulated genes in the VM that was most prominent in the VM clusters, when compared with the CM cluster (Fig. 4G). Moreover, in agreement with our *in situ* analyses (Fig. 3C-F), we confirmed that *CG11658*, *fax*, *Kah* and *Sumo* were strongly expressed in the VM of our whole embryo scRNA-seq dataset (Fig. S5). Taken together, our scRNA-seq analysis supports our TaDa-based identification of novel Alk signaling targets in the VM.

### *Kahuli* transcription is regulated by Alk signaling in the developing VM

**Fig. 5.**
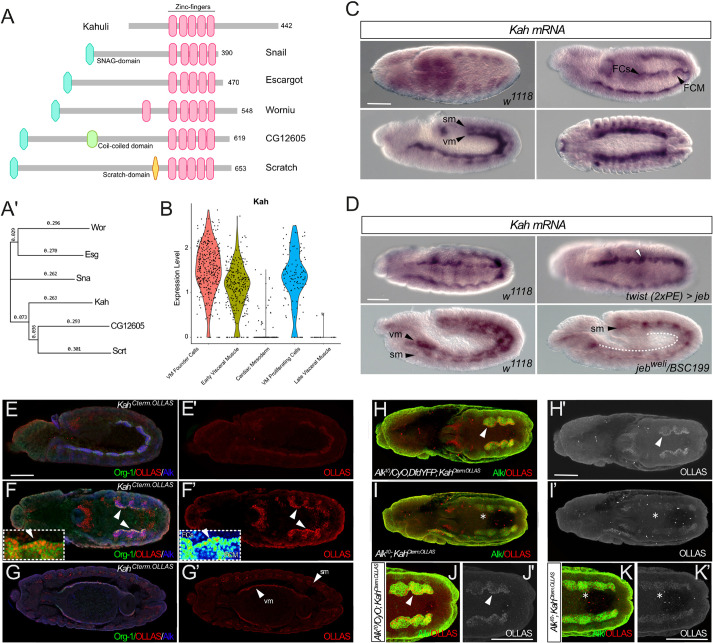
**Kahuli is expressed in developing visceral and somatic mesoderm.** (A) Schematic indicating domain structure of the *Drosophila* Snail/Scratch family members Kahuli, Snail, Escargot, Wornoi, CG12605 and Scratch. SNAIL/Gfi-1 (SNAG, blue), coiled-coil (green) and zinc-finger (pink) domains are shown. (A′) Phylogenetic tree indicating the relationship between Kah and Snail/Scratch family members in *Drosophila*. (B) Violin plots from scRNA-seq analysis of FACS-sorted *Hand-GFP*-expressing cells reveals expression of *Kah* mRNA in VM FC, early visceral muscle and VM proliferating cells, but not in cardiac mesoderm or late visceral muscle. (C) *Kah* transcripts are abundant in SM and VM during embryogenesis, with increased expression levels in the visceral FC row. FC, founder cell; FCM, fusion competent myoblasts; sm, somatic mesoderm; vm, visceral mesoderm. (D) *twi.2xPE-GAL4*-driven Jeb expression results in increased *Kah* expression in VM cells (white arrowhead). Conversely, animals devoid of Jeb/Alk signaling (*jeb^weli^* mutants) lack the strong FC-specific *Kah* expression in the VM (dotted line), while SM expression remains unaltered. (E-G′) Endogenously tagged *Kah^Cterm.OLLAS^* is enriched in, but not exclusive to, visceral FCs (FCs marked by Org-1 in green, OLLAS in red, Alk in blue). (E,E′) *Kah^Cterm.OLLAS^* embryos, lateral view, stage 11/12. (F,F′) *Kah^Cterm.OLLAS^* embryos, dorsal view, stage 11/12. Insets depict higher magnification of Kah^OLLAS^ (F, Kah^OLLAS^ in red, Alk in green; F′, Kah^OLLAS^ in LUT colors). Arrowheads indicate the visceral FC row. (G,G′) *Kah^Cterm.OLLAS^* embryos, dorsal view, stage 13. *Kah^Cterm.OLLAS^* is present in both the visceral and somatic muscles (vm and sm, marked with arrowheads). (H-K′) Endogenously tagged *Kah^Cterm.OLLAS^* in *Alk^10^* mutant background. OLLAS in red (H,I,J,K) or white (H′,I′,J′,K′), Alk in green. (H,H′) Kah^Cterm.OLLAS^ protein is enriched in visceral FCs of controls (arrowheads in H,H′ and J,J′), higher magnification in J,J′. (I,I′) Kah^Cterm.OLLAS^ protein is still detected in the VM of *Alk^10^* mutants (asterisks in I,I′ and K,K′), although the enrichment observed in FCs of control embryos is not observed; higher magnification shown in K,K′. Scale bars: 50 µm.

Our TaDa analysis identified 151 TFs that could be potentially regulated by Alk signaling activity (Fig. 2C,D) (Table S1). We chose to further investigate one of these: the Snail family TF Kahuli (Kah; Fig. 3E). The Snail TF family in *Drosophila* comprises Snail (Sna), Worniu (Wor) and Escargot (Esg), while the Scratch A and B families comprise Scratch (Scrt), CG12605 and Kah, which share a common domain structure with a zinc-finger C2H2-type DNA-binding domain (Fig. 5A,A′). Kah is the only Scratch A family member and lacks the Scratch domain found in Scratch B family members (Kerner et al., 2009). *Kah* mRNA is expressed in a dynamic pattern, initially detected in early embryogenesis (Fig. S6). Expression is seen at stage 10 in the developing VM, and later is enriched in FCs, as revealed by both mRNA *in situ* and our VM-enriched scRNA-seq dataset (Fig. 5B,C). *Kah* expression was also observed in the somatic mesoderm (SM) (Fig. 5C), and in the brain and ventral nerve cord (VNC) (Fig. S6). Our *in situ* data identifying *Kah* expression in both the VM and SM was confirmed in our scRNA-seq datasets (Fig. S5).

The robust *Kah* mRNA signal observed in visceral FCs was consistent with our hypothesis that *Kah* could be a target of Alk activity in the VM. To test this, we assessed *Kah* expression in the VM upon *twist(2xPE)-Gal4*-driven Jeb overexpression, as well as in *jeb* mutants. Ectopic Alk activation led to strong, uniform *Kah* expression throughout the entire VM, whereas loss of Alk signaling in *jeb* mutants reduced, although did not eliminate, *Kah* expression (Fig. 5D, Fig. S6). As expected, *Kah* expression was still robustly expressed in the embryonic SM, as this tissue was unaffected by loss of Alk signaling (Fig. 5D, Fig. S6).

### Kahuli protein is detected in the embryonic VM

**Fig. 6.**
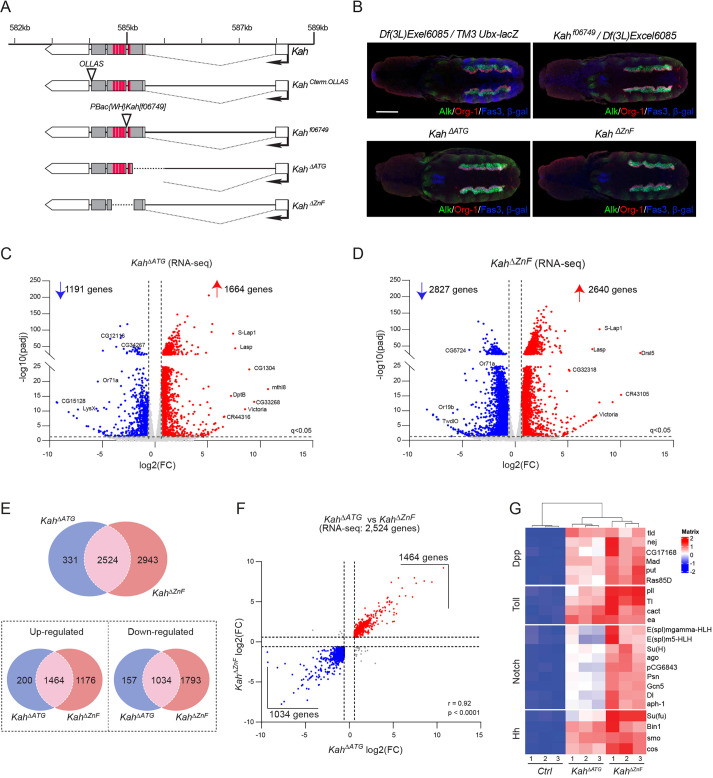
**RNA-seq analysis identifies Kah target genes.** (A) Schematic overview of *Kah* alleles: *Kah^Cterm.OLLAS^*, *Kah^ΔATG^*, *Kah^ΔZnF^* and *Kah^f06749^*. Exon structure is depicted, highlighting protein coding regions (gray) and zinc-finger domains (red). (B) Dorsal views of stage 10–11 control [*Df(3L)Exel6085/TM3,Ubx-lacZ*] and *Kah* mutant [*Kah^f06749^/Df(3L)Exel6085*, *Kah^ΔATG^* and *Kah^ΔZnF^*] embryos stained with Alk (green), the FC-marker Org-1 (red), Fas3 (blue) and β-gal [blue, in control *Df(3L)Exel6085/TM3,Ubx-lacZ*]. (C,D) Volcano plots of differential gene expression measured in RNA-seq from *Kah^ΔATG^* and *Kah^ΔZnF^* mutant embryos. See Table S2 for detailed results. Dashed lines indicate differential gene expression thresholds [FC≥1.5 and ≤−1.5 (log2FC≥0.59 and ≤−0.59)] for up- and downregulated genes respectively (*P*adj≤0.05). Up- or downregulated genes are indicated in red or blue, respectively. A selection of differentially expressed genes are labeled. (E) Venn diagrams indicating the number of differentially expressed genes observed in *Kah^ΔATG^* and *Kah^ΔZnF^* mutants. Top panel, all significantly differentially expressed genes; lower left panel, significantly differentially expressed upregulated genes; lower right panel, significantly differentially expressed downregulated genes. (F) Correlation between the significantly differentially expressed genes (2524) observed in *Kah^ΔATG^* and *Kah^ΔZnf^* mutants. Thresholds used to determine differential expression are indicated by dashed lines [FC≥1.5 and ≤−1.5 (log2FC≥0.59 and ≤−0.59), and *P*adj≤0.05]. Pearson correlation coefficient is indicated in the lower right corner. (G) Heatmap detailing expression of genes in enriched pathways, such as Dpp, Toll, Notch and Hedgehog (Hh) in *Kah^ΔATG^* and *Kah^ΔZnf^* mutants, compared with controls (Ctrl). Color key indicates expression levels. Scale bar: 50 µm.

To visualize Kah protein during embryo development, we employed BAC clone CH322-97G04-derived strain from the modERN (model organism Encyclopedia of Regulatory Networks), which carries an extra copy of the *Kah* locus encoding a C-terminally GFP::FLAG-tagged variant of Kah, under control of endogenous regulatory elements (Kudron et al., 2018). This strain was generated by targeted genomic integration of the *Kah.GFP* recombining BAC into an intronic region of the *Msp300* gene, and does not compromise fly viability (Fig. S7A). Kah-GFP was detected throughout the VM and SM, in agreement with *Kah* mRNA expression (Fig. S8). However, in contrast to our mRNA analyses, Kah-GFP was observed in both VM FCs and FCMs (Fig. S8). Given the large size of GFP and its tendency to form homodimers at high concentrations (Yang et al., 1996), we were concerned this might impact function and stability of the Kah-fusion protein, prompting us to generate a *Kah* allele with a C-terminal 3xOLLAS tag using CRISPR/Cas9-induced homology-directed repair (HDR) (Fig. 5E-G′, Fig. 6A, Fig. S9; referred to as *Kah^Cterm.OLLAS^*). Viable *Kah^Cterm.OLLAS^* animals were obtained that displayed nuclear OLLAS-tag staining in the visceral and somatic muscle (Fig. 5E-G′). *Kah^Cterm.OLLAS^* was enriched in nuclei of VM FCs at stage 11/12, in keeping with our Kah mRNA observations (Fig. 5F,F′,H,H′,J,J′). To investigate whether Alk signaling was crucial for Kah expression, we examined *Kah^Cterm.OLLAS^* in an *Alk^10^* (Lorén et al., 2003) mutant background (Fig. 5H-K′). Kah^Cterm.OLLAS^ was weakly expressed and uniformly distributed in all VM cells of *Alk^10^* mutants at stage 11/12 (Fig. 5I,I′,K,K′), suggesting that Alk signaling is not crucial for the initial expression of Kah, but regulates or maintains Kah expression after visceral FCs are specified.

### Kahuli is required for embryonic midgut constriction

Given the expression of *Kah* in the VM, we next addressed a potential role for Kah in this tissue during embryonic development. We initially characterized a *Kah^f06749^* PiggyBac insertion that was lethal in combination with *Df(3L)Exel6085*, which deletes the entire *Kah* locus [Fig. 6A, Figs S7, S10A; 0% of transheterozygous *Kah^f06749^/Df(3L)Exel6085* survived to L2 larvae (*n*=200)]. To explore Kah function further, we generated two additional alleles using CRISPR/Cas9: (1) *Kah^ΔATG^*, deleting most of exon 2, including the predicted ATG start codon; and (2) *Kah^ΔZnF^*, carrying an in-frame deletion that removes the region encoding the Kah zinc-finger domains (Fig. 6A). Surprisingly, and in contrast to *Kah^f06749^*, *Kah^ΔATG^* and *Kah^ΔZnF^* were viable over *Df(3L)Exel6085* (Fig. S10A). To investigate whether *Kah* mutants exhibit defects in visceral cell fate specification or VM morphology, we visualized Alk, Fas3 and Org-1 at stage 11/12 in *Kah^f06749^/Df(3L)Exel6085*, *Kah^ΔATG^* and *Kah^ΔZnF^* embryos. For all *Kah* alleles, we noted that Alk expression and localization were similar to controls (Fig. 6B). In addition, loss of Kah did not affect early VM cell identity, as indicated by FC-specific expression of Org-1 (Fig. 6B).

To identify a function for Kah in the embryonic VM, we next performed RNA-seq on *Kah* mutants to identify putative targets of this previously uncharacterized TF. RNA-seq was performed on both *Kah^ΔATG^* and *Kah^ΔZnF^* mutants at embryonic stages 11-16. We noted 1664/2640 upregulated genes and 1191/2827 downregulated genes in *Kah^ΔATG^* and *Kah^ΔZnF^* mutants, respectively [threshold values: log2FC≤−0.59 (FC≤−1.5), *P*adj≤0.05] (Fig. 6C,D, Table S2). Further comparison identified 2524 overlapping differentially regulated genes [log2FC≥0.59 and ≤−0.59 (FC≥1.5 and ≤−1.5), *P*adj≤0.05] in both *Kah* mutants (1464 present at increased/1034 at decreased expression levels), the expression of which was correlated significantly (Fig. 6E,F). Many genes identified have yet to be investigated and represent interesting candidates for future functional characterization. As *Kah* encodes a TF, we performed over-representation analysis on these datasets using WebGestaltR (Liao et al., 2019). This led to the identification of BMP (Dpp), Toll, Notch and Hedgehog (Hh) as enriched pathways commonly upregulated in both *Kah^ΔATG^* and *Kah^ΔZnF^* embryos (Fig. 6G, Fig. S11).

**Fig. 7.**
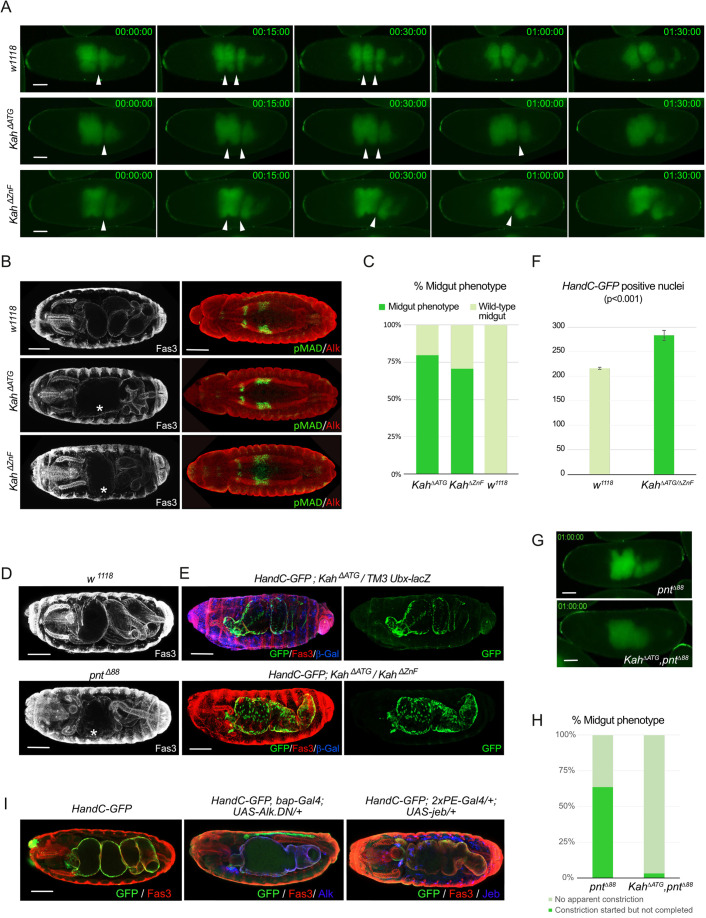
**Kah mutants exhibit defects in midgut constriction.** (A) Live imaging of control (*w^1118^*) embryos at stage 16 identifies three midgut constrictions, while *Kah* mutants (*Kah^ΔATG^* and *Kah^ΔZnF^*) fail to form the first midgut constriction (arrowheads indicate constrictions). Representative frames are shown (see Movies 5-7). (B) Midgut constriction defects in *Kah^ΔATG^* and *Kah^ΔZnF^* are not due to defective Mad signaling. Fas3 (white) highlights midgut structure at stage 16, while anti-pMAD (red) visualizes Mad signaling at stage 13/14; Alk identifies VM (green). Dorsal views. Asterisk indicates midgut constriction phenotype. (C) Quantification of the midgut constriction phenotype observed in *Kah^ΔATG^* (*n*=89) and *Kah^ΔZnF^* (*n*=109) mutants. (D) *pnt^Δ88^* mutants display a midgut constriction phenotype similar to that observed in Kah mutants. Fas3 (white) highlights midgut structure; dorsal views. Asterisk indicates midgut constriction phenotype. (E) *Kah* mutants display abnormal midgut musculature organization, visualized with *HandC-GFP* (green). Lateral views. (F) Quantification of *HandC-GFP*-positive nuclei present in wild-type (*w^1118^*, *n*=30) and *Kah^ΔATG^/Kah^ΔZnF^* (*n*=30) mutants, *P*<0.001. (G) Representative images from live imaging of *pnt^Δ88^* and *Kah^ΔATG^pnt^Δ88^* mutant embryos (see Movies 8 and 9). (H) Quantification of *pnt^Δ88^* (*n*=22) and *Kah^ΔATG^pnt^Δ88^* (*n*=31) mutant midgut constriction phenotypes, indicating the increased severity midgut constriction phenotypes observed in *Kah^ΔATG^pnt^Δ88^* double mutants. (I) Midgut morphology of representative stage 16 *HandC-GFP* control, *HandC-GFP, bap3-Gal4; UAS-Alk.DN/+* and *HandC-GFP; 2xPE-Gal4; UAS-jeb/+* embryos stained for Fas3 (red) and GFP (green). Transgene expression (blue) is revealed by Alk or Jeb antibody staining, as indicated. Scale bars: 50 µm.

As Dpp signaling has been reported to be required for late midgut development (Hursh et al., 1993; Masucci and Hoffmann, 1993), we examined *Kah* mutant midguts [*Kah^f06749^/Df(3L)Exel6085*, *Df(3L)Exel6085*, *Kah^ΔATG^* and *Kah^ΔZnF^*] at later stages using antibody staining and live imaging (Fig. 7A,B, Fig. S10B,C). By stage 16, the midgut of wild-type embryos (Fig. 7A,B,D,E) has acquired three constrictions that subdivide it into four chambers (Campos-Ortega and Hartenstein, 1997; Poulson, 1950; Reuter and Scott, 1990; Schröter et al., 2006). Live imaging of *Kah^ΔATG^* and *Kah^ΔZnF^* mutants identified abnormalities in the first midgut constriction of both mutants (Fig. 7A; Movies 5-7). Using HandC-GFP and Fas3 to visualize the midgut, we found the first midgut constriction was frequently not formed or was incomplete (Fig. 7B,E, quantified in 7C; *Kah^ΔATG^* 80% penetrance, *n*=89; *Kah^ΔZnF^* 70% penetrance, *n*=109; Movies 5-7). This phenotype was also observed in *Kah^f06749^/Df(3L)Exel6085* and in *Df(3L)BSC362/Df(3L)Exel6085* embryos in which *Kah* is entirely deleted (Figs S7B and S10B). As Wingless (Wg) and Dpp signaling events are important for midgut constriction, and our RNA-Seq analysis identified differential gene expression in Dpp signaling components in *Kah* mutants, we investigated Wg, Dpp and pMAD expression in *Kah* mutants. Wg protein, Dpp mRNA and robust pMAD expression was observed (Fig. 7B, Fig. S10C), suggesting that the defective midgut constriction observed in the absence of Kah is not due to loss of Wg or Dpp signaling. Although no decrease in pMAD was observed, we cannot exclude increased signaling through this pathway, which would reflect the increased expression of pathway components in our *Kah* mutant RNA-seq datasets and that may result in disruption of the midgut constriction process.

We next turned towards TFs with reported VM constriction phenotypes, such as Org-1 and Pointed (Pnt), as well as Hand and H2O, which exhibit FC-specific expression but have no obvious phenotypes in the embryonic VM (Barad et al., 1991; Bilder et al., 1998; Lo et al., 2007; Schaub and Frasch, 2013). Interestingly, a physical interaction of the ETS TF Pnt with Kah has been reported by Y2H (Thurmond et al., 2019), http://flybi.hms.harvard.edu/results.php, prompting us to further investigate similarities between *pnt* and *Kah* mutants. Analysis of the amorphic *pnt^Δ88^* allele confirmed the previously described midgut constriction phenotype (Fig. 7D) (Bilder et al., 1998). Using the *HandC-GFP* reporter, we revealed that *Kah* mutant embryos have an increased number of visceral nuclei in the midgut (Fig. 7E; quantified in 7F, *n*=30, *P*<0.001). These HandC-GFP-positive nuclei were also highly disorganized when compared with controls where visceral muscle nuclei are aligned in four rows (Fig. 7E). We next performed epistasis experiments between our *Kah* loss-of-function alleles and *pnt^Δ88^*, identifying incomplete anterior midgut constriction formation in ∼12% of late stage transheterozygous embryos (*n*=154). Live imaging of *Kah^ΔATG^*, *pnt^Δ88^* as well as *Kah^ΔATG^,pnt^Δ88^* double mutant embryos revealed that the *pnt^Δ88^* mutant constriction phenotype increased in severity in *Kah^ΔATG^, pnt^Δ88^* double mutants, which completely failed to initiate anterior constriction (Movies 8, 9, representative images in Fig. 7G; quantified in 7H). Taken together, these findings suggest that Kah and Pnt may function together to accomplish the anterior midgut constriction. Finally, we asked whether Alk signaling could impact on the midgut constriction process. To do this, we expressed dominant-negative Alk (*bap-Gal4>UAS-Alk.DN*) in the VM, observing defects in midgut constriction at low penetrance (34%, *n*=190) (Fig. 7I). Interestingly, we also observed a range of late gut phenotypes upon pan-mesodermal overexpression of Jeb (*2xPE-Gal4>UAS-jeb*), ranging from absent or incomplete midgut constriction with occasional bulging of the visceral muscle layer (56%, *n*=37) to severe defects that affected overall midgut formation (44%, *n*=37) (Fig. 7I). Thus, Alk signaling appears to be important for later events in midgut development, particularly midgut constriction formation.

### Analysis of Kah and Pnt ChIP-seq datasets identifies common targets and a Kah-binding motif

**Fig. 8.**
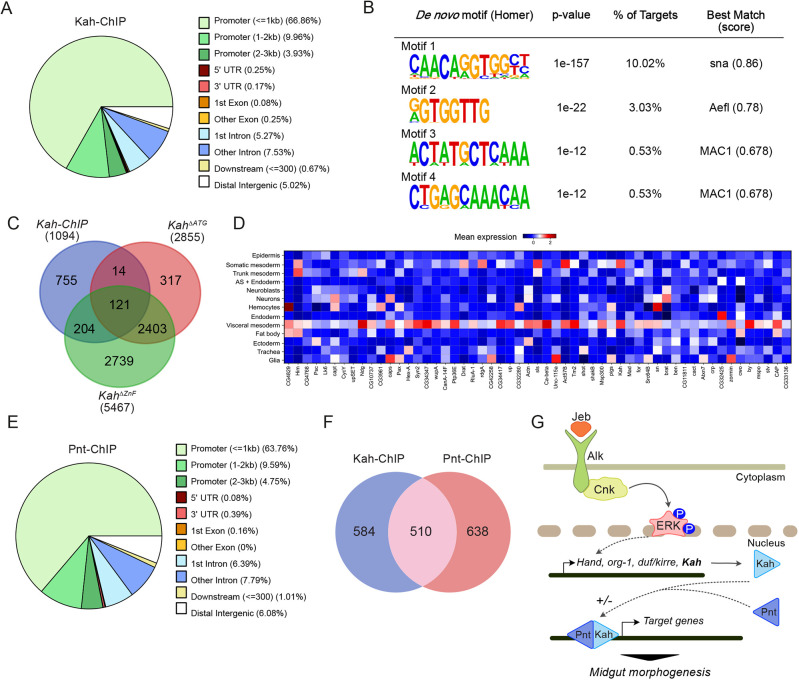
**ChIP analysis identifies a Kah putative binding site and putative common targets of Kah and Pnt.** (A) Genomic location distribution in the Kah-ChIP dataset. Pie chart indicating different genomic regions statistically enriched in Kah-ChIP relative to promoter, UTR, intron/exon and other regions (see key). Promoter regions (≤1 kb) are heavily represented. Data were extracted from Roy et al. (2010). (B) Analysis of motif enrichments in regions of 50 bp around the peak center identifies a putative Kah-binding motif highly related to the Sna-binding motif. (C) Venn diagram showing the overlap between Kah-ChIP and *Kah* mutant RNA-seq datasets (details in Table S2). (D) Matrix-plot visualizing expression levels for genes common to Kah-ChIP, Pnt-ChIP and *Kah* mutant RNA-seq datasets that were represented in the whole embryo scRNA-seq dataset. (E) Pie-chart indicating Pnt ChIP-seq peak locations in the genome, relative to promoter, UTR, intron/exon and other regions (key at right). Promoter regions (≤1 kb) are heavily represented. Data extracted from Roy et al. (2010). (F) Venn diagram showing the proportion of overlapping genes between Kah- and Pnt-ChIP datasets. Details in Table S2. (G) Model for Alk-mediated regulation of embryonic VM development involving Kah and Pnt. Alk activation in the VM is driven by Jeb binding, which induces signaling and activates the transcription of FC-specific genes, including *Hand*, *org-1*, *duf/kirre* and *Kah*. Kah may work in concert with other transcriptional regulators, such as Pnt, to target genes involved in the formation of the first midgut constriction.

To better understand Kah function, we employed ChIP-seq data from *Kah-eGFP* embryos that has been deposited publicly by the modENCODE project (accession number ENCSR161YRO) (Roy et al., 2010). This *Kah-eGFP* ChIP dataset contains a predominance of promoter regions with a peak in the vicinity of transcription start sites (TSS) (Fig. 8A, Fig. S12A,B). A Basic Motif search in regions 50 bp and 200 bp around the peaks led to the generation of a *de novo* motif for Kah with highest scoring similarity to the related Snail TF (Fig. 8B), in agreement with a recent study (Reddington et al., 2020). Among those genes containing a Kah motif in the vicinity of the TSS, we noted a number expressed in the visceral mesoderm, such as *Antennapedia* (*Antp*), *mind bomb 2* (*mib2*) and *Netrin-B* (*NetB*) (Table S3). Comparison of differentially expressed genes identified in *Kah* mutants by RNA-seq with the Kah ChIP-seq dataset showed that 31% (339/1094) of genes in the Kah ChIP-seq dataset were differentially regulated in *Kah* mutants (Fig. 8C, Table S3). Further interrogation of our whole-embryo scRNA-seq dataset (Fig. 4) showed that these genes were more highly expressed across the visceral mesoderm cluster (Fig. 8D).

ChIP-seq data from transgenic *Pnt-eGFP* embryos has also been deposited publicly by the modENCODE project (Fig. 8C, Fig. S12C) (accession number ENCSR997UIM) (Roy et al., 2010), allowing us to compare Pnt and Kah binding locations throughout the genome, together with genes differentially expressed in Kah mutants. This analysis revealed that 46% (510/1094) of Kah-ChIP targets are potentially occupied by both Kah and Pnt (Table S3). Furthermore, 30% (157/510) of these common ChIP targets overlapped with genes differentially expressed in *Kah* mutants (Table S3). These overlapping genes include *Antp*, which is known to play an important role in setting up the first midgut constriction (Bilder et al., 1998; Roy et al., 1997), as well as *Kah* itself. We also found *nej*, *put*, *Mad* and *Ras85D*, which were upregulated in both RNA-seq datasets of *Kah^ΔATG^* and *Kah^ΔZnF^* mutants (Fig. 6G) as common Kah- and Pnt-ChIP targets. Taken together, our ChIP analysis together with our *Kah* mutant RNA-seq datasets identified a set of genes that are potentially regulated by Kah and Pnt downstream of Alk signaling during midgut constriction, some of which likely play important roles in this process (Fig. 8G).

## DISCUSSION

### Alk targets in the VM

Specification of FCs in the VM depends on Alk signaling in response to Jeb secretion from the somatic mesoderm (Englund et al., 2003; Lee et al., 2003; Stute et al., 2004). Signaling via Alk activates the Ras/MAPK pathway, translocating the FCM fate-promoting TF Lameduck (Lmd) from the nucleus to the cytoplasm (Popichenko et al., 2013). A similar mechanism has been suggested for a still unknown FC-fate repressor triggering the FC-specific transcriptional program in the VM (Popichenko et al., 2013; Zhou et al., 2019). This transcriptional program remains relatively unexplored with only a few identified targets reported, such as *Hand*, *org-1*, *kirre*, *dpp* and *Alk* itself (Englund et al., 2003; Lee et al., 2003; Mendoza-García et al., 2017; Shirinian et al., 2007; Varshney and Palmer, 2006). Although ChIP has been the predominant approach for mapping protein-chromatin interactions, it requires significant amounts of starting material and specific antibodies (Wu et al., 2016). RNA-seq has also been intensely employed for transcriptomic analyses and, although straightforward for cell culture studies, isolation of the VM would be required for its use in identifying Alk transcriptional targets in *Drosophila*. Therefore, in our efforts to identify novel Alk transcriptional targets in the VM, we employed TaDa, which allows genome-wide RNA PolII occupancy to be investigated in the specific tissue of choice (Southall et al., 2013). TaDa requires less starting material and provides cell-type specificity, although resolution can be less accurate compared with RNA-seq and ChIP-seq due to its dependency on frequency of GATC sites in the genome (Marshall et al., 2016).

### TaDa reproduces endogenous RNA Pol II occupancy

Our experimental design was based on either activating or inhibiting Alk signaling throughout the VM, followed by TaDa analysis. Comparison of our TaDa dataset with previously published RNA-seq data (NCBI BioProject, PRJEB11879) from cells isolated from the mesoderm suggest our data recapitulated endogenous binding of RNA PolII. Our dataset also agreed with current understanding of Alk signaling and induction of cell fate specification in the trunk VM (Lee et al., 2003; Lorén et al., 2003; Stute et al., 2004), including observed differential expression of previously identified Alk transcriptional targets, such as *Hand*, *org-1*, *kirre* and *dpp*. However, these FC-enriched targets were not the most significantly expressed genes within our TaDa dataset, perhaps reflecting lower levels or smaller temporal windows of active transcription that may additionally be complicated by differentially stable mRNA or protein products. Taken together, a combination of different analyses supported our approach as replicating transcriptional events in the VM and led us to validate of TaDa-identified genes in the VM as targets of Alk-driven signaling events.

### TaDa identified VM-specific genes

A number of differentially expressed genes were validated by *in situ* hybridization during embryogenesis. mRNA was indeed visualized in the visceral musculature, and VM expression was confirmed in our *HandC-GFP* scRNA-seq dataset for *fax*, *Sumo* and *CG11658*. Expression of *fax* was observed in the VM and CNS, as previously reported (Hill et al., 1995). Interestingly, Fax has been identified in a screen for diet-regulated proteins in the *Drosophila* ovary. Insulin signaling in response to diet promotes activation of the Ribosomal protein S6 Kinase (S6K), which drives *fax* expression (Hsu and Drummond-Barbosa, 2017; Su et al., 2018). Notably, Alk modulates insulin signaling in the brain during nutrient restriction (Cheng et al., 2011; Okamoto and Nishimura, 2015), making Fax interesting for further study. Another interesting candidate is *Sumo*, which encodes the *Drosophila* SUMO-1 homologue (Abed et al., 2018). Sumo is not exclusively expressed in the VM, but is rather expressed maternally and ubiquitously throughout the embryo (Shigenobu et al., 2006). Functional studies have identified a role for Sumo in the post-translational modification of several TFs, as well as in modulation of signaling in the fly (Huang et al., 2011; Sánchez et al., 2010). Overall, our TaDa analysis identified numerous uncharacterized genes, and further investigation will be crucial to decipher their role in the developing visceral muscles.

### *Kahuli* plays a role in later visceral musculature development

One interesting uncharacterized target was *Kah*, which encodes a Snail family TF. Kah overexpression in the thorax has been reported to block development of thoracic bristles, revealing a potential to drive changes in cell identity (Singari et al., 2014). We were able to validate *Kah* as an Alk target locus in the embryonic VM, with differences in *Kah* expression when Alk signaling was either blocked or activated. However, we also noted Alk-independent *Kah* transcription in the early VM in addition to the Alk-modulated transcription that is reminiscent of the VM expression reported for *org-1* (Schaub and Frasch, 2013). Currently, the TFs downstream of Alk that regulate *Kah* transcription are unknown, although this will be interesting to study in the future.

Alk signaling in the VM drives FC specification via Ras/MAPK pathway activation, leading to the transcription of FC-specific genes such as *Hand*, *org-1*, *kirre*, *dpp* and also *Kah* (Englund et al., 2003; Lee et al., 2003; Shirinian et al., 2007; Stute et al., 2004; Varshney and Palmer, 2006). Loss of *Hand*, *org-1* and *dpp* does not alter FC specification in the VM, suggesting a complex temporal regulation that assures FC specification and eventually formation of visceral muscles (Hursh et al., 1993; Masucci and Hoffmann, 1993; Schaub and Frasch, 2013; Varshney and Palmer, 2006). Our characterization of *Kah* mutant alleles suggest that, similar to *Hand* and *org-1*, *Kah* is dispensable for VM FC specification, although formally Kah could be responsible for FC-specific transcriptional changes of yet unidentified targets.

*Kah* mutants exhibit defects in midgut constriction formation. A number of players are implicated in this event, such as Wg, Dpp, Ultrabithorax (Ubx), Pnt, Extra macrochaetae (Emc) and Org-1 (Bilder et al., 1998; Ellis et al., 1990; Müller et al., 1989; Panganiban et al., 1990; Reuter et al., 1990; Schaub and Frasch, 2013). Interestingly, Alk signaling activity is important for VM Dpp expression and maintenance of Org-1 in FCs (Popichenko et al., 2013; Shirinian et al., 2007). Although it is difficult to define a role for Alk in VM events post FC specification, we show that Alk may indeed play a role in later midgut development. We also noted that inappropriate activation of Alk signaling via Jeb expression results in a range of later gut defects. Thus, perturbation of Alk signaling appears to disrupt later events in midgut constriction. However, whether this is a consequence of earlier, Alk-dependent, visceral FC specification or refers to an independent role of Alk signaling in the VM is unclear. We were particularly interested in Pnt, as this ETS domain TF has been reported by the FlyBi-project (http://flybi.hms.harvard.edu/) to bind Kah in high-throughput Y2H (Thurmond et al., 2019), and *pnt* mutant embryos also exhibit a midgut constriction phenotype (Bilder et al., 1998). Like Kah, Pnt is not required for VM FC specification (Zhou et al., 2019). The observation of low penetrance midgut constriction defects in transheterozygous *Kah^ΔATG^*/*pnt^Δ88^* embryos suggest that Kah and Pnt may function together in this process, which is further supported by live imaging, through which we observed stronger midgut constriction phenotypes in *Kah, pnt^Δ88^* double mutants than in *pnt^Δ88^* alone. Employing publicly available Kah-ChIP datasets (Roy et al., 2010), we could define a Kah-binding motif similar to that of Snail. Analysis of publicly available Pnt-ChIP datasets (Roy et al., 2010) identified numerous targets of both Kah and Pnt, including *Kah* itself, *nej*, *put*, *Mad* and *Ras85D*. Interestingly, although several components of the Dpp signaling appear to be misregulated in *Kah* mutants, *Wg* and *dpp* expression appear normal and robust pMAD activity is observed in both *Kah^ΔATG^* and *Kah^ΔZnF^* mutants. Earlier work reported that *Wg* and *dpp* expression are also normal in the VM of *pnt* mutant embryos (Bilder et al., 1998). It is likely that as yet unidentified players function downstream of Kah in this process, and we have not yet been able to identify a key component downstream of Kah that could explain the underlying molecular mechanisms. It is also important to note that, although in this work we have focused on a role in the VM, Kah is also expressed in the embryonic SM and CNS. Our analysis of *Kah* mutant RNA-seq together with Kah-ChIP and Pnt-ChIP datasets identifies candidates to be focused on in future studies.

### Conclusions

The TaDa approach successfully allowed us to identify transcriptional targets of Alk signaling in the developing mesoderm, including the transcriptional regulator Kahuli described here. Many of these targets are currently uncharacterized and future studies should allow their function(s) in the VM to be elucidated. Our in-depth study of Kah highlights a role for this TF in later visceral musculature development, where it appears to work in concert with other factors, including Pnt, to regulate midgut constriction. Combined ChIP and RNA-seq analyses highlights a group of interesting and largely uncharacterized genes, which should shed further light on the midgut constriction process.

## MATERIALS AND METHODS

### *Drosophila* stocks and genetics

LacZ or GFP balancer chromosomes were used to distinguish progeny of crosses. The following stock lines were obtained from the Bloomington *Drosophila* Stock Center (BDSC): *TM3 Sb Ubx-lacZ* (#9120), *Df(3L)BSC362* (*Kah* deficiency, #24386), *Df(3L)Exel8065* (*Kah* deficiency, #7564), *Kah^f06749^* (PiggyBac insertion mapped to the second intron of *Kah*, #19006), *Kah-GFP.FPTB* (#64829), *twi.2xPE-Gal4* (#2517) and *Df(2R)BSC199* (*jeb* deficiency, #9626). Additional stocks used in this study were: *UAS-LT3-NDam-Pol II* (Southall et al., 2013), *rP298-lacZ* (Nose et al., 1998), which is an enhancer trap in the *kirre* locus (Ruiz-Gómez et al., 2000), *HandC-GFP* (Sellin et al., 2006), *bap3-Gal4* (Zaffran et al., 2001), *UAS-jeb.V* (Varshney and Palmer, 2006), *UAS-Alk.EC.MYC* (Bazigou et al., 2007) (Alk extracellular domain that functions as dominant negative, here referred to as *UAS-Alk.DN*), jebweli (Stute et al., 2004) and *Alk^10^* (Lorén et al., 2003).

### TaDa sample preparation

*Twi.2xPE-Gal4* and *bap3-Gal4* lines were used to test Dam-Pol II toxicity and further RNA-Pol II profiling. Embryos were collected over a 4 h period and aged at 25°C to stages 10-13, followed by dechorionation in 2% sodium hypochlorite solution for 2 min and subsequent washing steps in PBS. A total of 50 µl embryos per sample was used as starting material. Genomic DNA was extracted (QIAGEN DNeasy Blood and Tissue kit, #69504) and methylated DNA was processed and amplified as previously described (Choksi et al., 2006; Sun et al., 2003), with the following modifications. Upon verification of non-sheared gDNA, the DpnI digestion was set up in 50 µl. After overnight DpnI digestion, the DNA was purified (QIAGEN MinElute PCR purification kit, #28004) into 30 µl of deionized water, from which 15 µl were used for the adaptors-ligation step. Amplified DNA from experimental and Dam-only embryos was again purified (QIAGEN MinElute PCR purification kit) into 20 µl of deionized water and 200 ng aliquots were run in a 1% agarose gel to verify amplification of different fragments (visualized as a smear from 500 bp to 2-3 kb). Purified PCR products were used for PCR-free library preparation, followed by pair-end sequencing on Illumina HiSeq X Ten platform (BGI Tech Solutions, Hong Kong). Three biological replicates were performed for transcriptional profiling of the visceral mesoderm on each of the experimental genetic backgrounds.

### DamID-seq bioinformatics data analysis

The *Drosophila* genome (FASTA) and genes (GTF) version r6.21 were downloaded from FlyBase (Gramates et al., 2017; Thurmond et al., 2019) and all GATC regions extracted in BED format using fuzznuc (Rice et al., 2000). The paired FASTQ files from 18 samples (background Dam, Jeb and DN with three replicates each for *twi.2xPE-Gal4* and *bap3-Gal4*) were aligned to the *Drosophila* genome using *bowtie2* (*--very-sensitive-local*) (Langmead and Salzberg, 2012). *Sambamba* (merge) (Tarasov et al., 2015) was used to combine replicates and the log-fold changes between DN/Jeb and Dam, obtained using *bamCompare* (*--centerReads --normalizeTo1x 142573017 --smoothLength 5 -bs 1*) from deepTools (Ramírez et al., 2014). Counts of reads mapped on edge to GATC fragments were generated using a script (GATC_mapper.pl) from DamID-Seq pipeline (Maksimov et al., 2016). RNA-seq data from a public dataset (PRJEB11879) was used to quantify the expression of genes (only 6-8 h mesoderm samples used). The GATC level counts were converted to gene level counts using intersectBed from Bedtools (Quinlan, 2014) and compared against the gene expression (only background Dam samples) at TPM level. GATC sites were merged into peaks following the methods prescribed in a previous study (Tosti et al., 2018). In brief, logFC for individual GATCs were generated using Limma (Jeb versus Dam and DN versus Dam) (*P*<1e-5) and the GATC sites were merged into peaks based on median GATC fragment distance in the *Drosophila* genome using *mergeWindows* and *combineTests* function from the *csaw* package (Lun and Smyth, 2016). The peaks were assigned to overlapping genes and filtered for FDR at 0.01. The final results were taken only for the DN versus Dam comparison. The Jeb samples had low rate of alignment, hence Jeb versus Dam is only used as a visual confirmation of the DN versus Dam peaks at specific locations. Enrichment of TFs in the peaks generated was performed by using Fisher test against list of published *Drosophila* TFs (Kudron et al., 2018). Enrichment for GO and KEGG terms for the genes assigned to significant peaks was performed using *WebGestalt* (Liao et al., 2019). All statistical analysis was performed in the R programming environment.

### Embryo dissociation into single cells and cell sorting

Embryos were collected on apple juice agar plates and aged to stages 10-13 (as confirmed by microscopic visualization of a small fraction). Embryos were dechorionated in 2% sodium hypochlorite solution for 2 min and washed in ice-cold PBS. Subsequently, embryos were incubated in dissociating solution (1 mg/ml trypsin, 0.5 collagenase I, 2% BSA) for 1 h and vortexed every 10 min, after which the reaction was stopped by addition of 10 volumes of ice-cold PBS. Dissociated cell solution was sieved through 70 µm and 40 µm cell strainers to remove cell clumps. Dead cells or debris from the dissociated samples were removed using the EasySep Dead Cell Removal (Anexin V) Kit (STEMCELL, 17899) according to the manufacturer's guidelines. The remaining cells were respectively labelled with aqua-fluorescent reactive dye (dying cells) and calcein violet AM (living cells) using the LIVE/DEAD Violet Viability/Vitality Kit (Molecular Probes, L34958) under manufacturer's guidelines. Finally, each sample was washed twice in PBS/2% fetal bovine serum and resuspended in 500 µl PBS/2% fetal bovine serum. Living cells were enriched using a FACSAria III cell sorter (BD biosciences) based on the LIVE/DEAD staining and, when appropriate, GFP expression driven by the *HandC-GFP* construct (Sellin et al., 2006). The cells were sorted using an 85 µm nozzle into Eppendorf tubes that had been pre-coated with PBS/2% BSA.

### Generation of single cell libraries, sequencing and bioinformatic analysis

Approximately 2500 sorted cells were directly loaded in sheath fluid onto one lane of a Chromium 10X chip (10X Genomics) and libraries prepared using the normal workflow for Single Cell 3´ v3 libraries (10X Genomics). Libraries were sequenced on the NextSeq 500 platform (Illumina), and the raw format base call file (BCL) sequences were demultiplexed using cellranger mkfastq version 3.1. After read QC, mapping was performed with the *Drosophila melanogaster* genome using STAR aligner. For analysis, unique molecular identified (UMI) count matrix were imported into the Seurat R toolkit version 3.1. For quality filtering, cells with fewer than 1000 genes and more than 5000 expressed genes were excluded. In addition, cells that expressed more than 25% mitochondrial genes were removed. Subsequent count normalization, scaling, feature selection, clustering (PCA) and dimensionality reduction (UMAP, Uniform Manifold Approximation and Projection) were performed using Seurat (Stuart et al., 2019) and Scanpy (Wolf et al., 2018). After preprocessing, 1055 and 888 cells with a total of 11,029 and 10,602 RNA features remained, for the whole embryo and *HandC-GFP* scRNA-seq datasets, respectively.

To identify relationships and correlations between clusters, hierarchical clustering and correlation matrices were calculated using Pearson correlation (Scanpy). Based on canonical markers and previously known markers, the cellular heterogeneity of the whole embryo with 13 cell types [epidermis, somatic mesoderm, early trunk mesoderm, amnioserosa (AS) and endoderm, neuroblasts, neurons, hemocytes, endoderm, visceral mesoderm, fat body, ectoderm, trachea and glia] and the *HandC-GFP* scRNA-seq with five clusters (VM founder cells, early visceral muscle, cardiac mesoderm, VM proliferating cells and late visceral muscle) were determined. Clusters were identified using the Louvain algorithm (Blondel et al., 2008) and visualized by UMAP projection. Feature plots, violin plots, matrix plots, heatmaps and dot plots were plotted for visualizing the marker genes expression and percentage of cell distribution. Gene Ontology (GO) term enrichment was performed on genes commonly upregulated in both *Kah^ΔATG^* and *Kah^ΔZnF^* mutants using WebGestalt (PANTHER functional database) (Liao et al., 2019). Markers employed for determination of VM proliferating cells were defined by gene ontology analysis using g:Profiler (Biological Process) (Raudvere et al., 2019).

### CRISPR/Cas9-mediated generation of mutant and tagged *Kah* alleles

Deletion (*Kah^ΔATG^* and *Kah^ΔZnF^*) and endogenous tagged (*Kah^Cterm.OLLAS^*) *Kah* alleles were generated using the CRISPR/Cas9 system. CRISPR target sites were identified and evaluated using flyCRISPR Optimal Target Finder tool (Gratz et al., 2015). Single guide RNA (sgRNA) target sequences (sequences available in Table S4) were cloned into pU6-BbsI-chiRNA vector (Addgene plasmid #45946; Gratz et al., 2013) and injected into *vasa-Cas9* (BDSC, #51323) embryos (BestGene). For *Kah^Cterm.OLLAS^* a donor construct was added to the injection mix. Injected animals were crossed to third chromosome balancer flies and resulting progeny PCR screened for positive deletion/insertion events. Positive candidates were confirmed further by Sanger sequencing (Eurofins Genomics).

Endogenously tagged *Kah^Cterm.OLLAS^* was generated using CRISPR/Cas9-induced homology-directed repair (HDR) at the Kah C-terminal. Three CRISPR sgRNA sequences (sequences available in Table S4) were used. One sgRNA was designed to target upstream to the *Kah* stop codon and the other two to target directly the *Kah* stop codon. In addition, a DNA donor cassette was synthesized (Integrated DNA Technologies) before Gibson assembly cloning into the pBluescript II KS[-] HDR plasmid. This donor cassette codes for the remaining part of the Kah C-terminal followed by six glycines (linker), three copies of the OLLAS-tag and a TAG stop codon; flanked by two homology arms (495 bp upstream and 500 bp downstream of the respective Cas9 cutting sites, with codon optimized target sequences).

### ChIP bioinformatics-based determination of Kah- and Pnt-binding motifs

Publicly available *dm6* aligned Kah ChIP-seq data input libraries (ENCSR161YRO and ENCSR664RUV) and the raw format FASTQ sequences of Pnt ChIP-seq (ENCSR997UIM and ENCSR249WKC) were retrieved from modEncodeID. For Pnt ChIP-seq data, the base quality of each sequenced read was assessed using the FASTQC program. The reads were aligned to the *Drosophila melanogaster* (BDGP6) reference genome using Bowtie2. Owing to the ambiguity of reads that align to multiple locations across the genome, only reads that uniquely mapped were considered for subsequent analysis. Post-alignment processes were performed with samtools and BEDtools, and Homer suite v4.1 program ‘findpeaks’ (Heinz et al., 2010) with default TF finding parameters (-style factor) used for peak calling. Resulting peaks from each replicate were annotated using ChIPseeker v1.2 (Yu et al., 2015) and merged to be used as input for genome-wide motif enrichment scanning using the ‘findMotifsGenome.pl’ script from the Homer suite. Regions of 50 bp and 200 bp around the peak center were analyzed for motif enrichment.

### RNA-sequencing and analysis

Embryos were collected at 25°C (5-16 h after egg laying) and dechorionated in 2% sodium hypochlorite solution for 4 min. After subsequent washing with embryo wash (0.8% NaCl and 0.05% Triton X-100) and H_2_O, embryos were stored at −80°C. One embryo collection for three biological replicates per genotype was obtained. RNA-extraction was carried out according to the manufacturer's protocol (Promega ReliaPrepTM RNA Tissue Miniprep System, REF-Z6111). Total RNA was measured using NanoDrop OneC (Thermo Scientific) for its concentration and RNA integrity was checked using gel electrophoresis. Then 9-15 µg of total RNA/biological replicate was shipped to Novogene for sequencing. Prior to making the library, samples were reassessed for quality with the Agilent 2100 Bioanalyzer system. Sequencing was performed on an Illumina platform and paired-end reads were produced. Over 40 million reads/genotype were generated and mapped to the genome at a rate of over 96%. *Drosophila melanogaster* (ensemble bdgp6_gca_000001215_4 genome assembly) was used. HISAT2 algorithm for alignment and DESeq2 R package (Anders and Huber, 2010) for differential gene expression was used. Subsequent analyses were performed with GraphPad Prism 9. Fold change ≥1.5 and ≤−1.5 (log2FC≥0.59 and ≤−0.59) for up- and downregulated genes, respectively, and *P*_adj_≤0.05 were used for statistical significance.

### Immunohistochemistry

Embryos were fixated and stained as described previously (Müller, 2008). Primary antibodies used were: guinea pig anti-Alk (1:1000; Lorén et al., 2003), guinea pig anti-Jeb (1:1000; Englund et al., 2003), rabbit anti-Alk (1:750; Lorén et al., 2003), chicken anti-β-galactosidase (1:200; Abcam ab9361), mouse anti-Fasciclin3 (1:50; DSHB 7G10), rabbit anti-GFP (1:500; Abcam ab290), chicken anti-GFP (1:300; Abcam ab13970), mouse anti-Wg (1:50; DSHB 4D4), rat anti-OLLAS (1:200, pre-absorbed on w1118 embryos; Abnova), rabbit anti-Org-1 (1:1000; Mendoza-García et al., 2017), sheep anti-digoxygenin-AP fab fragment (1:4000, Roche), rabbit anti-phospho-Smad1/5 (41D10) (1:500; Cell Signaling Technologies 9516). Alexa Fluor-conjugated secondary antibodies were from Jackson Immuno Research. Embryos were dehydrated in an ascending ethanol series before clearing and mounting in methylsalicylate. Images were acquired with a Zeiss LSM800 confocal microscope or Axiocam 503 camera, processed and analyzed employing Zeiss ZEN2 (Blue Edition) imaging software. *HandC-GFP* positive VM nuclei quantification (Fig. 7F) was performed in triplicate for each genotype (*n*=10 per replicate, in total *n*=30 per genotype) with ImageJ software (Schneider et al., 2012). Raw images were converted into binary format and nuclei were quantified using 3D nuclei counter package (Bolte and Cordelieres, 2006). To identify statistical differences between *Kah* mutants and controls (*w^1118^*), *t*-test analysis was performed.

### Live embryo imaging

Overnight collection of embryos was performed at 25°C followed by dechorionation in 2% sodium hypochlorite solution for 4 min. After subsequent washing with embryo wash solution (0.8% NaCl and 0.05% Triton X-100) and H_2_O, stage 14-early 16 embryos were sorted manually with a stereo/epi-fluorescence microscope. Afterwards embryos were transferred into 96-well plates and live imaged with a Zeiss Cell Discoverer 7 at 1 min intervals with time series setting. Further processing was performed with FIJI ImageJ. For quantification of the midgut phenotype of *pnt^Δ88^* and *Kah^ΔATG^pnt^Δ88^* double mutant embryos, manual scoring of live imaged embryos (stage 15 onward) was performed. Scoring was based on two criteria: (1) 1st midgut constriction started but not completed; and (2) no apparent constriction. Results are shown as a percentage of embryos scored.

### *In situ* hybridization

For *in situ* hybridization, fragments of the respective CDS were PCR amplified from genomic DNA, cloned into the dual promoter pCRII-TA vector (ThermoFisher, K207040) and used as a template to generate DIG-labeled *in situ* probes with SP6/T7 polymerases (Roche, 10999644001). Whole-mount *in situ* hybridization was carried out according to Lécuyer et al. (2008), with modifications adapted from Pfeifer et al. (2012). Samples were mounted using *in situ* mounting media (Electron Microscopy Sciences). Images were acquired with a Zeiss Axio Imager.Z2 microscope, processed and analyzed using Zeiss ZEN2 (Blue Edition) imaging software.

## Supplementary Material

Supplementary information

Reviewer comments

## References

[DEV199465C1] Abed, M., Bitman-Lotan, E. and Orian, A. (2018). The biology of SUMO-targeted ubiquitin ligases in Drosophila development, immunity, and cancer. *J. Dev. Biol.* 6, 2. 10.3390/jdb6010002 PMC5875560 29615551

[DEV199465C2] Anders, S. and Huber, W. (2010). Differential expression analysis for sequence count data. *Genome Biol.* 11, R106. 10.1186/gb-2010-11-10-r106 20979621 PMC3218662

[DEV199465C3] Aughey, G. N. and Southall, T. D. (2016). Dam it's good! DamID profiling of protein-DNA interactions. *Wiley Interdiscip. Rev. Dev. Biol.* 5, 25-37. 10.1002/wdev.205 26383089 PMC4737221

[DEV199465C4] Barad, M., Erlebacher, A. and McGinnis, W. (1991). Despite expression in embryonic visceral mesoderm, H2.0 is not essential for Drosophila visceral muscle morphogenesis. *Dev. Genet.* 12, 206-211. 10.1002/dvg.1020120305 1678322

[DEV199465C5] Bazigou, E., Apitz, H., Johansson, J., Lorén, C. E., Hirst, E. M. A., Chen, P.-L., Palmer, R. H. and Salecker, I. (2007). Anterograde Jelly belly and Alk receptor tyrosine kinase signaling mediates retinal axon targeting in Drosophila. *Cell* 128, 961-975. 10.1016/j.cell.2007.02.024 17350579

[DEV199465C6] Bilder, D., Graba, Y. and Scott, M. P. (1998). Wnt and TGFbeta signals subdivide the AbdA Hox domain during Drosophila mesoderm patterning. *Development* 125, 1781-1790. 10.1242/dev.125.9.1781 9521915

[DEV199465C7] Blondel, V. D., Guillaume, J.-L., Lambiotte, R. and Lefebvre, E. (2008). Fast unfolding of communities in large networks. *J Stat Mech-Theory E*. 2008. 10.1088/1742-5468/2008/10/P10008

[DEV199465C8] Bolte, S. and Cordelieres, F. P. (2006). A guided tour into subcellular colocalization analysis in light microscopy. *J. Microsc.* 224, 213-232. 10.1111/j.1365-2818.2006.01706.x 17210054

[DEV199465C9] Brand, A. H. and Perrimon, N. (1993). Targeted gene expression as a means of altering cell fates and generating dominant phenotypes. *Development* 118, 401-415. 10.1242/dev.118.2.401 8223268

[DEV199465C10] Campos-Ortega, J. A. and Hartenstein, V. (1997). *The Embryonic Development of Drosophila melanogaster*. Berlin, Germany: Springer.

[DEV199465C11] Cheng, L. Y., Bailey, A. P., Leevers, S. J., Ragan, T. J., Driscoll, P. C. and Gould, A. P. (2011). Anaplastic lymphoma kinase spares organ growth during nutrient restriction in Drosophila. *Cell* 146, 435-447. 10.1016/j.cell.2011.06.040 21816278

[DEV199465C12] Choksi, S. P., Southall, T. D., Bossing, T., Edoff, K., de Wit, E., Fischer, B. E., van Steensel, B., Micklem, G. and Brand, A. H. (2006). Prospero acts as a binary switch between self-renewal and differentiation in Drosophila neural stem cells. *Dev. Cell* 11, 775-789. 10.1016/j.devcel.2006.09.015 17141154

[DEV199465C13] Ellis, H. M., Spann, D. R. and Posakony, J. W. (1990). extramacrochaetae, a negative regulator of sensory organ development in Drosophila, defines a new class of helix-loop-helix proteins. *Cell* 61, 27-38. 10.1016/0092-8674(90)90212-W 1690604

[DEV199465C14] Englund, C., Lorén, C. E., Grabbe, C., Varshney, G. K., Deleuil, F., Hallberg, B. and Palmer, R. H. (2003). Jeb signals through the Alk receptor tyrosine kinase to drive visceral muscle fusion. *Nature* 425, 512-516. 10.1038/nature01950 14523447

[DEV199465C15] Frasch, M. (1995). Induction of visceral and cardiac mesoderm by ectodermal Dpp in the early Drosophila embryo. *Nature* 374, 464-467. 10.1038/374464a0 7700357

[DEV199465C16] Georgias, C., Wasser, M. and Hinz, U. (1997). A basic-helix-loop-helix protein expressed in precursors of Drosophila longitudinal visceral muscles. *Mech. Dev.* 69, 115-124. 10.1016/S0925-4773(97)00169-X 9486535

[DEV199465C17] Gramates, L. S., Marygold, S. J., Santos, G. D., Urbano, J. M., Antonazzo, G., Matthews, B. B., Rey, A. J., Tabone, C. J., Crosby, M. A., Emmert, D. B. et al. (2017). FlyBase at 25: looking to the future. *Nucleic Acids Res.* 45, D663-D671. 10.1093/nar/gkw1016 27799470 PMC5210523

[DEV199465C18] Gratz, S. J., Cummings, A. M., Nguyen, J. N., Hamm, D. C., Donohue, L. K., Harrison, M. M., Wildonger, J. and O'Connor-Giles, K. M. (2013). Genome engineering of Drosophila with the CRISPR RNA-guided Cas9 nuclease. *Genetics* 194, 1029-1035. 10.1534/genetics.113.152710 23709638 PMC3730909

[DEV199465C19] Gratz, S. J., Rubinstein, C. D., Harrison, M. M., Wildonger, J. and O'Connor-Giles, K. M. (2015). CRISPR-Cas9 Genome Editing in Drosophila. *Curr. Protoc. Mol. Biol.* 111, 31 32 31-20. 10.1002/0471142727.mb3102s111 26131852 PMC4506758

[DEV199465C20] Heinz, S., Benner, C., Spann, N., Bertolino, E., Lin, Y. C., Laslo, P., Cheng, J. X., Murre, C., Singh, H. and Glass, C. K. (2010). Simple combinations of lineage-determining transcription factors prime cis-regulatory elements required for macrophage and B cell identities. *Mol. Cell* 38, 576-589. 10.1016/j.molcel.2010.05.004 20513432 PMC2898526

[DEV199465C21] Hill, K. K., Bedian, V., Juang, J. L. and Hoffmann, F. M. (1995). Genetic interactions between the Drosophila Abelson (Abl) tyrosine kinase and failed axon connections (fax), a novel protein in axon bundles. *Genetics* 141, 595-606. 10.1093/genetics/141.2.595 8647396 PMC1206759

[DEV199465C22] Hsu, H. J. and Drummond-Barbosa, D. (2017). A visual screen for diet-regulated proteins in the Drosophila ovary using GFP protein trap lines. *Gene Expr. Patterns* 23-24, 13-21. 10.1016/j.gep.2017.01.001 28093350 PMC5392429

[DEV199465C23] Huang, H., Du, G., Chen, H., Liang, X., Li, C., Zhu, N., Xue, L., Ma, J. and Jiao, R. (2011). Drosophila Smt3 negatively regulates JNK signaling through sequestering Hipk in the nucleus. *Development* 138, 2477-2485. 10.1242/dev.061770 21561986

[DEV199465C24] Hursh, D. A., Padgett, R. W. and Gelbart, W. M. (1993). Cross regulation of decapentaplegic and Ultrabithorax transcription in the embryonic visceral mesoderm of Drosophila. *Development* 117, 1211-1222. 10.1242/dev.117.4.1211 8404526

[DEV199465C25] Kerner, P., Hung, J., Béhague, J., Le Gouar, M., Balavoine, G. and Vervoort, M. (2009). Insights into the evolution of the snail superfamily from metazoan wide molecular phylogenies and expression data in annelids. *BMC Evol. Biol.* 9, 94. 10.1186/1471-2148-9-94 19426549 PMC2688512

[DEV199465C26] Klapper, R., Stute, C., Schomaker, O., Strasser, T., Janning, W., Renkawitz-Pohl, R. and Holz, A. (2002). The formation of syncytia within the visceral musculature of the Drosophila midgut is dependent on duf, sns and mbc. *Mech. Dev.* 110, 85-96. 10.1016/S0925-4773(01)00567-6 11744371

[DEV199465C27] Kudron, M. M., Victorsen, A., Gevirtzman, L., Hillier, L. W., Fisher, W. W., Vafeados, D., Kirkey, M., Hammonds, A. S., Gersch, J., Ammouri, H. et al. (2018). The ModERN resource: genome-wide binding profiles for hundreds of Drosophila and Caenorhabditis elegans transcription factors. *Genetics* 208, 937-949. 10.1534/genetics.117.300657 29284660 PMC5844342

[DEV199465C28] Kusch, T. and Reuter, R. (1999). Functions for Drosophila brachyenteron and forkhead in mesoderm specification and cell signalling. *Development* 126, 3991-4003. 10.1242/dev.126.18.3991 10457009

[DEV199465C29] Langmead, B. and Salzberg, S. L. (2012). Fast gapped-read alignment with Bowtie 2. *Nat. Methods* 9, 357-359. 10.1038/nmeth.1923 22388286 PMC3322381

[DEV199465C30] Lécuyer, E., Parthasarathy, N. and Krause, H. M. (2008). Fluorescent in situ hybridization protocols in Drosophila embryos and tissues. *Methods Mol. Biol.* 420, 289-302. 10.1007/978-1-59745-583-1_18 18641955

[DEV199465C31] Lee, H.-H., Norris, A., Weiss, J. B. and Frasch, M. (2003). Jelly belly protein activates the receptor tyrosine kinase Alk to specify visceral muscle pioneers. *Nature* 425, 507-512. 10.1038/nature01916 14523446

[DEV199465C32] Lee, H.-H., Zaffran, S. and Frasch, M. (2006). Development of the larval visceral musculature. In *Muscle Development in Drosophila*, pp. 62-78. New York, NY: Springer New York. 10.1007/0-387-32963-3_6

[DEV199465C33] Liao, Y., Wang, J., Jaehnig, E. J., Shi, Z. and Zhang, B. (2019). WebGestalt 2019: gene set analysis toolkit with revamped UIs and APIs. *Nucleic Acids Res.* 47, W199-W205. 10.1093/nar/gkz401 31114916 PMC6602449

[DEV199465C34] Lo, P. C. H., Zaffran, S., Sénatore, S. and Frasch, M. (2007). The Drosophila Hand gene is required for remodeling of the developing adult heart and midgut during metamorphosis. *Dev. Biol.* 311, 287-296. 10.1016/j.ydbio.2007.08.024 17904115 PMC2128039

[DEV199465C35] Lorén, C. E., Scully, A., Grabbe, C., Edeen, P. T., Thomas, J., McKeown, M., Hunter, T. and Palmer, R. H. (2001). Identification and characterization of DAlk: a novel Drosophila melanogaster RTK which drives ERK activation in vivo. *Genes Cells* 6, 531-544. 10.1046/j.1365-2443.2001.00440.x 11442633 PMC1975818

[DEV199465C36] Lorén, C. E., Englund, C., Grabbe, C., Hallberg, B., Hunter, T. and Palmer, R. H. (2003). A crucial role for the Anaplastic lymphoma kinase receptor tyrosine kinase in gut development in Drosophila melanogaster. *EMBO Rep.* 4, 781-786. 10.1038/sj.embor.embor897 12855999 PMC1326337

[DEV199465C37] Lun, A. T. L. and Smyth, G. K. (2016). csaw: a Bioconductor package for differential binding analysis of ChIP-seq data using sliding windows. *Nucleic Acids Res.* 44, e45. 10.1093/nar/gkv1191 26578583 PMC4797262

[DEV199465C38] Maksimov, D. A., Laktionov, P. P. and Belyakin, S. N. (2016). Data analysis algorithm for DamID-seq profiling of chromatin proteins in Drosophila melanogaster. *Chromosome Res.* 24, 481-494. 10.1007/s10577-016-9538-4 27766446

[DEV199465C39] Marshall, O. J., Southall, T. D., Cheetham, S. W. and Brand, A. H. (2016). Cell-type-specific profiling of protein-DNA interactions without cell isolation using targeted DamID with next-generation sequencing. *Nat. Protoc.* 11, 1586-1598. 10.1038/nprot.2016.084 27490632 PMC7032955

[DEV199465C40] Martin, B. S., Ruiz-Gómez, M., Landgraf, M. and Bate, M. (2001). A distinct set of founders and fusion-competent myoblasts make visceral muscles in the Drosophila embryo. *Development* 128, 3331-3338. 10.1242/dev.128.17.3331 11546749

[DEV199465C41] Masucci, J. D. and Hoffmann, F. M. (1993). Identification of two regions from the Drosophila decapentaplegic gene required for embryonic midgut development and larval viability. *Dev. Biol.* 159, 276-287. 10.1006/dbio.1993.1240 8365566

[DEV199465C42] Mendoza-García, P., Hugosson, F., Fallah, M., Higgins, M. L., Iwasaki, Y., Pfeifer, K., Wolfstetter, G., Varshney, G., Popichenko, D., Gergen, J. P. et al. (2017). The Zic family homologue Odd-paired regulates Alk expression in Drosophila. *PLoS Genet.* 13, e1006617. 10.1371/journal.pgen.1006617 28369060 PMC5393633

[DEV199465C43] Müller, H.-A. J. (2008). Immunolabelling of embryos. *Methods Mol. Biol.* 420, 207-218. 10.1007/978-1-59745-583-1_12 18641949 PMC3513711

[DEV199465C44] Müller, J., Thüringer, F., Biggin, M., Züst, B. and Bienz, M. (1989). Coordinate action of a proximal homeoprotein binding site and a distal sequence confers the Ultrabithorax expression pattern in the visceral mesoderm. *EMBO J.* 8, 4143-4151. 10.1002/j.1460-2075.1989.tb08599.x 2574106 PMC401602

[DEV199465C45] Nose, A., Isshiki, T. and Takeichi, M. (1998). Regional specification of muscle progenitors in Drosophila: the role of the msh homeobox gene. *Development* 125, 215-223. 10.1242/dev.125.2.215 9486795

[DEV199465C46] Okamoto, N. and Nishimura, T. (2015). Signaling from glia and cholinergic neurons controls nutrient-dependent production of an insulin-like peptide for drosophila body growth. *Dev. Cell* 35, 295-310. 10.1016/j.devcel.2015.10.003 26555050

[DEV199465C47] Panganiban, G. E., Reuter, R., Scott, M. P. and Hoffmann, F. M. (1990). A Drosophila growth factor homolog, decapentaplegic, regulates homeotic gene expression within and across germ layers during midgut morphogenesis. *Development* 110, 1041-1050. 10.1242/dev.110.4.1041 1983114

[DEV199465C48] Pfeifer, K., Dorresteijn, A. W. C. and Fröbius, A. C. (2012). Activation of Hox genes during caudal regeneration of the polychaete annelid Platynereis dumerilii. *Dev. Genes Evol.* 222, 165-179. 10.1007/s00427-012-0402-z 22569931

[DEV199465C49] Popichenko, D., Hugosson, F., Sjögren, C., Dogru, M., Yamazaki, Y., Wolfstetter, G., Schönherr, C., Fallah, M., Hallberg, B., Nguyen, H. et al. (2013). Jeb/Alk signalling regulates the Lame duck GLI family transcription factor in the Drosophila visceral mesoderm. *Development* 140, 3156-3166. 10.1242/dev.094466 23824577

[DEV199465C50] Poulson, D. F. (1950). Histogenesis, organogenesis and differentiation in the embryo of Drosophila melanogaster Meigen. *Biol. Drosophila*, 168-274.

[DEV199465C51] Quinlan, A. R. (2014). BEDTools: the Swiss-army tool for genome feature analysis. *Curr. Protoc. Bioinformatics* 47, 11 12 11-34. 10.1002/0471250953.bi1112s47 PMC4213956 25199790

[DEV199465C52] Ramírez, F., Dündar, F., Diehl, S., Grüning, B. A. and Manke, T. (2014). deepTools: a flexible platform for exploring deep-sequencing data. *Nucleic Acids Res.* 42, W187-W191. 10.1093/nar/gku365 24799436 PMC4086134

[DEV199465C53] Raudvere, U., Kolberg, L., Kuzmin, I., Arak, T., Adler, P., Peterson, H. and Vilo, J. (2019). g:Profiler: a web server for functional enrichment analysis and conversions of gene lists (2019 update). *Nucleic Acids Res.* 47, W191-W198. 10.1093/nar/gkz369 31066453 PMC6602461

[DEV199465C54] Reddington, J. P., Garfield, D. A., Sigalova, O. M., Karabacak Calviello, A., Marco-Ferreres, R., Girardot, C., Viales, R. R., Degner, J. F., Ohler, U. and Furlong, E. E. M. (2020). Lineage-resolved enhancer and promoter usage during a time course of embryogenesis. *Dev. Cell* 55, 648-664 e649. 10.1016/j.devcel.2020.10.009 33171098

[DEV199465C55] Reuter, R. and Scott, M. P. (1990). Expression and function of the homoeotic genes Antennapedia and Sex combs reduced in the embryonic midgut of Drosophila. *Development* 109, 289-303. 10.1242/dev.109.2.289 1976087

[DEV199465C56] Reuter, R., Panganiban, G. E., Hoffmann, F. M. and Scott, M. P. (1990). Homeotic genes regulate the spatial expression of putative growth factors in the visceral mesoderm of Drosophila embryos. *Development* 110, 1031-1040. 10.1242/dev.110.4.1031 1983113

[DEV199465C57] Rice, P., Longden, I. and Bleasby, A. (2000). EMBOSS: the European Molecular Biology Open Software Suite. *Trends Genet.* 16, 276-277. 10.1016/S0168-9525(00)02024-2 10827456

[DEV199465C58] Roy, S., Shashidhara, L. S. and VijayRaghavan, K. (1997). Muscles in the Drosophila second thoracic segment are patterned independently of autonomous homeotic gene function. *Curr. Biol.* 7, 222-227. 10.1016/S0960-9822(06)00117-5 9094307

[DEV199465C59] Roy, S., Ernst, J., Kharchenko, P. V., Kheradpour, P., Negre, N., Eaton, M. L., Landolin, J. M., Bristow, C. A., Ma, L., Lin, M. F. et al. (2010). Identification of functional elements and regulatory circuits by Drosophila modENCODE. *Science* 330, 1787-1797. 10.1126/science.1198374 21177974 PMC3192495

[DEV199465C60] Rudolf, A., Buttgereit, D., Jacobs, M., Wolfstetter, G., Kesper, D., Pütz, M., Berger, S., Renkawitz-Pohl, R., Holz, A. and Önel, S. F. (2014). Distinct genetic programs guide Drosophila circular and longitudinal visceral myoblast fusion. *BMC Cell Biol.* 15, 27. 10.1186/1471-2121-15-27 25000973 PMC4169254

[DEV199465C61] Ruiz-Gómez, M., Coutts, N., Price, A., Taylor, M. V. and Bate, M. (2000). Drosophila dumbfounded: a myoblast attractant essential for fusion. *Cell* 102, 189-198. 10.1016/S0092-8674(00)00024-6 10943839

[DEV199465C62] Sánchez, J., Talamillo, A., Lopitz-Otsoa, F., Pérez, C., Hjerpe, R., Sutherland, J. D., Herboso, L., Rodríguez, M. S. and Barrio, R. (2010). Sumoylation modulates the activity of Spalt-like proteins during wing development in Drosophila. *J. Biol. Chem.* 285, 25841-25849. 10.1074/jbc.M110.124024 20562097 PMC2919146

[DEV199465C63] Satija, R., Farrell, J. A., Gennert, D., Schier, A. F. and Regev, A. (2015). Spatial reconstruction of single-cell gene expression data. *Nat. Biotechnol.* 33, 495-502. 10.1038/nbt.3192 25867923 PMC4430369

[DEV199465C64] Schaub, C. and Frasch, M. (2013). Org-1 is required for the diversification of circular visceral muscle founder cells and normal midgut morphogenesis. *Dev. Biol.* 376, 245-259. 10.1016/j.ydbio.2013.01.022 23380635 PMC3602240

[DEV199465C65] Schneider, C. A., Rasband, W. S. and Eliceiri, K. W. (2012). NIH Image to ImageJ: 25 years of image analysis. *Nat. Methods* 9, 671-675. 10.1038/nmeth.2089 22930834 PMC5554542

[DEV199465C66] Schröter, R. H., Buttgereit, D., Beck, L., Holz, A. and Renkawitz-Pohl, R. (2006). Blown fuse regulates stretching and outgrowth but not myoblast fusion of the circular visceral muscles in Drosophila. *Differentiation* 74, 608-621. 10.1111/j.1432-0436.2006.00080.x 17177857

[DEV199465C67] Sellin, J., Albrecht, S., Kölsch, V. and Paululat, A. (2006). Dynamics of heart differentiation, visualized utilizing heart enhancer elements of the Drosophila melanogaster bHLH transcription factor Hand. *Gene Expr. Patterns* 6, 360-375. 10.1016/j.modgep.2005.09.012 16455308

[DEV199465C68] Shigenobu, S., Kitadate, Y., Noda, C. and Kobayashi, S. (2006). Molecular characterization of embryonic gonads by gene expression profiling in Drosophila melanogaster. *Proc. Natl. Acad. Sci. USA* 103, 13728-13733. 10.1073/pnas.0603767103 16950879 PMC1559405

[DEV199465C69] Shirinian, M., Varshney, G., Lorén, C. E., Grabbe, C. and Palmer, R. H. (2007). Drosophila Anaplastic Lymphoma Kinase regulates Dpp signalling in the developing embryonic gut. *Differentiation* 75, 418-426. 10.1111/j.1432-0436.2006.00148.x 17286600

[DEV199465C70] Singari, S., Javeed, N., Tardi, N. J., Marada, S., Carlson, J. C., Kirk, S., Thorn, J. M. and Edwards, K. A. (2014). Inducible protein traps with dominant phenotypes for functional analysis of the Drosophila genome. *Genetics* 196, 91-105. 10.1534/genetics.113.157529 24172131 PMC3872200

[DEV199465C71] Southall, T. D., Gold, K. S., Egger, B., Davidson, C. M., Caygill, E. E., Marshall, O. J. and Brand, A. H. (2013). Cell-type-specific profiling of gene expression and chromatin binding without cell isolation: assaying RNA Pol II occupancy in neural stem cells. *Dev. Cell* 26, 101-112. 10.1016/j.devcel.2013.05.020 23792147 PMC3714590

[DEV199465C72] Stuart, T., Butler, A., Hoffman, P., Hafemeister, C., Papalexi, E., Mauck, W. M., III, Hao, Y., Stoeckius, M., Smibert, P. and Satija, R. (2019). Comprehensive integration of single-cell data. *Cell* 177, 1888-1902.e1821. 10.1016/j.cell.2019.05.031 31178118 PMC6687398

[DEV199465C73] Stute, C., Schimmelpfeng, K., Renkawitz-Pohl, R., Palmer, R. H. and Holz, A. (2004). Myoblast determination in the somatic and visceral mesoderm depends on Notch signalling as well as on milliways (mili(Alk)) as receptor for Jeb signalling. *Development* 131, 743-754. 10.1242/dev.00972 14757637

[DEV199465C74] Su, Y.-H., Rastegri, E., Kao, S.-H., Lai, C.-M., Lin, K.-Y., Liao, H.-Y., Wang, M.-H. and Hsu, H.-J. (2018). Diet regulates membrane extension and survival of niche escort cells for germline homeostasis via insulin signaling. *Development* 145, dev159186. 10.1242/dev.159186 29549109

[DEV199465C75] Sun, L. V., Chen, L., Greil, F., Negre, N., Li, T.-R., Cavalli, G., Zhao, H., Van Steensel, B. and White, K. P. (2003). Protein-DNA interaction mapping using genomic tiling path microarrays in Drosophila. *Proc. Natl. Acad. Sci. USA* 100, 9428-9433. 10.1073/pnas.1533393100 12876199 PMC170935

[DEV199465C76] Tarasov, A., Vilella, A. J., Cuppen, E., Nijman, I. J. and Prins, P. (2015). Sambamba: fast processing of NGS alignment formats. *Bioinformatics (Oxford, England)* 31, 2032-2034. 10.1093/bioinformatics/btv098 PMC4765878 25697820

[DEV199465C77] Thurmond, J., Goodman, J. L., Strelets, V. B., Attrill, H., Gramates, L. S., Marygold, S. J., Matthews, B. B., Millburn, G., Antonazzo, G., Trovisco, V. et al. (2019). FlyBase 2.0: the next generation. *Nucleic Acids Res.* 47, D759-D765. 10.1093/nar/gky1003 30364959 PMC6323960

[DEV199465C78] Tosti, L., Ashmore, J., Tan, B. S. N., Carbone, B., Mistri, T. K., Wilson, V., Tomlinson, S. R. and Kaji, K. (2018). Mapping transcription factor occupancy using minimal numbers of cells in vitro and in vivo. *Genome Res.* 28, 592-605. 10.1101/gr.227124.117 29572359 PMC5880248

[DEV199465C79] Varshney, G. K. and Palmer, R. H. (2006). The bHLH transcription factor Hand is regulated by Alk in the Drosophila embryonic gut. *Biochem. Biophys. Res. Commun.* 351, 839-846. 10.1016/j.bbrc.2006.10.117 17094947

[DEV199465C80] Wolf, F. A., Angerer, P. and Theis, F. J. (2018). SCANPY: large-scale single-cell gene expression data analysis. *Genome Biol.* 19, 15. 10.1186/s13059-017-1382-0 29409532 PMC5802054

[DEV199465C81] Wolfstetter, G., Pfeifer, K., van Dijk, J. R., Hugosson, F., Lu, X. and Palmer, R. H. (2017). The scaffolding protein Cnk binds to the receptor tyrosine kinase Alk to promote visceral founder cell specification in Drosophila. *Sci. Signal.* 10. 10.1126/scisignal.aan0804 29066538

[DEV199465C82] Wu, F., Olson, B. G. and Yao, J. (2016). DamID-seq: genome-wide mapping of protein-DNA interactions by high throughput sequencing of adenine-methylated DNA fragments. *J Vis Exp* e53620. 10.3791/53620 26862720 PMC4781701

[DEV199465C83] Yang, F., Moss, L. G. and Phillips, G. N. Jr. (1996). The molecular structure of green fluorescent protein. *Nat. Biotechnol.* 14, 1246-1251. 10.1038/nbt1096-1246 9631087

[DEV199465C84] Yu, G., Wang, L.-G. and He, Q.-Y. (2015). ChIPseeker: an R/Bioconductor package for ChIP peak annotation, comparison and visualization. *Bioinformatics (Oxford, England)* 31, 2382-2383. 10.1093/bioinformatics/btv145 25765347

[DEV199465C85] Zaffran, S., Kuchler, A., Lee, H. H. and Frasch, M. (2001). biniou (FoxF), a central component in a regulatory network controlling visceral mesoderm development and midgut morphogenesis in Drosophila. *Genes Dev.* 15, 2900-2915. 11691840 10.1101/gad.917101 PMC312807

[DEV199465C86] Zhou, Y., Popadowski, S. E., Deutschman, E. and Halfon, M. S. (2019). Distinct roles and requirements for Ras pathway signaling in visceral versus somatic muscle founder specification. *Development* 146, dev169003. 10.1242/dev.169003 30630823

