# Supplementary material for: DamID transcriptional profiling identifies the Snail/Scratch transcription factor Kahuli as an Alk target in the Drosophila visceral mesoderm
Source: Development. 2021 Dec 14;148(23):dev199465. doi: 10.1242/dev.199465 (PMC8722224; doi:10.1242/dev.199465)
Supplement: Supplementary information [file develop-148-199465-s1.pdf]

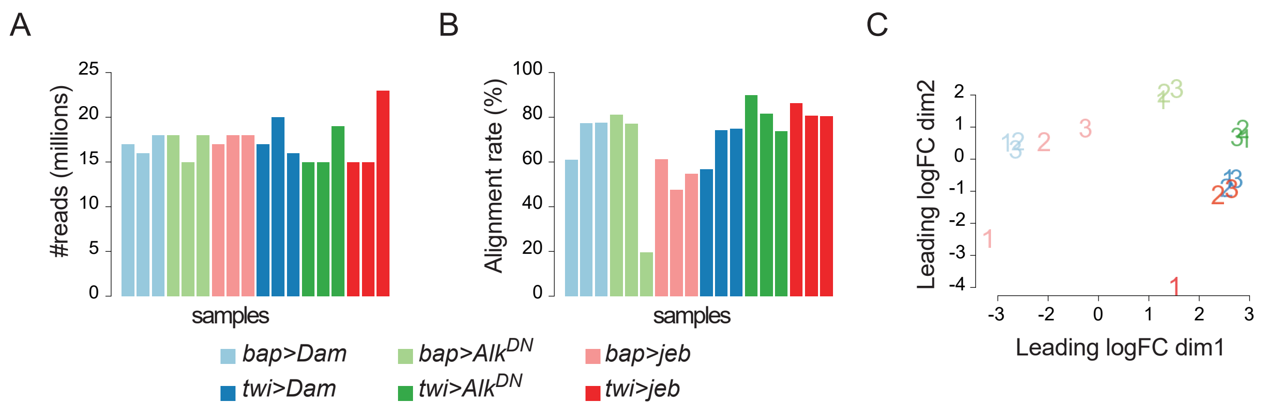

Fig. S1. A. Number of reads per DamID sample/replicate. **B.** Alignment rate of the DamID reads to the *Drosophila melanogaster* genome (dm6). **C.** Multi-dimensional scaling (MDS) plot of DamID samples.

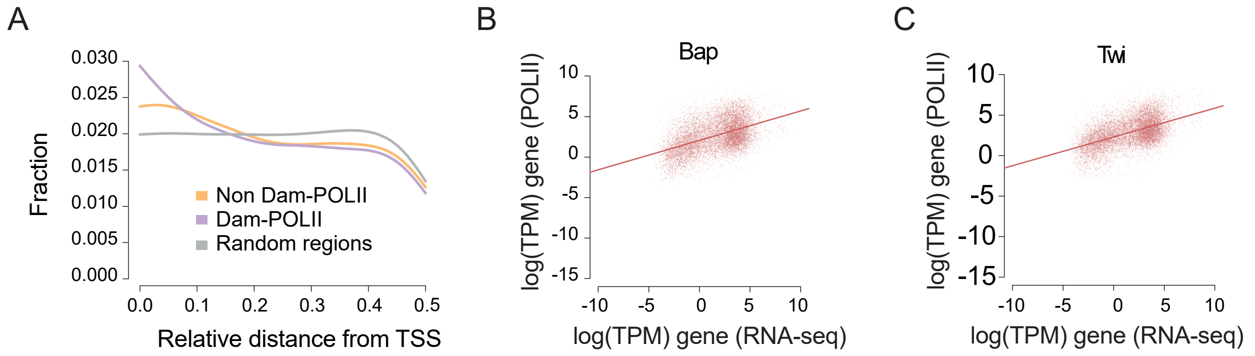

Fig. S2. A. Alignment rate of the Dam-Pol II occupied sites compared to Non Dam-Pol II and random genomic regions. **B-C.** Correlation graph between DamID and RNA-seq (from mesodermal cells).

Development • Supplementary information

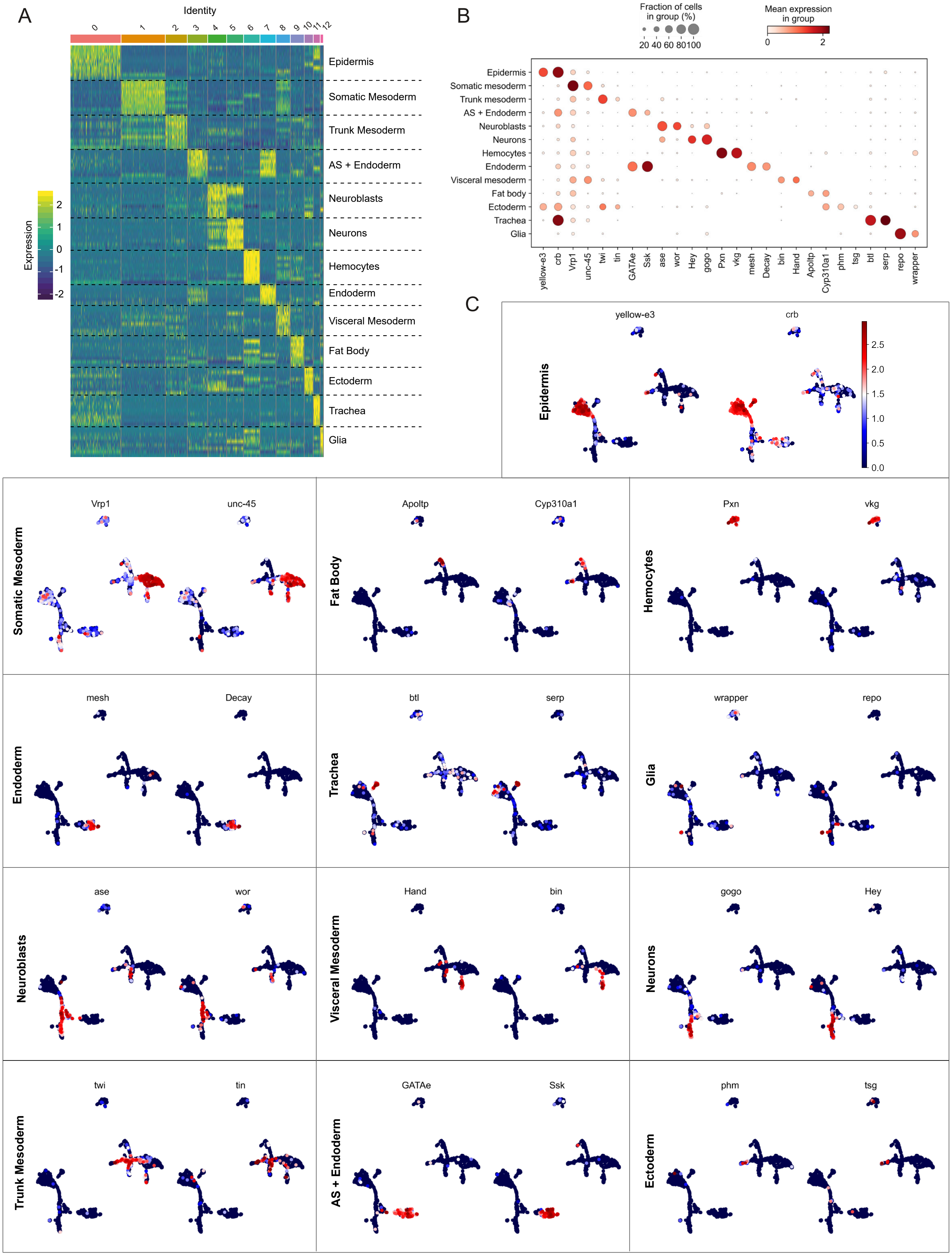

Development • Supplementary information

Fig. S3. A. Initial heat map analysis of whole embryo ScRNA-Seq data indicating 13 cell clusters. **B.** Dot plot displays genes identified as canonical markers to determine the cellular heterogeneity of the whole embryo scRNA-seq dataset. **C.** Feature Plots visualize the gene expression of a selected pair of canonical markers (shown as UMAP), across the population of whole embryo scRNA-seq dataset. Color scale indicates low (blue) to high (red) expression levels.

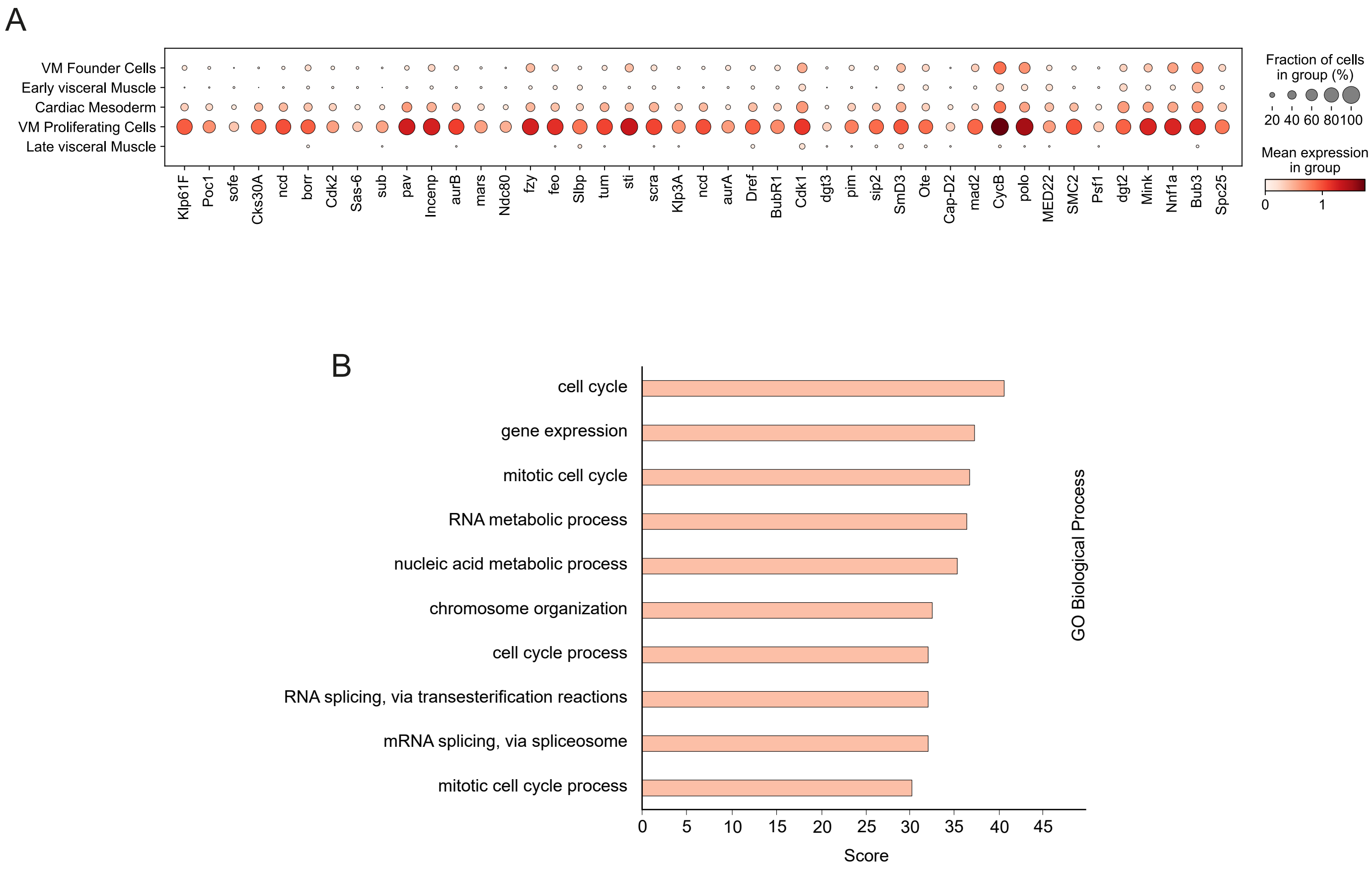

Fig. S4. A. Dot plot representing the percentage of cell distribution and expression levels in the VM proliferating cells in the *HandC-GFP* single cell dataset. These candidates are considered as notable markers of VM proliferating cells. **B.** Gene Ontology (GO) enrichment analysis for VM proliferating cells.

Development • Supplementary information

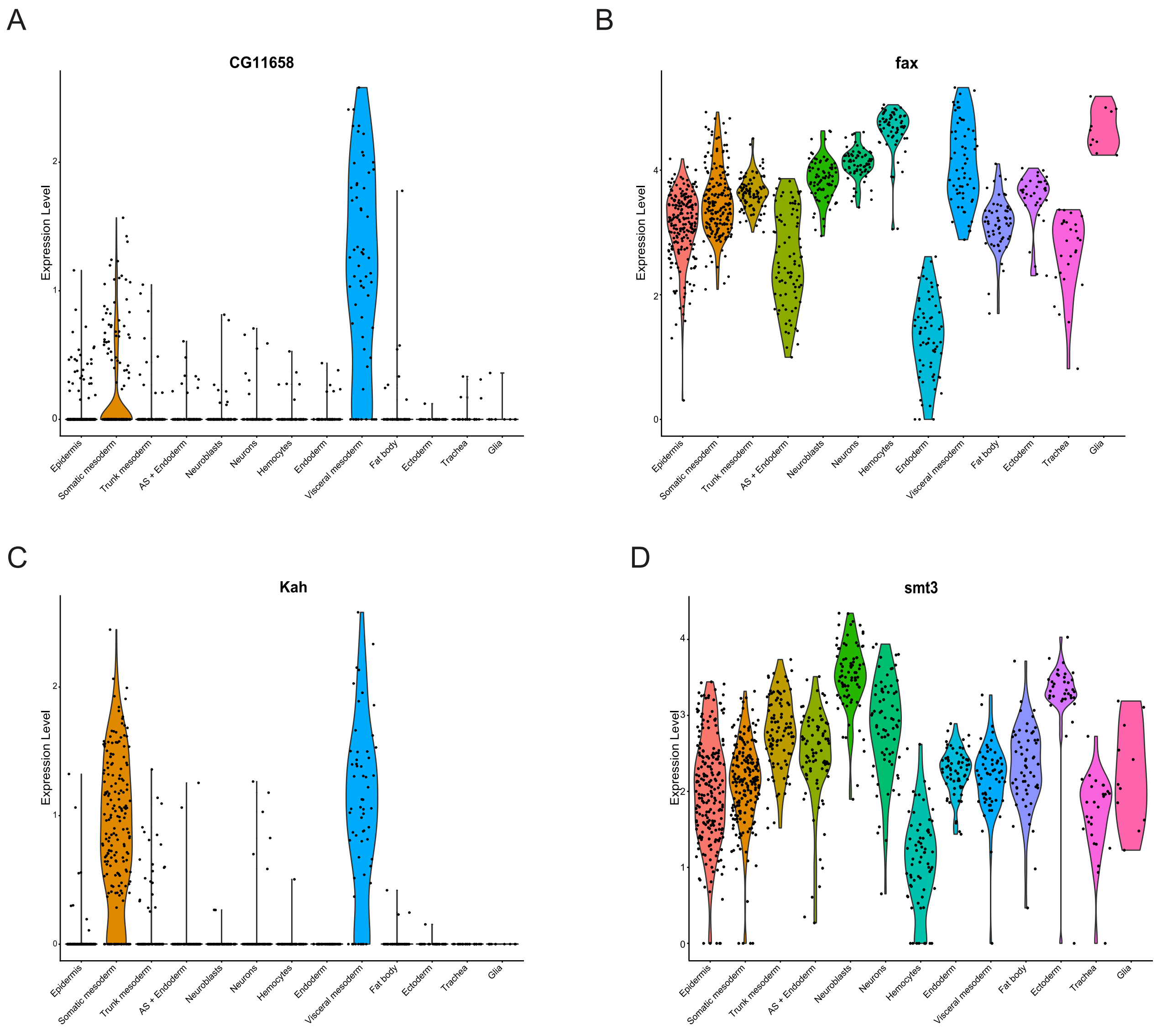

Fig. S5. Violin plots showing the mRNA expression of the TaDa candidates, CG11658 (A), failed axon connections (fax, B), Kahuli (Kah, C) and the SUMO family protein (smt3, D). Expression across the violin plot is shown for each population of the thirteen cell clusters (Epidermis, Somatic mesoderm, Trunk mesoderm, AS + Endoderm, Neuroblasts, Neurons, Hemocytes, Endoderm, Visceral mesoderm, Fat body, Ectoderm, Trachea and Glia) of the whole embryo scRNA-seq dataset.

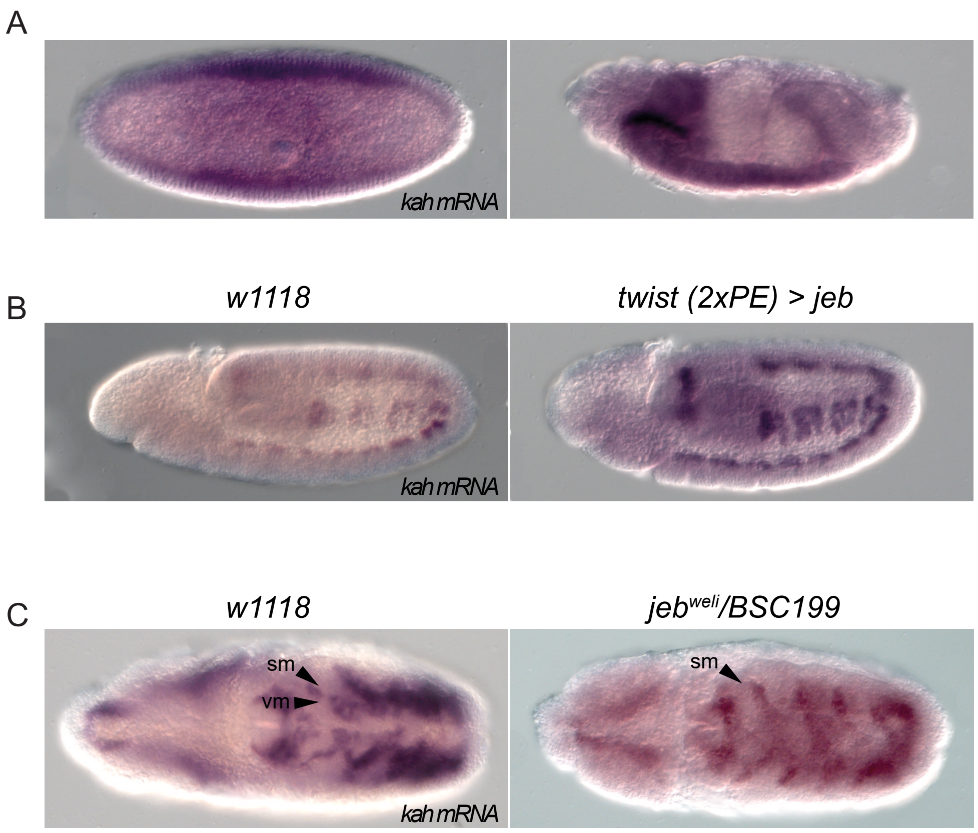

Fig. S6. A. *Kah* mRNA expression in non-mesodermal tissues in the segmented region in early embryogenesis and in the VNC and CNS in late embryos. **B.** Overexpression of jeb results in higher *Kah* expression. **C.** Embryos devoid of Jeb ligand show reduced *Kah* mRNA in the visceral mesoderm, but not in the somatic mesoderm.

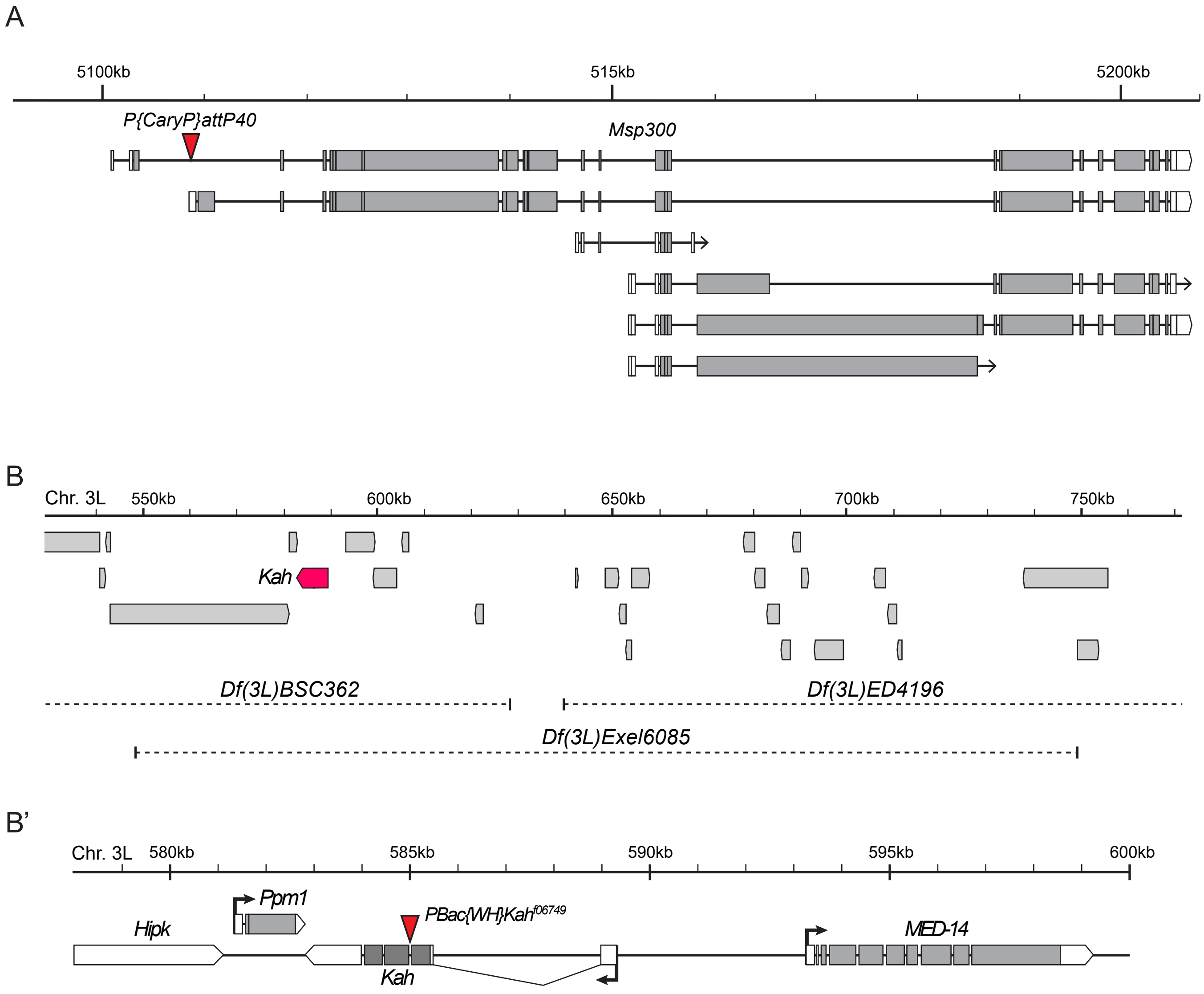

Fig. S7. A. *Kah.GFP* insertion site within the *Msp300* locus. **B.** Overview of the deficiency lines used in this study in relation to *Kah* locus. **B'.** Representation of the *Kah*^{f06749} alelle generated by insertion of a PiggyBac in the second intron of *Kah* gene.

Development • Supplementary information

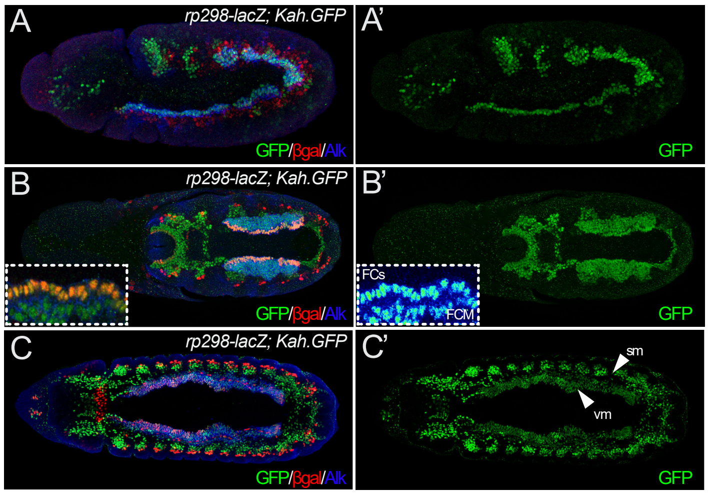

Fig. S8. Kah.GFP, encoded by *Kah-GFP.FPTB* (see Fig. S7A), is detected from stage 10 embryos in the VM, with no clear distinction between FCs (marked by *rp298-lacZ*, red, inset depicts a close-up in LUT colors) and FCMs. Lateral view (**A-A'**), dorsal view (**B-B'**). **C-C'.** Kah.GFP is still maintained in visceral (vm) and somatic musculature (sm) after myoblast fusion (stage 13). Dorsal view.

Development • Supplementary information

A

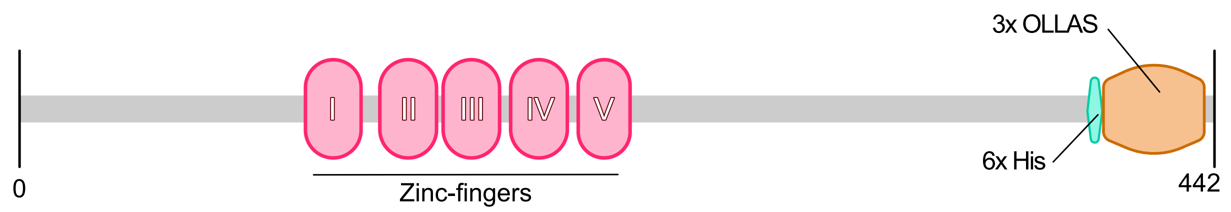

B

MTKDQQATPIPEELYNLTQLAEVTLSVGPLVTDEVKPLPLYASSDDDSNYYSHKVFDRRKLRRCT
ISDSNSCASSSSSSSTSSRQSSEDHLGLQGHSSVHHHHGEQGEILNSTSLLEDE**HICPECGKKYST**
SSNLARHRQTHRSIMDKKA**RHCPYCEKVYVSMPAYSMHVRTH**NQG**CECQFCGKRFSRPWLLQGHI**
RTHTGEKP**FKCGVCEKAFADKSNLRAHIQTH**SNTKP**HTCARCGKAFALKSYLYKHEE**SSCMKNRG
GVPGSGAASGNRPPSSPKRQQAEVTSGTISALAPGSPAAAVCAASDSAKSTLANKLLQKEKDRRQ
AAMAFQGFPAGPEVTAYSHATSAQEEYEKFKRINVIQPKVMPHRVPSLYQDLLPNRHVPLALPLA
MPYHFQGQATSTGQSDPTSVQEQPVDFSPKNNFTHSAKTSPFELTGNYAMVA**GGGGGG****SGFANEL**
GPRLMGKSGFANELGPRLMGKSGFANELGPRLMGK*

Fig. S9. A. Graphic representation of the Kah$^{Cterm.OLLAS}$ protein. **B.** Protein sequence of the Kah$^{Cterm.OLLAS}$ alelle. Protein domains are highlighted: zinc-fingers (pink), 6x His (turquoise) and 3xOLLAS (brown).

Development • Supplementary information

A

Genotype	*viable/non-viable*
Exel6085/Kah_ATG	viable
Exel6085/Kah_ZnF	viable
Kah_ATG/Kah_ZnF	viable
Kah_f06749/Exel6085	non-viable
Kah_f06749/Kah_ATG	viable
Kah_f06749/Kah_ZnF	non-viable

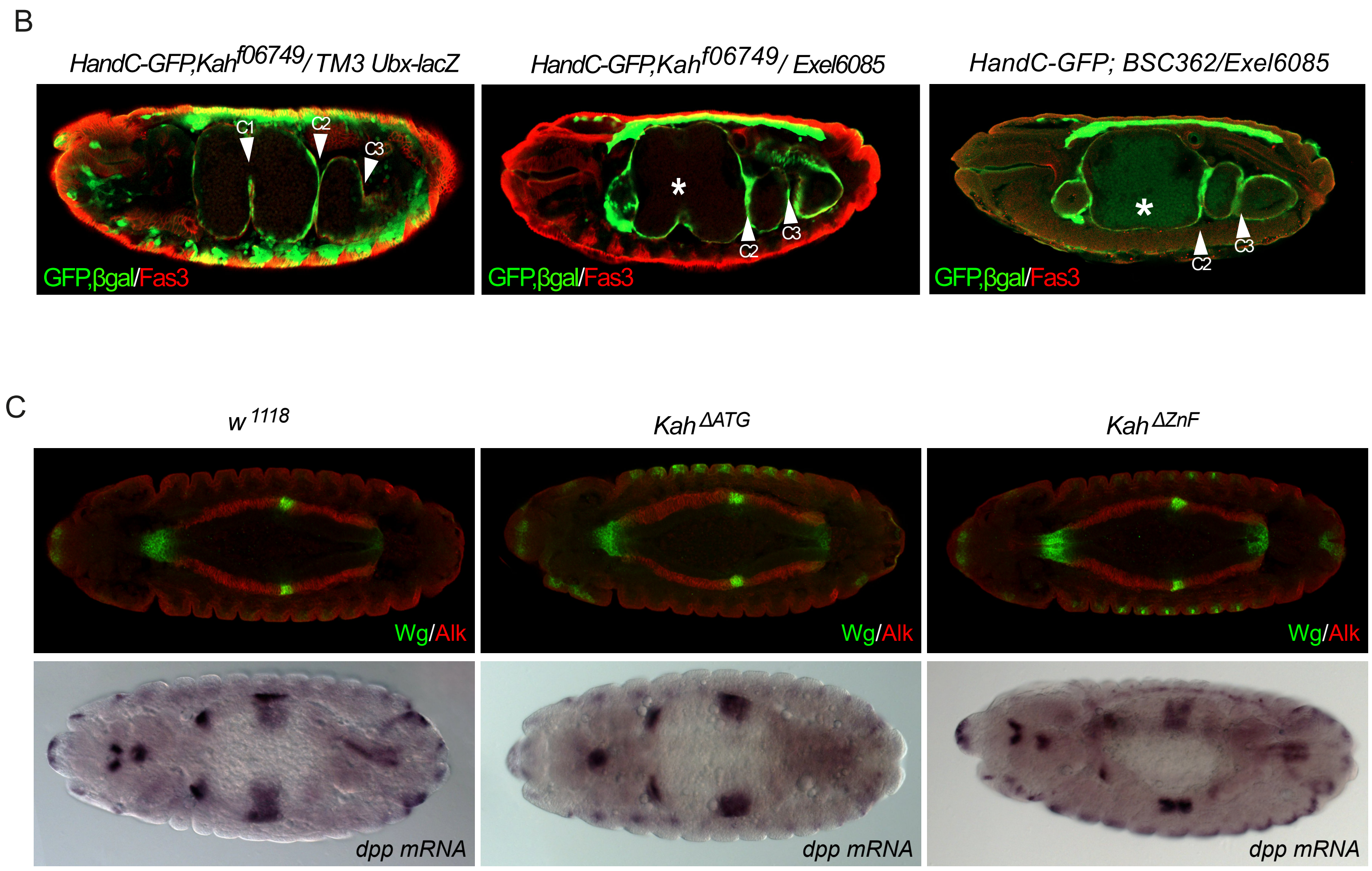

Fig. S10. A. Table summarising viability of *Kah* alleles employed in this study in combination with other Kah alleles and the publically available *Exel6085* deficiency line. **B.** *Kah^{f06749}/Exel6085* and *Exel6085/BSC362* embryos share the midgut phenotype observed in *Kah* mutants. While control embryos display three constrictions (c1, c2, c3) mutant embryos only display c2 and c3, resulting in an enlarged first chamber. **C.** *Kah* mutants display a midgut constriction phenotype (upper panel, highlighted with asterisk). Stage 16 embryos stained with Fasciclin 3 (Fas3). *Kah* mutants express Wg protein (red, middle panel) and *dpp* mRNA (lower panel) in the VM at levels comparable with control (*w^{1118}*) embryos. Stage 13, dorsal views.

Development • Supplementary information

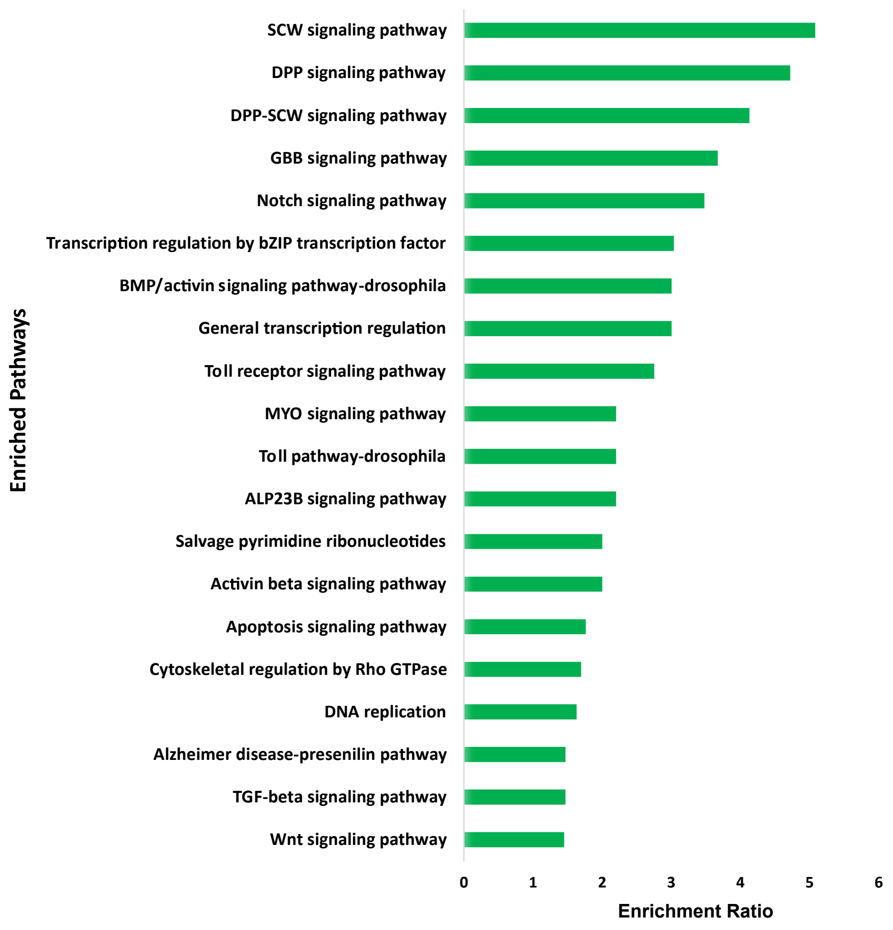

Fig. S11. Panther pathway analysis for the genes which are commonly upregulated in RNASeq datasets obtained from *Kah*$^{\Delta ATG}$ and *Kah*$^{\Delta ZnF}$ mutants.

Development • Supplementary information

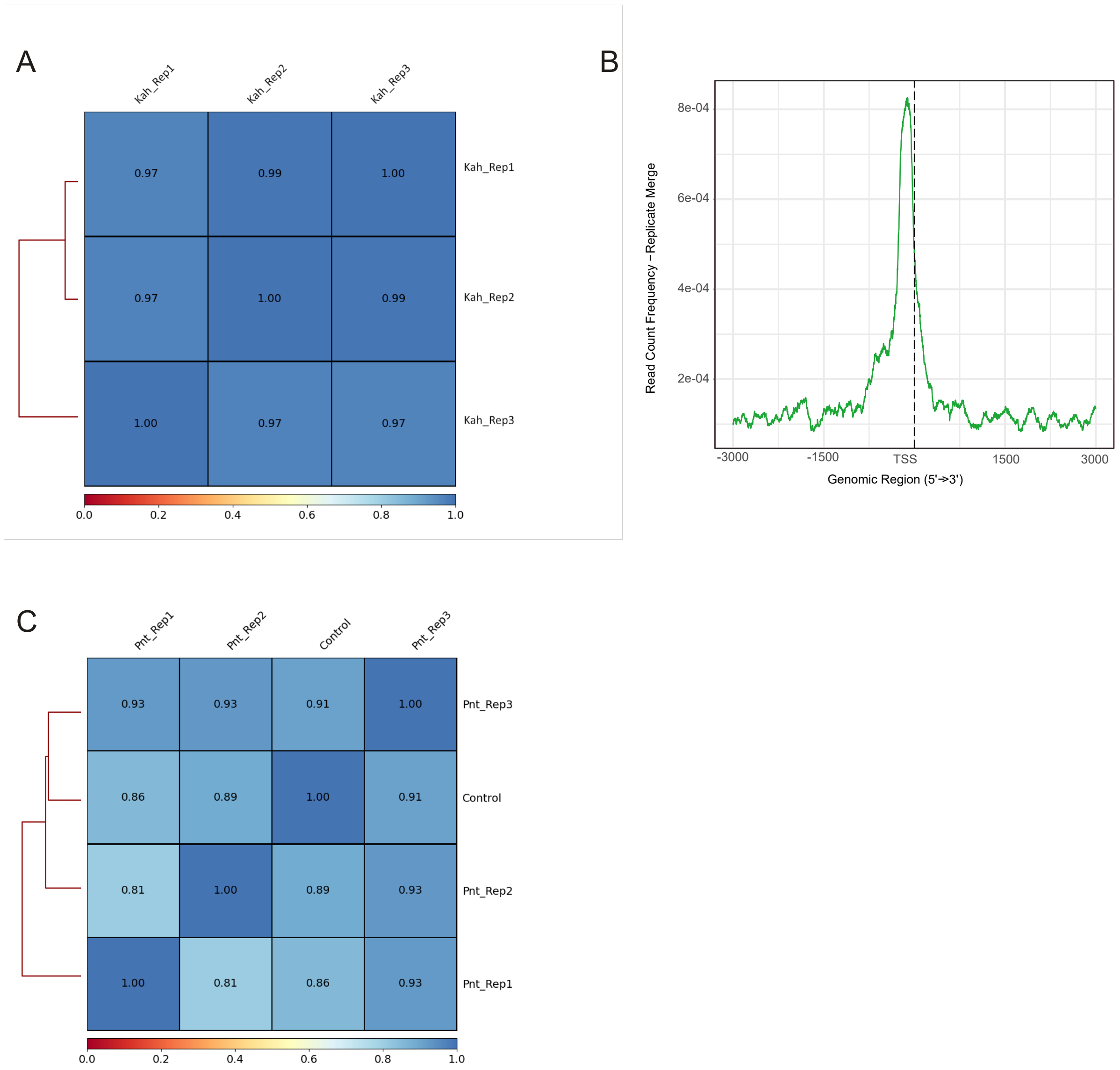

Fig. S12. A. Correlation graph for Kah-ChIP between the three experimental ChIP replicates from the modENCODE project (accession #ENCSR161YRO). **B.** Signal intensity plot indicating a peak of Kah-ChIP read counts upstream of transcriptional start sites (TSS). **C.** Correlation graph for Pnt-ChIP between the three experimental ChIP replicates and the control sample from the modENCODE project (accession #ENCSR997UIM).

Development • Supplementary information

Table S1. Summary table of TaDa analysis

Click here to download Table S1

Table S2. Summary table of RNA-Seq differential expression analysis in *Kah*$^{\Delta ATG}$ and *Kah*$^{\Delta ZnF}$ mutants

Click here to download Table S2

Table S3. Summary table of Kah-GFP and Pnt-GFP ChIP-seq analysis together with overlap with RNA-Seq differential expression analysis in *Kah*$^{\Delta ATG}$ and *Kah*$^{\Delta ZnF}$ mutants

Click here to download Table S3

Table S4. Summary table of single guide RNA (sgRNA) target sequences employed in this study

Click here to download Table S4

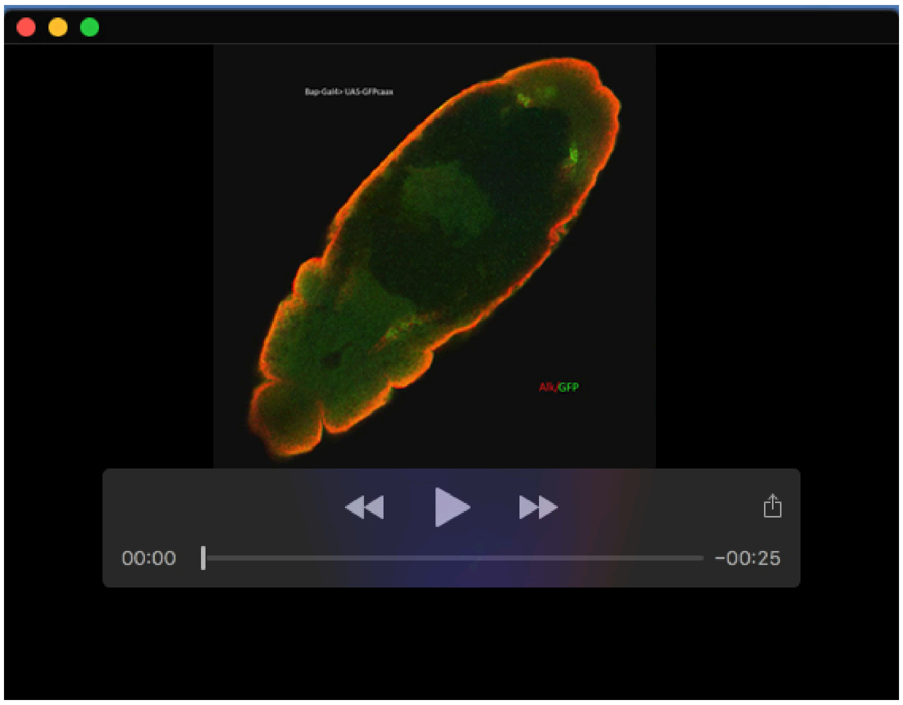

Movie 1. LSM confocal Z-stack movie of *twist2xPE-Gal4> UAS-GFPcaax* embryo stage 9. Expression of GFP predominantly in trunk mesoderm.

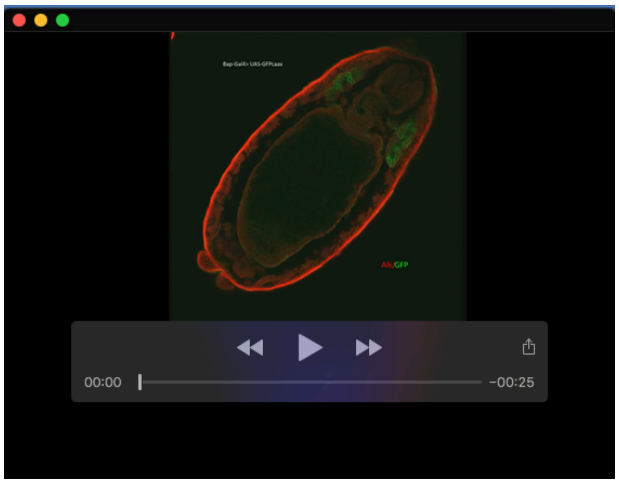

Movie 2. LSM confocal Z-stack movie of *twist2xPE-Gal4> UAS-GFPcaax* embryo stage 14. Expression of GFP in developing gut, somatic muscles and salivary glands.

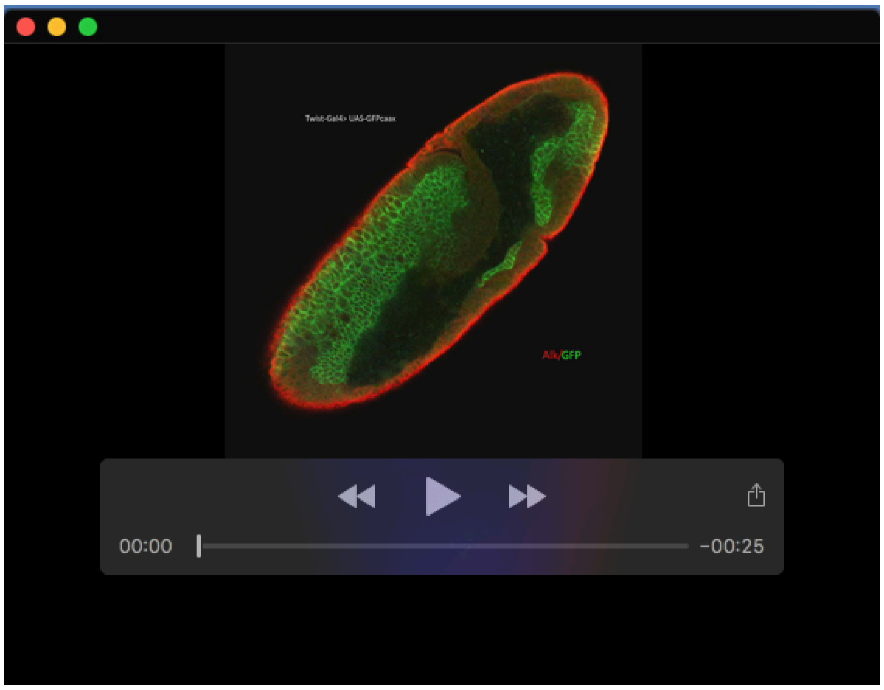

Movie 3. LSM confocal Z-stack movie of *bap-Gal4> UAS-GFPcaax* embryo stage 9. Expression of GFP predominantly in visceral mesoderm.

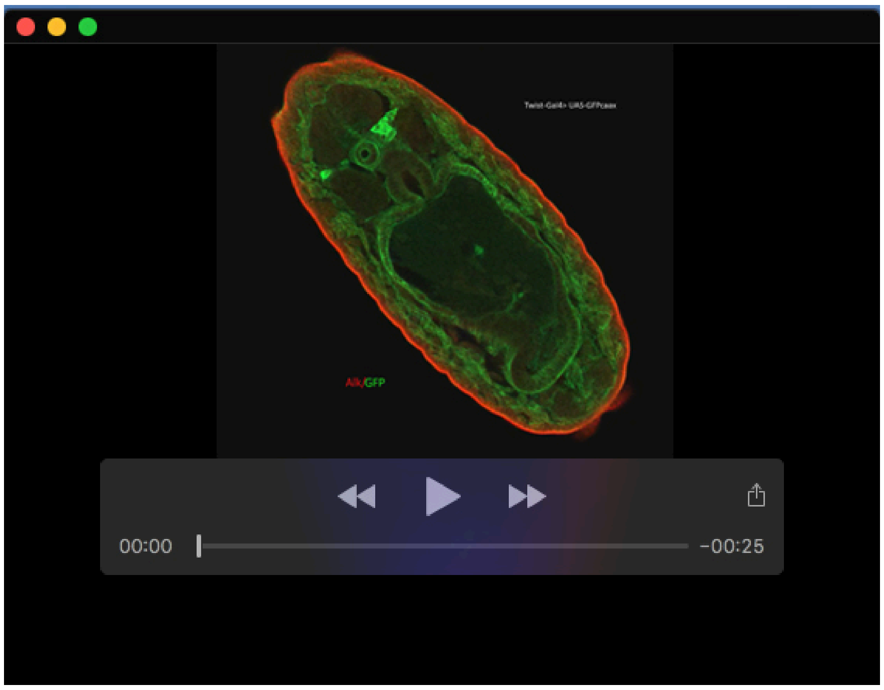

Movie 4. LSM confocal Z-stack movie of *bap-Gal4> UAS-GFPcaax* embryo stage 14. Expression of GFP in salivary glands.

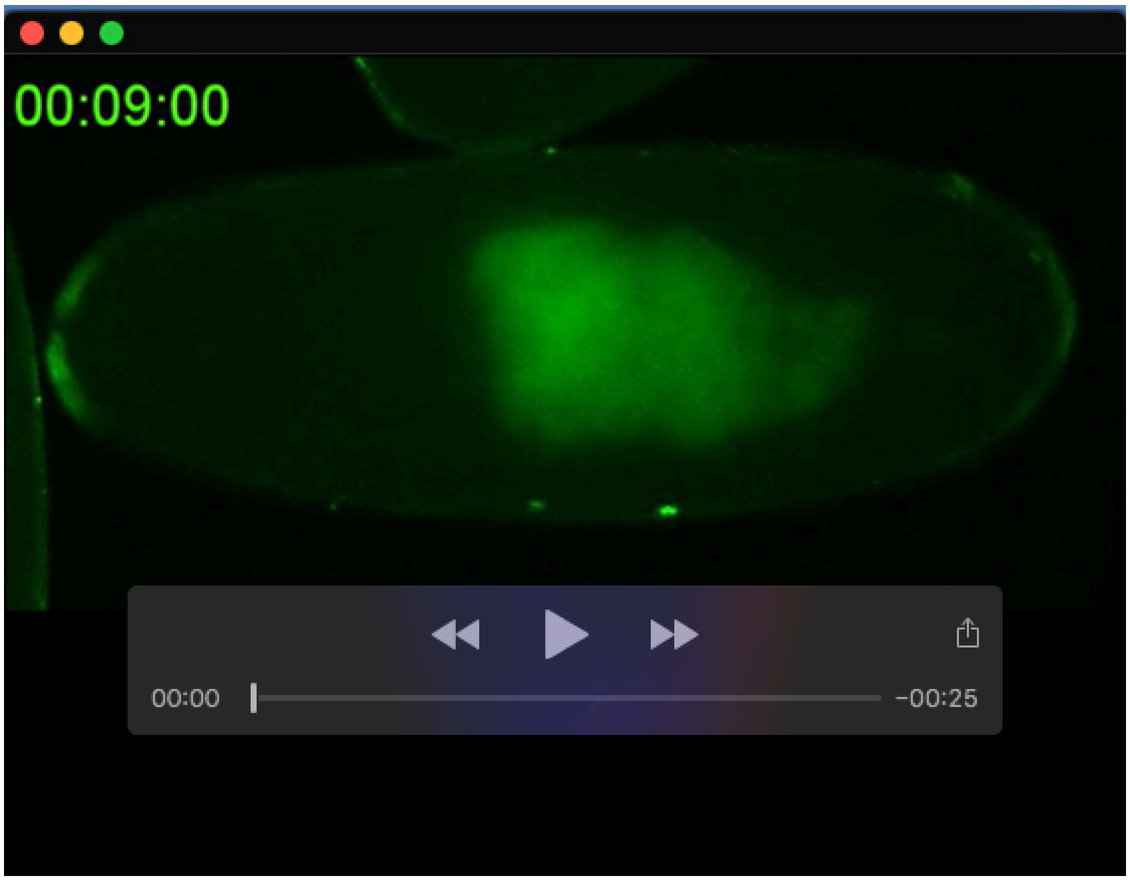

Movie 5. Live imaging of w[1118] (control) embryo staged late 15 onward for 150 min. Four mid gut chambers are generated after three successful constrictions followed by rotation of the chambers.

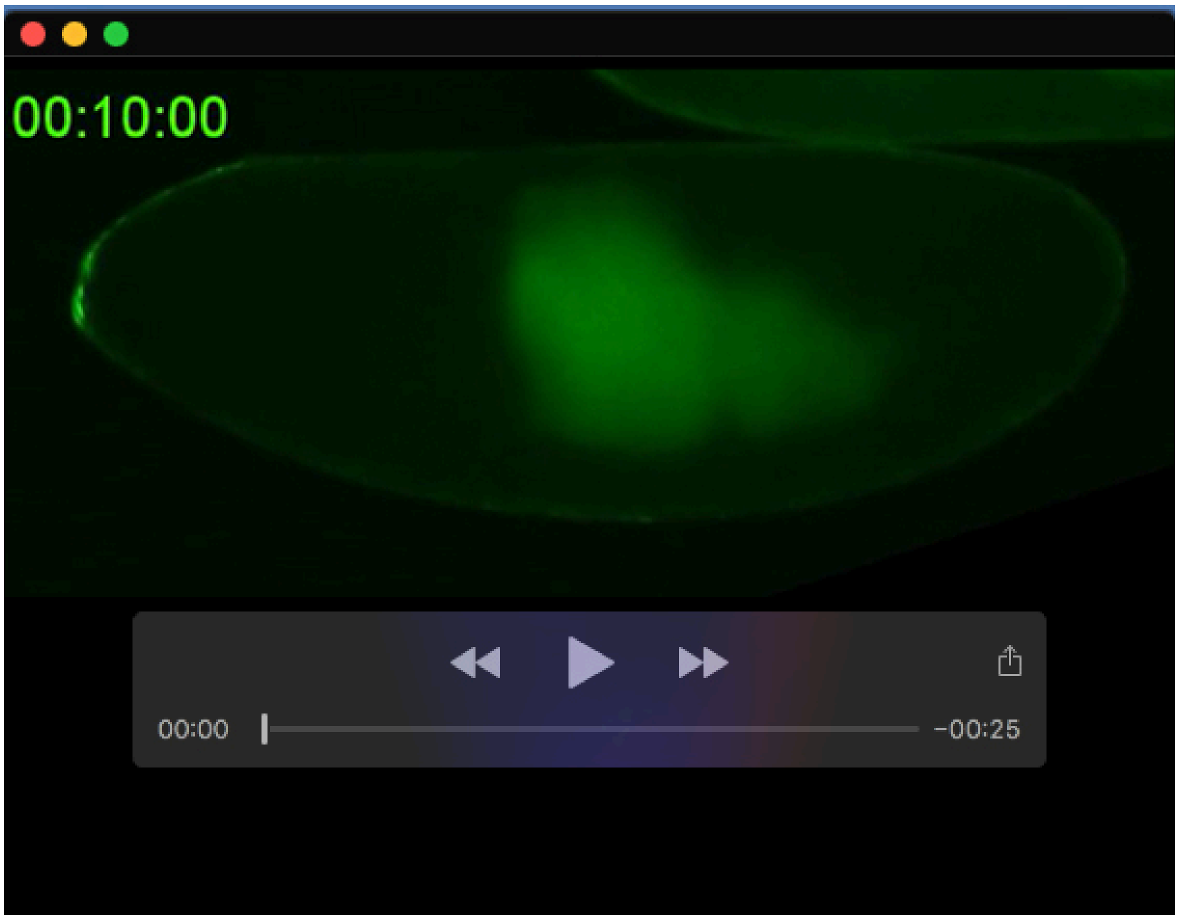

Movie 6. Live imaging of *Kah*$^{\Delta ATG}$ mutant staged late 15/early 16 onward for 150 min. First constriction started but not completed.

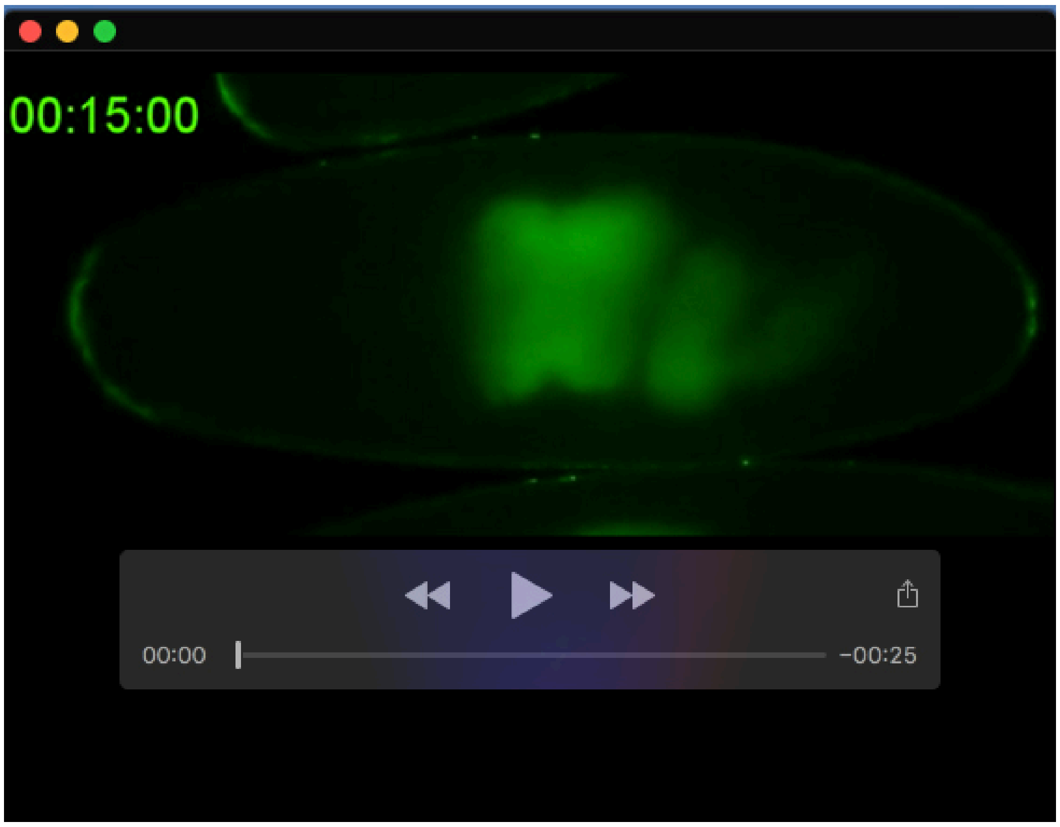

Movie 7. Live imaging of *Kah*$^{\Delta ZnF}$ mutant staged early 16 onward for 150 min. First constriction started but not completed.

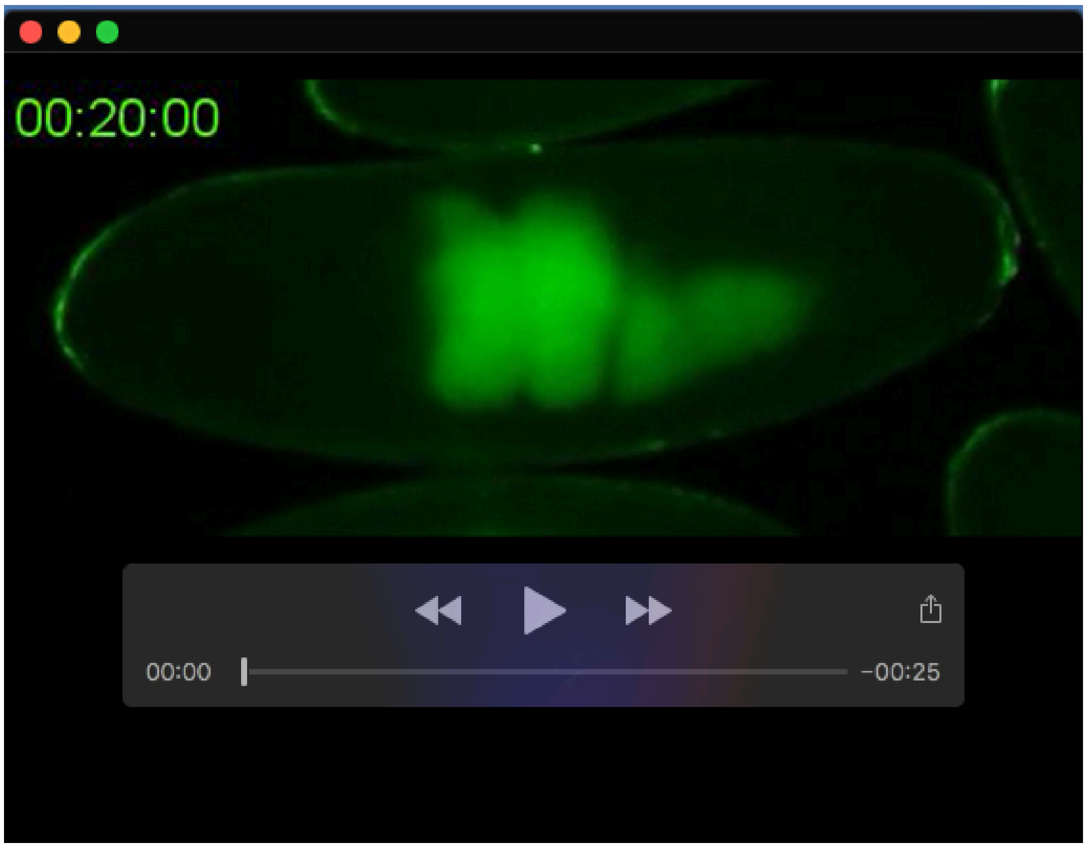

Movie 8. Live imaging of *Pnt*$^{\Delta 88F}$ mutant **staged early 16 onward for 150 min.** First constriction started but not completed.

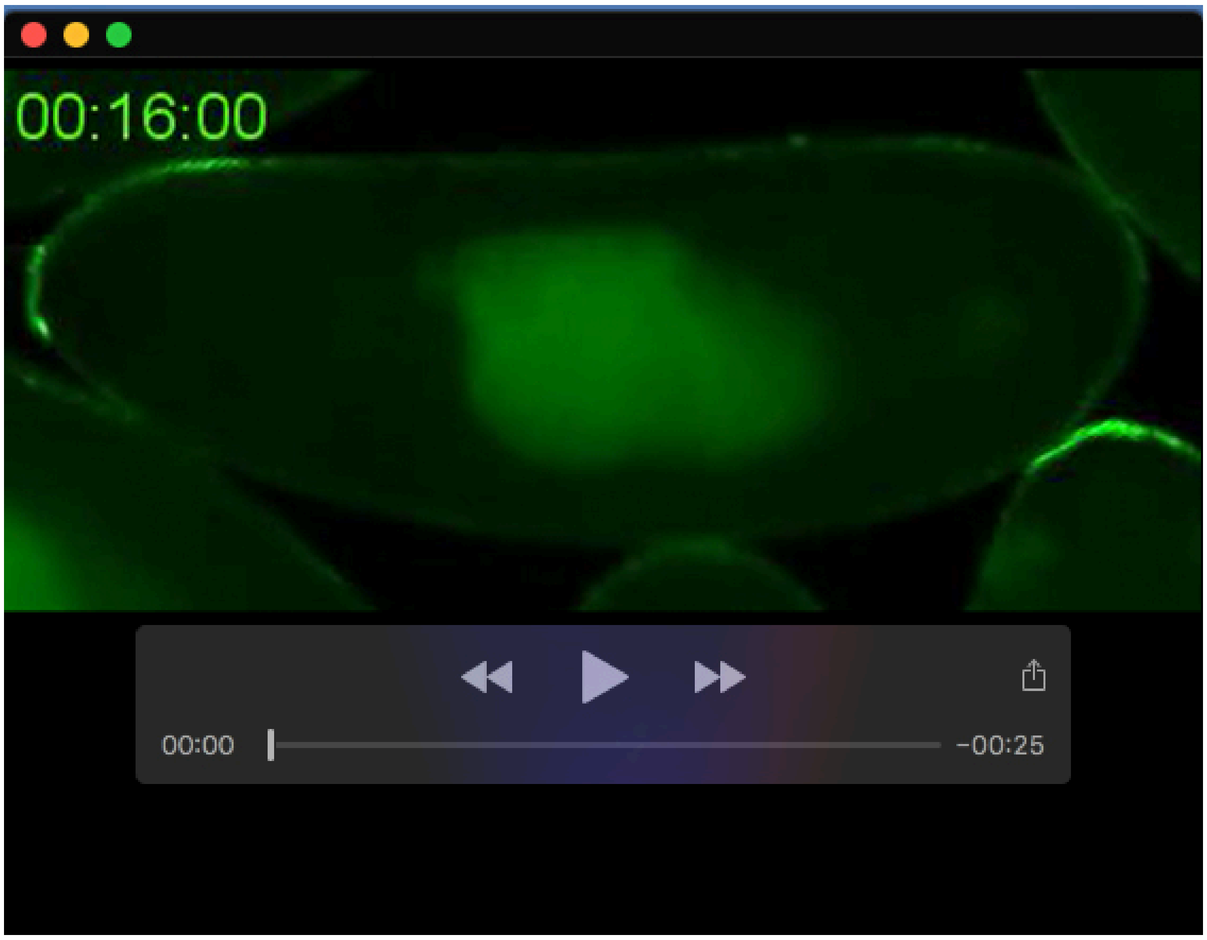

Movie 9. Live imaging of *Kah*$^{\Delta ATG}$ *Pnt*$^{\Delta 88F}$ double mutant staged early 15 onward for 250 min. Although live imaging of the double mutant was performed longer than others no apparent constriction was found.

Development • Supplementary information
