# Supplementary material for: DamID transcriptional profiling identifies the Snail/Scratch transcription factor Kahuli as an Alk target in the Drosophila visceral mesoderm
Source: Development. 2021 Dec 14;148(23):dev199465. doi: 10.1242/dev.199465 (PMC8722224; doi:10.1242/dev.199465)
Supplement: Reviewer comments [file develop-148-199465_review_history.pdf]

Development

DamID transcriptional profiling identifies the Snail/Scratch transcription factor Kahuli as an Alk target in the *Drosophila* visceral mesoderm

Patricia Mendoza-Garcia, Swaraj Basu, Sanjay Kumar Sukumar, Badrul Arefin, Georg Wolfstetter, Vimala Anthonydhason, Linnea Molander, Ezgi Uçkun, Henrik Lindehell, Christina Lebrero-Fernandez, Jan Larsson, Erik Larsson, Mats Bemark and Ruth H. Palmer
DOI: 10.1242/dev.199465

Editor: Thomas Lecuit

Review timeline

Original submission:	25 January 2021
Editorial decision:	13 April 2021
First revision received:	26 July 2021
Accepted:	26 October 2021

Original submission

First decision letter

MS ID#: DEVELOP/2021/199465

MS TITLE: DamID transcriptional profiling identifies the Snail/Scratch transcription factor Kahuli as Alk target in the Drosophila visceral mesoderm.

AUTHORS: Patricia Mendoza-Garcia, Swaraj Basu, Sanjay Kumar Sukumar, Badrul Arefin, Georg Wolfstetter, Vimala Anthonydhason, Linnea Molander, Henrik Lindehell, Jan Larsson, Erik Lekholm Larsson, Mats Bemark, and Ruth H Palmer

I very sincerely apologise for the unsual delay before being able to get back to you. I have now received all the referees' reports on the above manuscript, and have reached a decision. The referees' comments are appended below, or you can access them online: please go to BenchPress and click on the 'Manuscripts with Decisions' queue in the Author Area.

As you will see, the referees express somewhat contrasted views on your manuscript but do recognise the potential importance of your work, as do I. They have some significant criticisms and recommend a substantial revision of your manuscript before we can consider publication. If you are able to revise the manuscript along the lines suggested, which should involve further experiments, I will be happy receive a revised version of the manuscript. Your revised paper will be re-reviewed by one or more of the original referees, and acceptance of your manuscript will depend on your addressing satisfactorily the reviewers' major concerns. Please also note that Development will normally permit only one round of major revision.

We are aware that you may be experiencing disruption to the normal running of your lab that make experimental revisions challenging. If it would be helpful, we encourage you to contact us to discuss your revision in greater detail. Please send us a point-by-point response indicating where you are able to address concerns raised (either experimentally or by changes to the text) and where you will not be able to do so within the normal timeframe of a revision. We will then provide further guidance. Please also note that we are happy to extend revision timeframes as necessary.

© 2021. Published by The Company of Biologists under the terms of the Creative Commons Attribution License (https://creativecommons.org/licenses/by/4.0/).

Please attend to all of the reviewers' comments and ensure that you clearly highlight all changes made in the revised manuscript. Please avoid using 'Tracked changes' in Word files as these are lost in PDF conversion. I should be grateful if you would also provide a point-by-point response detailing how you have dealt with the points raised by the reviewers in the 'Response to Reviewers' box. If you do not agree with any of their criticisms or suggestions please explain clearly why this is so.

Reviewer 1

Advance summary and potential significance to field

The Manuscript by Palmer and colleagues describes the identification of a member of the Snail family transcription factor Kahuli (Kah) as a target of the Anaplastic Lymphoma Kinase (Alk) pathway and its role in mesoderm development of the Drosophila embryo. The Palmer group had previously identified the Alk homologue in flies and discovered a crucial role of this pathway in visceral mesoderm development. Previous Work from the Palmer and other laboratories had identified a small group of Alk target genes and described their roles in the specification of visceral muscle founder cells, but a comprehensive analysis of the transcriptional targets of the Alk pathway remained elusive. In the present work, the authors employ an elegant combination of techniques to search for transcriptional targets of the Alk tyrosine receptor signaling pathway in the Drosophila embryo. They describe the Alk-dependent differential gene expression landscape by using a targeted DamID (TaDa) approach. Among the loci with significant changes in Dam-Pol-II binding they reconfirmed genes previously known to be regulated by Alk providing a proof-of-principle. To provide further evidence for the specificity of the data set, they set out to examine the expression of the candidate target genes in distinct mesoderm cell lineages. Cluster analysis of single-cell RNAseq experiments revealed an enrichment of candidate Alk target genes in clusters of founder cells and early visceral mesoderm. The remaining of this manuscript focuses on the role of one new Alk target gene, kahuli (kah) coding a Snail family transcription factor. The authors provide evidence that kah is expressed in the visceral mesoderm and that its expression is regulated by Alk signaling. Mutational analysis of kah demonstrated that kah does not affect early visceral mesoderm development, but is required for midgut constrictions in late embryonic stages. They found that the function of kah in this process is independent of Wg and Dpp, but Kah functions in concert with another Erk-dependent transcriptional regulator, Pointed (Pnt). The authors then describe an approach to identify Kah target genes by an RNA-seq and compared these data with Pnt ChIP-seq data, which identified a set of genes that may be regulated by Kah and Pnt together. Finally, sourcing publicly available ChIP data led them to identify a potential de novo binding motif for Kah and overlapping transcriptional targets using a Pnt ChIP data set. The authors propose a model in which Kah as a direct transcriptional target of the Alk pathway then acts together with Pnt to activate target genes involved in midgut constrictions and provide evidence for Antennapedia expression in the visceral mesoderm being regulated by Kah.
The advances this paper reports are a) the identification of candidate Alk target genes; b) identification and phenotypic analysis of Kah as an Alk target gene; c) a functional relationship between the transcription factors Pnt and Kah in midgut constrictions.; d) data resource of single cell genomics of mid embryonic stages with focus on mesodermal lineages.

Comments for the author

This work uses state-of-the-art gene expression analysis tools to identify novel target genes of the Alk signaling pathway. The story focuses on the Snail family transcription factor Kahuli. This result, together with some data on the interaction of Kah with Pnt in a less well understood biological process is interesting, also in a perspective of the function of Alk and Kah in vertebrates. The major part of this study provides a rich resource through its TaDa data sets, RNAseq data sets and the single cell genomics analyses in mid-stage embryos. The difficulty this manuscript deals with is that the functional analysis of Kah does not arrive at on a mechanistic level concerning the phenotype of midgut constrictions. It is also surprising that despite the expression profile of kah in founder cells and early visceral mesoderm, loss-of-function alleles did not show an apparent phenotype in the specification of these cell types. This issue, which may well be based upon functional redundancy or cooperation with other targets - such as the case for pnt in the constriction phenotypes suggests – remains unresolved. The results of the RNAseq experiment and the analysis of the ChIP data do provide an important entry point into further studies on the role of Kah and Pnt in this process and

© 2021. Published by The Company of Biologists under the terms of the Creative Commons Attribution License (https://creativecommons.org/licenses/by/4.0/).

the identification of Antp as one potential target is promising, but requires further studies. In summary this reviewer believes that in essence the exploratory nature of the data presented in this manuscript prevail and provide an excellent resource, but a more detailed analysis of candidate targets, like Kah will be required to advance the understanding of a developmental process.

Specific points:
(1) The authors claim that kah function in midgut constrictions is Alk-dependent. Does Alk have a phenotype in midgut constrictions? If that is not yet known, is it possible to use conditional alleles or RNAi to test this idea?
(2) It is surprising that - despite its expression pattern and the genetic evidence for kah being a target gene of Alk - kah mutants do not show a phenotype a well characterized Alk-dependent developmental process.
The TaDa approach identified 151 transcriptional regulators as candidate Alk target genes. Are there any candidates from this analysis, that could act in concert with kah? Does Pnt act in concert with Kah in visceral mesoderm specification?
(3) On the same line, is there any interaction between kah and any of the already known Alk target genes, in particular Hand or org-1, since these also do not show defects in founder cell formation.
(4) A general comment: Overall the manuscript is well presented, the data are of high quality and typos are rare. The exception of this assessment are the figure legends, which require proper editing in spelling and content (see below).

Minor comments:
- Page 32, line 3 ff: This figure legend contains many incomplete sentences and is fragmented, which makes it impossible to help understanding the figure properly.
- Page 32, lines 14-18; it is unclear what peaks are referred to in the first three sentences of legend for Figure 2.
- typo p34 line 6: figure legend of Figure 5. eliminate 'of'
- typo p34 line 7: figure legend of Figure 5. 'indicate' not 'indicates'
- typo p34 line 8: figure legend of Figure 5. ‘reveal’ - not - 'reveals'
- p35 line 3-4: figure legend of Figure 6. the description of panel B. is incomplete.
- Scale bars are missing in figures 1,3, 5, 6, and 8.

Reviewer 2

Advance summary and potential significance to field

The overarching goal of this study is to identify downstream genes of the RTK Alk in the founder cells of visceral muscles in Drosophila embryos and to define the function of one identified candidate gene, kahuli. Although Alk activity is essential for specifying these founder cells, none of the handful of downstream genes known to date confers such a fundamental role in this developmental pathway, which warrants new screens and reevaluations of published screens such as those performed by the authors of this manuscript. Specifically, these encompassed:
- A differential screen (targeted DamID) for RNA polymerase occupied genomic sites in the visceral mesoderm, using conditions of overactivated Alk (via overexpressed Jeb ligands) and reduced Alk activity (by expressing a dominant negative Alk version) vs. wildtype controls; however, due to the excessive variability of the data with overexpressed ligand, only those obtained with dominant negative Alk vs. wildtype were utilized).
- An scRNA-seq experiment with Hand-GFP-sorted visceral mesoderm cells.
- An RNAseq screen with mutants of one of the candidate Alk targets, namely the zinc finger domain encoding gene kah, vs. wildtype.
- Comparisons of ChIPseq data for Kah from ModENCODE with the author’s RNAseq data for kah
- Comparisons of ChIPseq data from ModENCODE for Kah with those of the Ets domain factor Pnt, which may undergo protein/protein interactions with Kah.
Additionally, a tagged version of Kah at the native locus as well as kah mutants were made and used to examine the expression and function of this gene in the visceral mesoderm.

© 2021. Published by The Company of Biologists under the terms of the Creative Commons Attribution License (https://creativecommons.org/licenses/by/4.0/).

Comments for the author

I acknowledge that a significant amount of work has been performed and each of the different approaches taken yielded some intriguing data. However, in my opinion none of the individual parts has been developed to a sufficient depth yet and the manuscript feels more like a preliminary presentation of a collection of screens rather than generating definitive insights. As pointed out below for some major examples, it is often nor clear how well the screens worked, whether some of the genes being presented were selected from the screening data based on objective criteria or rather by “picking and choosing”, and whether there are indeed profitable connections among the data obtained from the different screens.

1) TaDa (RNAPol occupancy) screen:
While it is true that Kah came out at or near the top of the lists (Table S1), many of the other high ranking genes code for general muscle differentiation genes, both with the bap and the twi driver (e.g., act57B, MLC2, MHC, TpnC73F, betaTUB60D). Similarly, among the transcription factor encoding genes, Mef2, a muscle differentiation factor expressed uniformly in all muscle progenitors, ranks at or near the top. This situation puts the ranking of Kah into perspective, particularly since, like Mef2 and the differentiaton markers, Kah is also expressed both in visceral and somatic muscle primordia. Most known FC-specific or -enriched genes, such as org-1, kirre, and Hand, rank much lower on the lists. Other transcription factor genes high on the lists do not make sense, because they are not even expressed in the visceral mesoderm (according to published data and the BDGP in situ expression database), so they could not be downregulated with dominant-negative Alk (e.g., twi, pros, sup, Doc1, ken, NFAT, hng3). Pdp1, shown by in situ hybridization in Fig. 3 and discussed in the Discussion, cannot be found in Table S1, so it is unclear where this came from. In any case, its expression has been reported to be specific for the endoderm, but not in the visceral mesoderm during the relevant stages (similarly for smt3; Fig.3). For the in situs in Fig. 3, early stage 12 embryos would be needed when Alk is active.
p.8, line 18: “At the individual gene level, occupancy of Dam-Pol II reveals similar binding profiles for twi.2xPEGAL4 and bap-GAL4 samples”. I do not see this to be the case for org-1 and Hand with bap>AlkDN and twi>AlkDN, respectively (Fig. 2E, F), nor for most genes in Fig. 3, and a statistical evaluation of this claim is not provided.

2) scRNAseq screen:
Encouragingly, the authors can separate visceral mesoderm cells and, when visceral mesodermal founder cells are expanded in vivo and sorted using Hand-GFP, can subdivide them into different clusters, all of which are clearly distinct from a cardiac mesoderm cluster also expressing Hand-GFP. However, the basis for identifying these six clusters with the terms given in Fig. 4E is unclear. For example, what is the difference between “Early visceral muscle”, “Early visceral mesoderm”, and “Early TVM”? How was a cluster defined as “Transition TVM to FCs”? How were “Founder cells” defined, since one of the few markers for these would be kirre, which is not present in Fig. G? And “Late visceral muscle” should be enriched for muscle differentiation genes, which are also not present in Fig. 4G? Unless the authors have additional data not included in the manuscript, assigning these specific names to these clusters is premature and misleading. For example, clustering of the data in Fig. 4 G similarly to that in 4C should be done to see whether there are any convincing biological correlates. Is there any overlap with the top genes from the TaDa screen, and in which cluster(s) are those enriched? In the current context, the main aims would seem to be the identification of a VM FC cluster.

3) RNaseq with kah mutants:
The stages used includes somatic mesodermal expression that is more widespread as compared to visceral mesodermal expression (and certainly FC expression), which may have limited the chances of success. Indeed, many of the "downregulated" genes in Fig. 7F for which data are available appear not to be expressed at all in embryos or not expressed at the proper stage or tissue (e.g., CG34267, LysX, CG12224, CG12420, CG13065; see BDGP).

4) modENCODE ChIPseq analysis for Kah:
Stating 20% of the genes being curated in BDGP as “midgut expression” (p. 15, line 15) is misleading, as “midgut “ usually refers to endoderm rather than visceral mesoderm. Cases in point are CG13321 and str, which are purely endodermal (see also BDGP) and therefore not in support of

© 2021. Published by The Company of Biologists under the terms of the Creative Commons Attribution License (https://creativecommons.org/licenses/by/4.0/).

a successful screen. If a percentage is given for genes with expression in a particular tissue such as visceral mesoderm, it needs to be calculated whether this is significantly different from random.

5) kah function and comparison with pointed:
The production of kah mutants and a tagged version is to be commended and should allow to define its developmental roles in the visceral and/or somatic mesoderm. But the images do not convincingly show a protein enrichment in VM founder cells and expansion or reduction with UAS-jeb and jeb mutants, respectively, perhaps because in normal embryos this enrichment is very transient (which should be documented). High magnifications with on-views of several neighboring segments should be presented here (e.g., Fig. 5J, J', where also many FCM's seem to be positive; and why is the nuclear factor Org-1 cytoplasmic here?). The unaffected expression of wg and dpp in kah mutants can be moved to a future supplement. The method and statistics for determining expanded numbers of visceral mesoderm nuclei needs to be explained (p. 14, line 2). The impact of the kah data would be strengthened if a target could be identified (from their various screens?) that mediates its function in the formation of the first midgut constriction. The expansion of Antp (which however would not be related to the first midgut constriction) needs to be documented better since weak expression posteriorly to the main domain in PS5/6 can also seen in the wildtype (Fig. 8I) and may be staining dependent. Likewise, the pnt data for a reduced first midgut constriction would be strengthened if a joint target for pnt and kah could be identified.
Altogether, although I see interesting potential in some of the stories presented, I would propose splitting up some of them, performing more in depth analyses on each, and publishing certain parts separately as more mature stories.

First revision

<u>Author response to reviewers' comments</u>

General response to both reviewers: We would like to thank the reviewers for their constructive comments and suggestions, and the editor for the opportunity to resubmit our manuscript, Mendoza- Garcia *et al.*, "DamID transcriptional profiling identifies the Snail/Scratch transcription factor Kahuli as an Alk target in the *Drosophila* visceral mesoderm". We have made a number of changes, corrections and additions that we feel have significantly strengthened the work. We have extensively reevaluated our single cell analysis as well as provided additional experimental analysis of the *Kah* mutant phenotype. Our point-by-point responses to the reviewers comments are detailed below, where we outline the changes we have made. Changes made in the text, excluding text editing to conform with length restrictions, are highlighted in red.

Reviewer 1 Advance Summary and Potential Significance to Field:

The Manuscript by Palmer and colleagues describes the identification of a member of the Snail family transcription factor Kahuli (Kah) as a target of the Anaplastic Lymphoma Kinase (Alk) pathway and its role in mesoderm development of the Drosophila embryo. The Palmer group had previously identified the Alk homologue in flies and discovered a crucial role of this pathway in visceral mesoderm development. Previous Work from the Palmer and other laboratories had identified a small group of Alk target genes and described their roles in the specification of visceral muscle founder cells, but a comprehensive analysis of the transcriptional targets of the Alk pathway remained elusive. In the present work, the authors employ an elegant combination of techniques to search for transcriptional targets of the Alk tyrosine receptor signaling pathway in the Drosophila embryo. They describe the Alk-dependent differential gene expression landscape by using a targeted DamID (TaDa) approach. Among the loci with significant changes in Dam-Pol-II binding, they reconfirmed genes previously known to be regulated by Alk providing a proof-of-principle. To provide further evidence for the specificity of the data set, they set out to examine the expression of the candidate target genes in distinct mesoderm cell lineages. Cluster analysis of single-cell RNAseq experiments revealed an enrichment of candidate Alk target genes in clusters of founder cells and early visceral mesoderm. The remaining of this manuscript focuses on the role of one new Alk target gene, kahuli (kah) coding a Snail family transcription factor. The

© 2021. Published by The Company of Biologists under the terms of the Creative Commons Attribution License (https://creativecommons.org/licenses/by/4.0/).

authors provide evidence that kah is expressed in the visceral mesoderm and that its expression is regulated by Alk signaling. Mutational analysis of kah demonstrated that kah does not affect early visceral mesoderm development, but is required for midgut constrictions in late embryonic stages. They found that the function of kah in this process is independent of Wg and Dpp, but Kah functions in concert with another Erk-dependent transcriptional regulator, Pointed (Pnt). The authors then describe an approach to identify Kah target genes by an RNA-seq and compared these data with Pnt ChIP-seq data, which identified a set of genes that may be regulated by Kah and Pnt together. Finally, sourcing publicly available ChIP data led them to identify a potential de novo binding motif for Kah and overlapping transcriptional targets using a Pnt ChIP data set. The authors propose a model in which Kah as a direct transcriptional target of the Alk pathway then acts together with Pnt to activate target genes involved in midgut constrictions and provide evidence for Antennapedia expression in the visceral mesoderm being regulated by Kah.
The advances this paper reports are a) the identification of candidate Alk target genes; b) identification and phenotypic analysis of Kah as an Alk target gene; c) a functional relationship between the transcription factors Pnt and Kah in midgut constrictions.; d) data resource of single cell genomics of mid embryonic stages with focus on mesodermal lineages.

Reviewer 1 Comments for the Author:
This work uses state-of-the-art gene expression analysis tools to identify novel target genes of the Alk signaling pathway. The story focuses on the Snail family transcription factor Kahuli. This result, together with some data on the interaction of Kah with Pnt in a less well understood biological process is interesting, also in a perspective of the function of Alk and Kah in vertebrates. The major part of this study provides a rich resource through its TaDa data sets, RNAseq data sets and the single cell genomics analyses in mid-stage embryos. The difficulty this manuscript deals with is that the functional analysis of Kah does not arrive at on a mechanistic level concerning the phenotype of midgut constrictions. It is also surprising that despite the expression profile of kah in founder cells and early visceral mesoderm, loss-of-function alleles did not show an apparent phenotype in the specification of these cell types. This issue, which may well be based upon functional redundancy or cooperation with other targets - such as the case for pnt in the constriction phenotypes suggests – remains unresolved. The results of the RNAseq experiment and the analysis of the ChIP data do provide an important entry point into further studies on the role of Kah and Pnt in this process and the identification of Antp as one potential target is promising, but requires further studies. In summary, this reviewer believes that in essence the exploratory nature of the data presented in this manuscript prevail and provide an excellent resource, but a more detailed analysis of candidate targets, like Kah will be required to advance the understanding of a developmental process.

Specific points:

(1) The authors claim that kah function in midgut constrictions is Alk-dependent. Does Alk have a phenotype in midgut constrictions? If that is not yet known, is it possible to use conditional alleles or RNAi to test this idea?
***Author's response**: We have now looked at this criticism from the reviewer carefully and have conducted a couple of experiments to address this. Since Alk is critical for founder cell specification, it is not possible to use Alk mutants to address this, as the reviewer also indicates. However, we have previously shown that Alk activity is required for Dpp expression in the VM (which is driven in the founder cells of PS7) by Alk signaling (please see below, data showing dpp mRNA in an Alk^{10} mutant extracted from Shirinian et al, PMID: 17286600, 2007; Fig 5):*

© 2021. Published by The Company of Biologists under the terms of the Creative Commons Attribution License (https://creativecommons.org/licenses/by/4.0/).

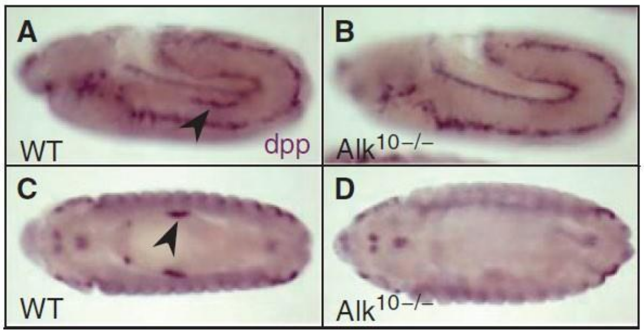

To address this experimentally, we have used a transgene that encoded the Alk extracellular domain that we have previously characterized as an effective dominant-negative Alk (Alk.DN). When overexpressed throughout the entire mesoderm (with the twi.2xPE-GAL4 driver) this blocks founder cell specification. However, when expressed in the VM with the bap-GAL4 driver this dominant- negative Alk protein is not able to block founder cell specification, likely due to not being produced at a high enough level in all cells fast enough to block Alk signaling. However, we now performed this experiment again, this time looking at midgut constriction and find that there are indeed defects in gut constriction when we block Alk signaling later in the VM. Of course, we cannot say whether this is related to Kah in any way, but it does support a role for Alk in the VM after the founder cell specification event and we have now included this experimental data in the revised manuscript (please see revised Fig 7I, and corresponding text in results and Fig. legend). Perhaps more interestingly, expression of the Alk ligand Jeb, using the twi.2xPE-GAL4 driver, also led to a severely disrupted gut phenotype, indicating that the VM is highly sensitive to both reduced and increased Alk singling activity. This experiment has also been included in the revised version of the manuscript (also revised Fig 7I, and corresponding text in the results and Fig. legend).

(2) It is surprising that - despite its expression pattern and the genetic evidence for kah being a target gene of Alk - kah mutants do not show a phenotype a well characterized Alk-dependent developmental process. The TaDa approach identified 151 transcriptional regulators as candidate Alk target genes. Are there any candidates from this analysis, that could act in concert with kah? Does Pnt act in concert with Kah in visceral mesoderm specification?
Author's response: The reviewer has a good point here and we have also considered this. Over the years, we and others have identified a number of interesting genes, including several transcriptional regulators, that are transcriptionally regulated by Alk (kirre, org-1, dpp, Hand) but that do not have a defect in founder cell specification in the early VM. Many of these have rather weak VM defects, for example, Hand and org-1. This is rather frustrating, and so far we have not been able to identify any factors that work together with Kah, other than the data we present here for Pnt. However, we aim to perform a more targeted screen of the transcriptional regulators identified in this TaDa analysis in both a WT and Kah background to try to address this. We have tried several candidates both before and during the revision process, but have so far been unsuccessful in identifying a novel factor in addition to Pnt that works with Kah in the VM.

(3) On the same line, is there any interaction between kah and any of the already known Alk target genes, in particular Hand or org-1, since these also do not show defects in founder cell formation.
Author's response: *We have now analysed a number of mutants alongside Kah, including Pnt, Org-1, H2O and Hand. We have analysed both single and double mutant combinations. In short, we have been unable to identify an interaction with any of these transcription factors, other than Pnt, and Kah. All mutant combinations that we have tested exhibit wild-type founder cell specification in the embryonic VM (at least, according to the markers we currently have to analyze this - HandC-GFP, Org-1, Vrp1, as well as morphology of the founder cells). Despite these rather extensive efforts, the only interaction we have been able to identify in a convincing manner is that of Kah and Pnt. In addition to the midgut constriction phenotypes observed in Kah/pnt transheterozygotes, we now also show by live imaging analysis that pnt/Kah double mutants show a strong and fully penetrant midgut phenotype, that is more severe than that of pnt alone. This*

© 2021. Published by The Company of Biologists under the terms of the Creative Commons Attribution License (https://creativecommons.org/licenses/by/4.0/).

new data is now included in Fig. 7G-H, and Supplementary Movies 8 and 9, and accompanying text.

(4)A general comment: Overall the manuscript is well presented, the data are of high quality and typos are rare. The exception of this assessment are the figure legends, which require proper editing in spelling and content (see below).
Author's response: *We apologise for this. We have now going through all the Figure legends carefully and edited them extensively.*

Minor comments:
-Page 32, line 3 ff: This figure legend contains many incomplete sentences and is fragmented, which makes it impossible to help understanding the figure properly.
-Page 32, lines 14-18; it is unclear what peaks are referred to in the first three sentences of legend for Figure 2.
-typo p34 line 6: figure legend of Figure 5. eliminate 'of'
-typo p34 line 7: figure legend of Figure 5. 'indicate' not 'indicates'
-typo p34 line 8: figure legend of Figure 5. 'reveal' - not - 'reveals'
-p35 line 3-4: figure legend of Figure 6. the description of panel B. is incomplete.
-Scale bars are missing in figures 1,3, 5, 6, and 8.
Author's response: *We thank the reviewer for picking up these errors and typos. We have now corrected these in the revised version of the manuscript.*

Reviewer 2 Advance Summary and Potential Significance to Field:

The overarching goal of this study is to identify downstream genes of the RTK Alk in the founder cells of visceral muscles in Drosophila embryos and to define the function of one identified candidate gene, kahuli. Although Alk activity is essential for specifying these founder cells, none of the handful of downstream genes known to date confers such a fundamental role in this developmental pathway, which warrants new screens and reevaluations of published screens such as those performed by the authors of this manuscript. Specifically, these encompassed:
- A differential screen (targeted DamID) for RNA polymerase occupied genomic sites in the visceral mesoderm, using conditions of overactivated Alk (via overexpressed Jeb ligands) and reduced Alk activity (by expressing a dominant negative Alk version) vs. wildtype controls; however, due to the excessive variability of the data with overexpressed ligand, only those obtained with dominant negative Alk vs. wildtype were utilized).
- An scRNA-seq experiment with Hand-GFP-sorted visceral mesoderm cells.
- An RNAseq screen with mutants of one of the candidate Alk targets, namely the zinc finger domain encoding gene kah, vs. wildtype.
- Comparisons of ChIPseq data for Kah from ModENCODE with the author's RNAseq data for kah
- Comparisons of ChIPseq data from ModENCODE for Kah with those of the Ets domain factor Pnt, which may undergo protein/protein interactions with Kah.

Additionally, a tagged version of Kah at the native locus as well as kah mutants were made and used to examine the expression and function of this gene in the visceral mesoderm.

Reviewer 2 Comments for the Author:
I acknowledge that a significant amount of work has been performed and each of the different approaches taken yielded some intriguing data. However, in my opinion none of the individual parts has been developed to a sufficient depth yet and the manuscript feels more like a preliminary presentation of a collection of screens rather than generating definitive insights. As pointed out below for some major examples, it is often nor clear how well the screens worked, whether some of the genes being presented were selected from the screening data based on objective criteria or rather by "picking and choosing", and whether there are indeed profitable connections among the data obtained from the different screens.

1) TaDa (RNAPol occupancy) screen:
While it is true that Kah came out at or near the top of the lists (Table S1), many of the other high ranking genes code for general muscle differentiation genes, both with the bap and the twi driver (e.g., act57B, MLC2, MHC, TpnC73F, betaTUB60D). Similarly, among the transcription

© 2021. Published by The Company of Biologists under the terms of the Creative Commons Attribution License (https://creativecommons.org/licenses/by/4.0/).

factor encoding genes, Mef2, a muscle differentiation factor expressed uniformly in all muscle progenitors, ranks at or near the top. This situation puts the ranking of Kah into perspective, particularly since, like Mef2 and the differentiaton markers, Kah is also expressed both in visceral and somatic muscle primordia. Most known FC-specific or -enriched genes, such as org-1, kirre, and Hand, rank much lower on the lists. Other transcription factor genes high on the lists do not make sense, because they are not even expressed in the visceral mesoderm (according to published data and the BDGP in situ expression database), so they could not be downregulated with dominant-negative Alk (e.g., twi, pros, sup, Doc1, ken, NFAT, hng3). Pdp1, shown by in situ hybridization in Fig. 3 and discussed in the Discussion, cannot be found in Table S1, so it is unclear where this came from. In any case, its expression has been reported to be specific for the endoderm, but not in the visceral mesoderm during the relevant stages (similarly for smt3; Fig.3). For the in situs in Fig. 3, early stage 12 embryos would be needed when Alk is active.
p. 8, line 18: “At the individual gene level, occupancy of Dam-Pol II reveals similar binding profiles for twi.2xPEGAL4 and bap-GAL4 samples”. I do not see this to be the case for org-1 and Hand with bap>AlkDN and twi>AlkDN, respectively (Fig. 2E, F), nor for most genes in Fig. 3, and a statistical evaluation of this claim is not provided.

Author’s response:

- *We appreciate the reviewer’s comment regarding the high ranking general muscle differentiation genes. We did not pursue them, since they are well characterized components of both the somatic and visceral musculature. We chose instead to pursue several genes that were uncharacterized, of which only Kah resulted in a VM phenotype. Moreover, Kah was also a strong candidate in both Twist and Bap datasets, which made it more interesting to us. Hits such as act57B, MLC2, MHC, TpnC73F and betaTUB60D may be very actively transcribed, making them more significant hits in terms of fold change in our TaDa dataset. It is not necessarily the case that a larger fold change means a more interesting hit, for instance, org-* 1 and Hand have clearly been shown to be Alk signaling targets, but are not particularly strong hits in our TaDa. This may reflect a tighter temporal and spatial expression of these genes. Overall, without conducting function analyses of a wide range of hits we cannot comment further on this point at this time.
- *Regarding transcription factors that do not make sense, we cannot currently explain the identification of these genes, however, TaDa is slightly different from RNA-seq and in situ in terms of what is being measured. One consideration is that TaDa only captures active transcription events (i.e., genes/DNA regions with elongating PolII), while RNA-seq and in situ measure total steady-state mRNAs that can include stable mRNAs from previous transcription events. TaDa readouts can be noisy and are somewhat less accurate for genes that are subjected to transcript stability controls.*
- *Regarding Pdp1, we also cannot find this as a significant hit in our TaDa dataset. It was identified in a pilot TaDa experiment with non-staged embryos. We can’t really explain this, and have now removed it from the revised version of our manuscript. We have now also carefully double checked though all other candidates included in the revised version.*
- *Regarding the in situs in Fig. 3, we have now provided a complementary analysis of these genes from our single cell datasets (which are aged at stages 10-13), in which we show theses candidates to be expressed in the VM (Supplementary Fig 5). In the case of CG11658 and Kah, their expression is highly enriched in VM, while for fax and smt3, expression is high in the VM but also present in a wide range of tissues. This data clearly supports our in situs and further integrates the TaDa with our single cell embryo dataset.*
- *Regarding the reviewers comment on the old p.8, line 18: “At the individual gene level, occupancy of Dam-Pol II reveals similar binding profiles for twi.2xPEGAL4 and bap-GAL4 samples”. In Fig. 3 we show TaDa profiles of genes that are differentially expressed between samples that overexpress either jeb or Alk.DN. These were selected form the list of significant genes from our TaDa analysis as outlined in the materials and methods, based on differentially peaks containing GATC sites in promoter regions. For graphical purposes we show logFC (comparing Jeb versus Dam only, and Alk.DN versus Dam only) of our TaDa reads aligned to the genome for these candidates in in Fig. 3. Here we have tried to state that in all cases we observe an increase of positive peaks upon Jeb*

© 2021. Published by The Company of Biologists under the terms of the Creative Commons Attribution License (https://creativecommons.org/licenses/by/4.0/).

overexpression that are downregulated (negative peaks) when overexpressing Alk.DN, pointing to the possibility of these genes to be regulated by Alk signaling. In each case shown in Fig 3, peaks are reduced in the presence of the Alk.DN. In brief, Fig 3 provides the reader with a qualitative analysis that visualises our dataset for these candidates, while the putative candidates identified in our TaDa experiments (as listed in Table S1), were identified based on a strict analysis considering GATC sites. We have now attempted to clarify this in the text for the reader as: "At individual gene levels, Dam-PolII occupancy revealed similar binding profile dynamics for jeb (positive peaks) and Alk.DN samples (negative peaks) (Fig. 3A-F)."

2) scRNAseq screen:
Encouragingly, the authors can separate visceral mesoderm cells and, when visceral mesodermal founder cells are expanded in vivo and sorted using Hand-GFP, can subdivide them into different clusters, all of which are clearly distinct from a cardiac mesoderm cluster also expressing Hand-GFP. However, the basis for identifying these six clusters with the terms given in Fig. 4E is unclear. For example, what is the difference between "Early visceral muscle", "Early visceral mesoderm", and "Early TVM"? How was a cluster defined as "Transition TVM to FCs"? How were "Founder cells" defined, since one of the few markers for these would be kirre, which is not present in Fig. G? And "Late visceral muscle" should be enriched for muscle differentiation genes, which are also not present in Fig. 4G? Unless the authors have additional data not included in the manuscript, assigning these specific names to these clusters is premature and misleading. For example, clustering of the data in Fig. 4 G similarly to that in 4C should be done to see whether there are any convincing biological correlates. Is there any overlap with the top genes from the TaDa screen, and in which cluster(s) are those enriched? In the current context, the main aims would seem to be the identification of a VM FC cluster.
***Author's response**: We appreciate the reviewers points here and have really tried to improve our single cell analysis and presentation, for both the whole embryo and the Hand-GFP sorted datasets.*
Regarding the whole embryo dataset, we have reanalyzed the heterogeneity of the dataset and defined clusters with canonical markers. For each cluster we have selected a pair of markers for visualization (feature plots). This now guides the reader though our analysis. These new analyses are now presented as Fig S3. To better define the relationship between the clusters we have perform phylogenetic tree analysis, which is now presented as Fig 4C, replacing the heatmap (which we have moved to the supplementary, Fig S3).
For the Hand-GFP dataset, we have considered very carefully the comments of the reviewer and have completely redone our analysis. Based on the reviewer's comments we now provide a much clearer presentation of this data and we thank the reviewer for their questions, which led to a much stronger analysis in the end. The reviewer is correct that the cardiac mesoderm is distinct and therefore easy to identify in this analysis (shown in Fig 4E-H in the revised version, where we also show a set of unique markers). We also considered that the 'early' and 'late' visceral muscle were rather easy to identify, based on the strong expression of e.g. components of the muscle machinery (such as up, Mlc2, Gyg, TpnC47D and Mhc). We now show this clearly in the revised version of Fig 4E-H. The 'late' visceral muscle cells clearly express markers such as sals, tn and slow, that are reported to be late expressed components of muscle, and this is now clearly shown for the reader in Fig 4H.
This left us with the tricky question of those cells that did not quite cluster with the founder cells in our analysis. After some head scratching, we remembered an old experiment, published from earlier work in our lab (Popoichenko et al, PMID: 23824577, 2013), in which we showed that a number of cells in the developing VM are pHis (phosphor-histone) positive. Moreover, this observed cell proliferation increases in the VM on overexpression of Jeb (please see extracted Figure 5 from this publication below).

© 2021. Published by The Company of Biologists under the terms of the Creative Commons Attribution License (https://creativecommons.org/licenses/by/4.0/).

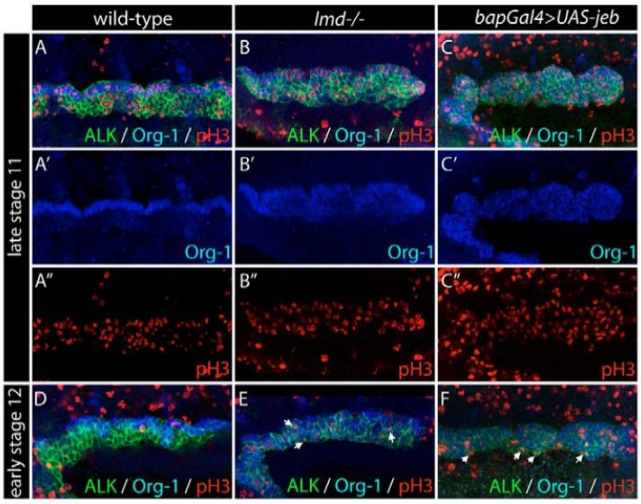

Fig. 5. Increased cell proliferation in the VM of ***lmd*$^{-/-}$ and** ***bapGal4>UAS-jeb*** **embryos.** (A-F) Alk (green), Org-1 (blue) and

This prompted us to ask whether the cells might be proliferating. Indeed, this is the case, and we now show that these cells, now termed as VM proliferating cells, strongly express FC markers, while exhibiting a strong proliferation cell cycle signature when subjected to GO analysis. We now include a panel of cell cycle/proliferation markers and their expression, together with the GO analysis, as Fig S4. A subset of 10 cell cycle/proliferation markers have been chosen to visualize this in the main figure to clarify for the reader (Fig 4H). Based on these new analyses, we have 5 clusters that we feel confident as identified (Cardiac mesoderm, VM Founder Cells, VM proliferating cells, Early Visceral Muscle and Late Visceral Muscle). These are represented now in a new UMAP (Fig 4E) which replaces our earlier UMAP analysis. We also show a correlation graph, in which the 'early' and 'late' visceral muscle cells clearly group together, as do the VM founder cells and VM proliferating cells, distinct from the cardiac mesoderm (Fig 4F).

We thank the reviewer again for bringing this up, as we feel this analysis and the revised figure has now much improved as a result.

3) RNaseq with kah mutants:
The stages used includes somatic mesodermal expression that is more widespread as compared to visceral mesodermal expression (and certainly FC expression), which may have limited the chances of success. Indeed, many of the "downregulated" genes in Fig. 7F for which data are available appear not to be expressed at all in embryos or not expressed at the proper stage or tissue (e.g., CG34267, LysX, CG12224, CG12420, CG13065; see BDGP).
Author's response*: The reviewer is correct that our Kah analysis is complicated by the expression in the somatic as well as the visceral mesoderm. We have now addressed the reviewers concerns by showing that differentially expressed genes identified in Kah mutants that overlap with the Kah-ChIP datset are enriched in the VM of our single cell embryo dataset (Fig 8D). As pointed out by the reviewer, there is also some enrichment for the somatic mesoderm, although this is not as strong.*
In addition, the reviewer highlights that the genes shown in old Fig 7F were not particularly informative. We have now replaced this with genes identified via a GO analysis of enriched pathways in genes differentially expressed in Kah mutants (Supplementary Fig 11). This highlighted components of several pathways, including Dpp signaling, that we now show in the revised version of this Fig (Fig 6G).

4) modENCODE ChIPseq analysis for Kah:
Stating 20% of the genes being curated in BDGP as "midgut expression" (p. 15, line 15) is misleading, as "midgut " usually refers to endoderm rather than visceral mesoderm. Cases in point are CG13321 and str, which are purely endodermal (see also BDGP) and therefore not in support of a successful screen. If a percentage is given for genes with expression in a particular tissue such as visceral mesoderm, it needs to be calculated whether this is significantly different

© 2021. Published by The Company of Biologists under the terms of the Creative Commons Attribution License (https://creativecommons.org/licenses/by/4.0/).

from random.
Author's response: *On reflection, we can see the reviewers point of view here and accept this criticism. We have attempted to correlate genes with our single cell embryo dataset, however, many genes are difficult to analyse due to low expression levels not being detectable by the 10x approach. Based on the reviewer's critique, we have now modified Fig 8 substantially, and integrated the ChIP data with both the RNA-seq analysis and Hand-GFP sorted single cell datasets. In modified Fig 8D, we now show that for detected genes in the single cell embryo dataset, there is a clear enrichment of Kah targets in the VM, as defined by our Kah RNAseq and Kah-ChIP dataset analyses.*

5) kah function and comparison with pointed:
The production of kah mutants and a tagged version is to be commended and should allow to define its developmental roles in the visceral and/or somatic mesoderm. But the images do not convincingly show a protein enrichment in VM founder cells and expansion or reduction with UAS-jeb and jeb mutants, respectively, perhaps because in normal embryos this enrichment is very transient (which should be documented). High magnifications with on-views of several neighboring segments should be presented here (e.g., Fig. 5J, J', where also many FCM's seem to be positive; and why is the nuclear factor Org-1 cytoplasmic here?). The unaffected expression of wg and dpp in kah mutants can be moved to a future supplement. The method and statistics for determining expanded numbers of visceral mesoderm nuclei needs to be explained (p. 14, line 2). The impact of the kah data would be strengthened if a target could be identified (from their various screens?) that mediates its function in the formation of the first midgut constriction. The expansion of Antp (which however would not be related to the first midgut constriction) needs to be documented better since weak expression posteriorly to the main domain in PS5/6 can also seen in the wildtype (Fig. 8I) and may be staining dependent. Likewise, the pnt data for a reduced first midgut constriction would be strengthened if a joint target for pnt and kah could be identified.

Author's response:

- *We have now included an additional experiment where we have looked at Kah^{OLLAS} in Alk mutants (Fig 5H-K'). This analysis shows that Kah is still expressed in Alk mutants, which is not surprising to us. However, Kah is enriched in founder cells, not only when analysed by in situ, but also when we stain for endogenous tagged Kah.OLLAS. We also now show strong enrichment of Kah mRNA in the founder cells of our Hand-GFP sorted single cell datasets (shown in revised Fig 5B). We do not claim that Alk is critical for Kah expression, but that it does regulate Kah expression to some extent. We have carefully checked through the manuscript in order to clarify this for the reader. These observations are similar to several other loci that Alk regulates, including Alk itself. For example, Alk mRNA is heavily expressed throughout the entire VM, but Alk expression is itself responsive to signaling through the pathway during FC specification (please see data using an Alk promotor construct (AlkE2.7) below, extracted from Mendoza-Garcia et al, PMID: 28369060, 2017; (E-F below).*

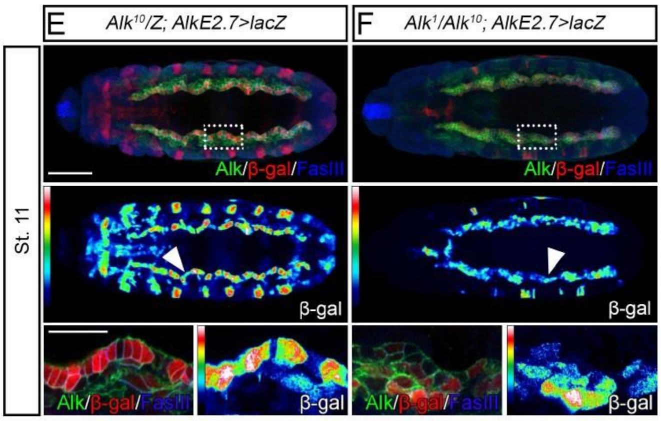

Corresponding Fig legend: *AlkE2.7 expression in the FCs is responsive to Alk signaling. lacZ expression in Alk1/Alk10 embryos is weaker when compared to Alk10 heterozygote balanced controls (arrowhead; compare β-gal heatmaps in E and F; note: epidermal β-gal expression*

© 2021. Published by The Company of Biologists under the terms of the Creative Commons Attribution License (https://creativecommons.org/licenses/by/4.0/).

in control (E) is due to presence of lacZ balancer; Alk protein is observed in Alk1/Alk10 animals (F) as these alleles encode non-functional Alk protein truncations detected with anti-Alk).)

- *In Fig 5, insets (old Fig 5J,J', new Fig F,F', as the reviewer notes, Org-1 is not cytoplasmic, we apologise. These insets are with Alk (green) and Kah^{OLLAS}(red). This is now corrected and clarified in the revised Fig 5 legend.*
- *As suggested by the reviewer, we have now moved the unaffected expression of wg and dpp in kah mutants to the supplementary information, as part of Fig S10.*
- *Regarding the method and statistics for determining expanded numbers of visceral mesoderm nublsi, this was done by measuring HandC-GFP nuclei. Samples were quantified as independent triplicates, n=10 for each sample (n=30 per genotype in total). Raw images were converted into binary format and nuclei were quantified using 3D nuclei counter package (Bolte and Cordelieres, 2006). To identify statistical differences between Kah mutants and controls (w1118), t-test analysis was performed. We have now clarified this in the Figure and legend (Fig 7F), as well as in the results text and the materials and methods.*
- *The reviewers is correct that if we could identify a target for Kah we that would mediate its function in the midgut constriction process that would indeed strengthen our data. We have made a great deal of effort here, but have not succeeded as yet. We aim to spend some time now systematically going through candidates and trying to identify some that will explain the Kah and Pnt phenotypes we observe. However, this is turning out to require a concerted effort and a more* comprehensive screen which we aim to carry out in future work. We wish that we could present such a candidate and explanation for the phenotype, but currently we cannot.
- *The expansion of Antp is rather clear to us, when observed in the microscope, and indeed is the only molecular hint we have of something that is abnormally expressed in the Kah mutants. However, we accept that the reviewer is not convinced and have removed this from the manuscript. We have mentioned Antp as a target of Pnt and Kah in the ChIP datasets and discuss it briefly in the discussion.*

Altogether, although I see interesting potential in some of the stories presented, I would propose splitting up some of them, performing more in depth analyses on each, and publishing certain parts separately as more mature stories.
Author's response: *We have really tried to address the concerns of reviewer 2 and to integrate our data better to provide a coherent story. In particular, we provide a more in depth analysis of our single cell datasets that are now integrated on multiple levels with our TaDa and Kah datasets. We feel that this has resulted in a much improved manuscript and hope that the reviewer agrees.*
Naturally, we would have loved to provide a mechanism by which Kah, and for that matter, Pnt and others regulate the process of midgut constriction. We do provide more experimental evidence of the Kah midgut constriction phenotype by live imaging, as well as show additional evidence of the Kah.Pnt interaction in this process. In addition, we also include data implicating Jeb/Alk signaling in later midgut development.

Second decision letter

MS ID#: DEVELOP/2021/199465

MS TITLE: DamID transcriptional profiling identifies the Snail/Scratch transcription factor Kahuli as an Alk target in the Drosophila visceral mesoderm.

AUTHORS: Patricia Mendoza-Garcia, Swaraj Basu, Sanjay Kumar Sukumar, Badrul Arefin, Georg Wolfstetter, Vimala Anthonydhason, Linnea Molander, Ezgi Uçkun, Henrik Lindehell, Christina Lebrero, Jan Larsson, Erik Lekholm Larsson, Mats Bemark, and Ruth H Palmer
ARTICLE TYPE: Research Article

© 2021. Published by The Company of Biologists under the terms of the Creative Commons Attribution License (https://creativecommons.org/licenses/by/4.0/).

I am terribly confused that your manuscript became stuck because one of the reviewers never returned their comments after accepting to review your revised manuscript. I looked at the other reviewer's comments and looked at the manuscript myself and in light of this I am happy to tell you that your manuscript has been accepted for publication in Development, pending our standard ethics checks.

Reviewer 1

Advance summary and potential significance to field

Their novel additional data now make a stronger and more consistent case for the biological roles of Alk and Kah in conjunction with Pnt and thus contribute to a better understanding of the developmental function of these genes in visceral muscle development and midgut constrictions. The lack of further genetic interactions among a range of Alk target genes suggests that the transcriptional network downstream of Alk is much more complex and requires further functional studies.

Comments for the author

The authors have attended to all my comments on their original manuscript. They added genetic data indicating a potential role of Alk signaling in midgut constrictions. Furthermore, the genetic interaction between Alk and Pnt has been extended to midgut constriction and live imaging data have been added to demonstrate these phenotypes. Their novel additional data now make a stronger and more consistent case for the biological roles of Alk and Kah in conjunction with Pnt and thus contribute to a better understanding of the developmental function of these genes in visceral muscle development and midgut constrictions. The lack of further genetic interactions among a range of Alk target genes suggests that the transcriptional network downstream of Alk is much more complex and requires further functional studies.

© 2021. Published by The Company of Biologists under the terms of the Creative Commons Attribution License (https://creativecommons.org/licenses/by/4.0/).
